# Combined analysis of host immune response, biofilm genes, and 16S rRNA detection in fracture-related infection: an observational cohort study

**DOI:** 10.5194/jbji-11-161-2026

**Published:** 2026-03-12

**Authors:** Melissa Depypere, Jonathan Sliepen, Jolien Onsea, Yves Debaveye, T. Fintan Moriarty, Elena Della Bella, Emmanuel André, Johan Van Weyenbergh, Willem-Jan Metsemakers

**Affiliations:** 1 Department of laboratory medicine, University Hospitals Leuven, 3000, Leuven, Belgium; 2 Department of Microbiology, Immunology and Transplantation, Laboratory of Clinical Bacteriology and Mycology, KU Leuven, Leuven, Belgium; 3 Department of Trauma Surgery, University Medical Center Groningen, Groningen, the Netherlands; 4 Department of Trauma Surgery, University Hospitals Leuven, 3000 Leuven, Belgium; 5 Department of Development and Regeneration, KU Leuven – University of Leuven, 3000 Leuven, Belgium; 6 Department of Intensive Care Medicine, University Hospitals Leuven, Leuven, Belgium; 7 AO Research Institute Davos, Davos, Switzerland; 8 Department of Microbiology, Immunology and Transplantation, Laboratory of Clinical and Epidemiological Virology, Rega Institute, KU Leuven, Leuven, Belgium

## Abstract

Fracture-related infection (FRI) is a serious complication in orthopaedic trauma that can lead to delayed union, nonunion, and poor clinical outcomes. A better understanding of the host immune response may provide valuable insights into the pathophysiology of FRI and may help identify genomic elements that contribute to the infection. This observational study compared immune responses between patients with FRI and non-infected controls using bone/tissue biopsies and sonication fluid, and it explored the possibility of detecting bacterial and biofilm genes using transcriptome profiling with hybridization technology (nCounter^®^ RNA hybridization technology). A total of 15 infected patients demonstrated significant upregulation of the innate immune pathway, including Toll-like receptor (TLR) signalling and the MyD88 cascade, suggesting an active immune response contributing to both infection control and bone resorption. Among the differentially expressed genes, PTGS2 (COX-2) showed the highest level of upregulation in the infection group. Bone biopsies showed enhanced chemokine (e.g. *CXCL1, CXCL2, CCL4/L1/L2*) signalling, with higher levels compared to tissue biopsies. Transcriptomic analysis identified bacterial transcripts in cases where conventional culture was negative, revealing potential cases of low-bacterial-load infections causing culture-negativity. Transcriptome profiling exposed distinct immune activation patterns in FRI and enabled the detection of pathogens missed by conventional culture. These findings call for larger, prospective studies to further explore the clinical utility of transcriptomics in understanding and managing FRI.

## 1 Introduction

Fracture-related infection (FRI) is one of the most challenging complications in orthopaedic trauma surgery. The incidence of FRI ranges from 1 % after closed low-energy fractures to up to 30 % in complex open tibia fractures worldwide (Papakostidis et al., 2011; Metsemakers et al., 2023; Boxma et al., 1996). Globally, approximately 1.8 million FRI cases are diagnosed each year (Metsemakers et al., 2023). The socio-economic burden is enormous, with costs that are 4 times higher than those of periprosthetic joint infection (PJI) (Darouiche, 2004; Moriarty et al., 2022).

A consensus definition for the diagnosis of FRI was published in 2018, providing clear diagnostic criteria that are pathognomonic for FRI. Two levels of diagnostic certainty were outlined: confirmatory criteria and suggestive criteria (Metsemakers et al., 2018). The presence of one or more confirmatory signs warrants immediate treatment of FRI, while suggestive signs should prompt further investigation by the medical team. A diagnostic algorithm for suspected FRI can be applied, based on key principles such as interpretation of clinical signs, serum tests, imaging, microbiology, and histology (McNally et al., 2020).

Implantation of a fracture fixation device increases the risk of infection and the formation of biofilm on the implant (Wildemann et al., 2021). *Staphylococcus aureus* followed by coagulase-negative staphylococci and Enterobacterales are the predominant pathogens in these infections (Depypere et al., 2022). There is general agreement that biofilms are the main reason for persistent and highly destructive infections (Costerton et al., 1999). It is stated that younger biofilms are associated with a more pro-inflammatory response, with neutrophils and M1-polarized macrophages releasing pro-inflammatory cytokines such as *IL1* β , *IL6*, and *TNF*- α in an attempt to clear the infection, while mature biofilms are associated with an immune microenvironment that is more anti-inflammatory (*IL4, IL10*) and immune-suppressive, which supports bacterial persistence, limiting tissue damage but also allowing chronic infection (Seebach and Kubatzky, 2019; Ciornei et al., 2010; Kaya et al., 2021). In addition, new bone deposition by osteoblasts is reduced locally, leading to decreased mineralization and increased apoptosis of osteoblastic cells (Marriott, 2013).

NanoString nCounter^®^ (NanoString^®^, Seattle, WA, USA) is a technology that uses a rapid, multiple, fluorescence-based hybridization approach of gene expression analysis. Unlike sequencing-based methods, it does not require amplification or cDNA/library preparation, making the process technically simpler and faster (Goytain and Ng, 2020). This technology has already been widely applied in infectious disease research (Feys et al., 2022; Fukutani et al., 2015; Andrade et al., 2017), but it has not yet been used for the detection of bacterial pathogens or biofilm-associated genes, representing a novel application in this context.

We conducted a “proof-of-concept” study to assess the applicability of nCounter^®^ RNA hybridization technology for transcriptome profiling in patients suspected of having FRI. Using this technology, we analysed tissue and bone biopsy samples along with sonication fluid. The primary goal was to compare the immune responses in patients with FRI, as defined by the consensus criteria (Metsemakers et al., 2018), to those in a non-infected control group consisting of fracture patients with a clinical suspicion of infection who underwent surgical revision but in whom infection was finally excluded based on negative cultures, imaging, clinical assessment, and multidisciplinary team consensus. A secondary objective was to determine whether this method could detect bacterial 16S ribosomal RNA (16S rRNA) and biofilm-related genes.

## 2 Methods

### 2.1 Study design and study population

In this observational, proof of concept study, we prospectively included 34 patients who required musculoskeletal trauma surgery at the University Hospitals Leuven, Belgium, between November 2018 and April 2021. Out of these patients, 16 were diagnosed with an infection according to the FRI consensus definition and 18 were included in the control group. Control patients were selected from the same clinical population as the FRI group. All patients presented with pain or nonunion for which infection was suspected and therefore underwent surgical revision and intraoperative sampling. Only those patients for whom infection was excluded based on negative culture results, imaging, clinical findings, and multidisciplinary team consensus were included as controls. These controls were selected consecutively from this subgroup of non-infectious cases during the same study period, ensuring similar clinical presentation and indication for surgery. In cases where there were only suggestive findings, the decision of the multidisciplinary team was considered final.

In addition to the standard 4–6 biopsy samples collected during surgery for conventional culture, we collected one additional tissue sample, bone biopsy, or sonication fluid from all patients. The additional samples were immediately frozen at -80 °C in cryovials without preservatives. Conventional tissue/bone biopsies were inoculated in Wilkins-Chalgren broth at 37 °C for 10 d. When visually cloudy, broths were subcultured on a standard array of agar plates. After 24–48 h of incubation, isolates were identified by matrix-assisted laser desorption ionization time-of-flight (MALDI-TOF) mass spectrometry (Bruker UK Ltd.). Antimicrobial susceptibility testing was performed using VITEK^®^2 (BioMérieux, Marcy l'Etoile, France). Demographic and clinical data were collected from the patient electronic medical records. Ultimately, patients were classified as an infected or non-infected control group based on the abovementioned criteria aligned with the FRI consensus definition. Patients initially suspected of infection but in whom FRI was ultimately excluded by the multidisciplinary team comprised the control group (Metsemakers et al., 2018). Table 1 gives an overview of the confirmatory and suggestive criteria of the consensus definition.

**Table 1:** Confirmatory and suggestive criteria for the diagnosis of FRI (Metsemakers et al., 2018).

Confirmatory criteria	Suggestive criteria
Clinical signs – Fistula – Sinus – Wound breakdown – Purulent drainage or the presence of pus	Clinical signs – Local/systemic (e.g. local redness, swelling, fever, pain) – New-onset joint effusion – Persistent, increasing, or new onset wound drainage
Microbiology – Phenotypically indistinguishable pathogens, identified by culture from at least 2 separate deep-tissue/implant specimens	Laboratory signs – Increased serum inflammatory markers (WBC count, CRP)
Histopathology – Presence of microorganisms in deep-tissue specimens, confirmed by using specific staining techniques for bacteria and fungi – Presence of > 5 PMNs/HPF in chronic/late-onset cases	– Radiological and/or nuclear imaging signs – Microbiology pathogenic microorganisms identified from a single deep-tissue/implant specimen

PMN: polymorphonuclear neutrophils; HPF: high-power field; WBC count: white blood cell count; CRP: C-reactive protein.

### 2.2 Procedures

#### 2.2.1 RNA extraction and nCounter^®^ analysis

RNA was extracted from the dedicated frozen tissue/bone samples and sonication fluid using the MagMax Viral and Pathogen Nucleic Acid Isolation kit (Applied Biosciences, Thermo Fisher), following manufacturer's instructions, and was hybridized (65° C for 24 h) to a Host Response Panel of 773 genes covering the host immune response to infectious diseases and 12 internal reference genes for data normalization, designed by NanoString Technologies (nCounter Human Host Response Panel, catalogue number 531-115000449) (cf. Table S1 in the Supplement: Overview of target genes included in the transcriptomic panel). To tailor the panel to our research objectives, we supplemented the predesigned panel with 17 additional, custom-selected probes targeting bacterial biofilm genes and 16S rRNA of five bacteria regularly involved in FRIs (*Pseudomonas aeruginosa*, *Enterobacter cloacae*, *Staphylococcus aureus*, *Staphylococcus epidermidis*, *Enterococcus faecalis*) (Table S2: Additional probes spiked into the panel).

For the biofilm genes, we selected key genes for each organism. For Staphylococci, we focused on *intercellular adhesins icaA, icaB, and icaC*, which synthesize polysaccharide intercellular adhesin (PIA), essential for adhesion, aggregation, and attachment to abiotic surfaces. Additionally, we included *ClfA and ClfB* (clumping factors) and *FnbA/B* (fibronectin-binding proteins), all of which are surface proteins critical for adherence to host tissues (Francis et al., 2024; Kavanagh et al., 2018). For *P. aeruginosa*, we targeted *lectins A and B (lecA/lecB)* – soluble outer-membrane proteins that aid in cell and exopolysaccharide retention within biofilms, promoting robust adhesion (Passos Da Silva et al., 2019). We also examined the *cupA gene cluster* (cupA1 through cupA5), with *cupA1* encoding a significant fimbrial subunit and *cupA4* producing a typical adhesin, both crucial for biofilm structure and stability (Vallet et al., 2001). For *E. cloacae*, we selected *adrA*, which promotes the syntheses of exopolysaccharides, an essential component of the biofilm matrix (Muriel et al., 2019); *csgB*, which facilitates adhesion to the biofilm through Curli fibres (Yan et al., 2020); and *flgM* and *fliA*, which regulate flagellar activity, allowing transition from a planktonic to a biofilm state (Dudin et al., 2014; Xu et al., 2014).

Raw data were processed using nSolver 4.0 software (Nanostring Technologies) sequentially correcting three factors: technical variation between samples (6 positive control RNAs, spiked-in for each individual sample to control for technical variation), background correction (8 negative control RNAs, spiked-in for each individual sample to control for background variation), and RNA content by adjusting for the 12 housekeeping genes *(ACTB, B2M, GAPDH, HPRT1, RPL13A, TBP, PPIA, GUSB, PGK1, SDHA, YWHAZ, RPLP0)*, followed by normalization using logarithmic transformation.

#### 2.2.2 Data analysis

No sample size calculation was performed for this “proof of concept” study. Demographic and clinical data were collected and analysed using RStudio (version 2024.09.0 + 375) for Windows. The data were reported using standard descriptive statistics, including counts and percentages to report proportions, mean and standard deviation (SD) for normally distributed continuous variables, and median and inter-quartile range (25th percentile–27th percentile ( $p_{25}$ – $p_{75}$ )) for non-parametric variables. p values were calculated using the $\chi^{2}$ or Fisher's exact test for categorical variables and the Mann–Whitney U test for continuous variables. Gene expression was analysed using ROSALIND^®^ (https://rosalind.onramp.bio/, last access: 30 May 2024), with a HyperScale architecture developed by ROSALIND, Inc. (San Diego, CA). Normalization, fold changes, and p values were calculated using criteria provided by Nanostring. ROSALIND^®^ follows the nCounter Advanced Analysis protocol of dividing counts within a sample by the geometric mean of the normalizer probes from the same sample. Volcano plots were constructed using GraphPad Prism 10.2.3. Pathway enrichment was performed using WebGestalt (WEB based Gene Set Analysis toolkit, 2017) on the normalized expression data generated by ROSALIND^®^, using Reactome canonical pathway (Version 66, September 2018) (Gillespie et al., 2022). We accessed the enrichment scores (ESs) for pathways with a minimum of 5 genes. ES reflects the degree to which a set is overrepresented. The normalized enrichment score (NES) is applied to account for the size of the set, followed by calculating the false discovery rate (FDR) for control of the false positives. Dot plots were generated using R 4.3.0 with packages ggplot2, glue, and dplyr.

Statistical testing was corrected for multiple testing by use of the more stringent Benjamini–Hochberg false discovery rate (FDR) method for analysis of gene expression (nCounter^®^) and pathways (gene set enrichment analysis). Differentially expressed genes (DEGs) were identified at an adjusted p value of less than 0.05. Where no DEGs could be identified with an adjusted p value < 0.05, uncorrected p values of <0.05 were used. For pathway analysis, the recommended 25 % significance level for FDR was applied (Subramanian et al., 2005). For interpretation of bacterial and biofilm gene expression, we applied fold-change expression of each gene as compared to the mean of four negative controls used for background correction. We used a fold change of 3 as a stringent cut-off for positivity above background.

## 3 Results

### 3.1 Population characteristics

A total of 34 patients were included in this study: 16 were diagnosed with an infection according to the FRI consensus definition (Table 1), and 18 were included in the control group (Metsemakers et al., 2018). A total of 14 tissue biopsies, 15 bone biopsies, 1 sonication fluid, and 4 samples of unknown origin (bone or tissue) were included. A detailed overview is shown in Table 2. There were insufficient data on preoperative C-reactive protein (CRP) and white blood cell (WBC) levels to represent these data in the table. There were 68.8 % and 50 % males in the infection and control group respectively. The median age was 51 years ( $p_{25}$ – $p_{75}$ : 29–65) in the infection group and 51 years ( $p_{25}$ – $p_{75}$ : 37–71) in the control group. *S. aureus* was the predominant pathogen in the infection group ( n=7 ; 43.8 %). Unhealed fractures were the dominant healing status and occurred equally in both groups.

**Table 2:** Baseline patient characteristics.

	Infection group n=16 (%)	Control group n=18 (%)	p value
Sex (male)	11 (68.8)	9 (50.0)	0.268
Age (years) median ( $p_{25}$ – $p_{75}$ )	51 (29–65)	51 (37–71)	
BMI (kg m^−2^) median ( $p_{25}$ – $p_{75}$ )	27.5 (24.7–34.0)	25.7 (22.6–29.0)	0.161
Diabetes	1 (6.3)	5 (27.8)	0.180
ASA			0.906
I = healthy	3 (18.8)	5 (27.8)	
II = mild systemic disease	8 (50.0)	7 (38.9)	
III = severe systemic disease	5 (31.3)	6 (33.3)	
Anatomical localization			0.271
Humerus	4 (25.0)	1 (5.6)	
Clavicle	2 (12.5)	0 (0)	
Radius/ulna	1 (6.3)	2 (11.1)	
Femur	4 (25.0)	5 (27.8)	
Tibia	3 (18.8)	7 (38.9)	
Fibula	0 (0)	1 (5.6)	
Pelvis/acetabulum	0 (0)	1 (5.6)	
Calcaneus	1 (6.3)	0 (0)	
Patella	1 (6.3)	0 (0)	
Carpal bone	0 (0.0)	1 (5.6)	
Fracture type			0.429
Open	5 (31.3)	3 (16.7)	
Closed	11 (68.8)	15 (83.3)	
Time since trauma (days) median ( $p_{25}$ – $p_{75}$ )	279 (55–1297)	397 (106–746)	0.704
Clinical presentation			
Confirmatory signs			
Fistula/sinus tract	7 (43.8)	0 (0)	**0.002** ^a^
Wound breakdown	0 (0)	0 (0)	–
Purulent discharge	7 (43.8)	0 (0.0)	**0.002** ^a^
Suggestive signs			
Redness	5 (31.3)	3 (16.7)	0.429
Pain	7 (43.8)	11 (61.1)	0.311
Swelling	6 (37.5)	5 (27.8)	0.545
Fever ( >38.3 °C)	2 (12.5)	1 (5.6)	0.591
Local warmth	2 (12.5)	2 (11.1)	1.000
Joint effusion	1 (6.3)	1 (5.6)	1.000
Wound drainage	4 (25.0)	0 (0)	**0.039** ^a^
Confirmatory pathogen conventional culture^b^			
*Staphylococcus aureus*	7 (43.8)	0 (0)	
*Staphylococcus epidermidis*	2 (12.5)	0 (0)	
*Staphylococcus capitis*	1 (6.3)	0 (0)	
*Staphylococcus caprae*	1 (6.3)	0 (0)	
*Enterobacter cloacae*	1 (6.3)	0 (0)	
*Enterococcus faecalis*	1 (6.3)	0 (0)	
*Pseudomonas aeruginosa*	1 (6.3)	0 (0)	
*Bacillus* spp.	1 (6.3)	0 (0)	
*Cutibacterium acnes*	1 (6.3)	0 (0)	

p values of <0.05 are highlighted in bold.

**Table 2:** Continued.

	Infection group n=16 (%)	Control group n=18 (%)	p value
Suggestive pathogen conventional culture^c^			
*Staphylococcus lugdunensis*	1 (6.3)	0 (0)	
*Staphylococcus epidermidis*	0 (0)	2 (11.1)	
*Enterobacter cloacae*	1 (6.3)	0 (0)	
*Bacillus* spp.	0 (0)	2 (11.1)	
Culture-negative	1 (6.3)	14 (77.8)	
Fracture healing status at time of surgery			1.000
Healed	2 (12.5)	2 (11.1)	
Unhealed	14 (87.5)	16 (88.9)	
Antibiotics <2 weeks prior to sampling	3 (18.8)	0 (0)	0.094

ASA: American Society of Anesthesiology; spp.: species; BMI: body mass index. Due to an insufficient number of available preoperative C-reactive protein (CRP) and white blood cell (WBC) measurements, these data were not presented. ^a^ Statistically significant at p<0.05 . ^b^ Phenotypically indistinguishable pathogens, identified by culture from at least 2 separate deep-tissue/implant specimens. ^c^ Microbiology pathogenic microorganisms identified from a single deep-tissue/implant specimen

### 3.2 Analysis of bacterial gene expression

Analysis failed for three samples because of a QC error: two negative samples in the control group and one sample in the infection group with growth of *S. aureus* by conventional culture. The infected group was composed of patients with positive cultures for *S. aureus* ( n=6 ), *Staphylococcus epidermidis* ( n=2 ), *Enterococcus faecalis* ( n=1 ), *Enterobacter cloacae* ( n=2 ), *Pseudomonas aeruginosa* ( n=1 ), and species not covered in our transcriptomic panel, as no corresponding probe was included for their detection ( n=5 ). One patient in the infected group was considered to be false culture-negative. This patient developed a sinus tract (confirmatory criteria for infection) shortly after implantation of the fixation plate.

Table 3 shows an overview of the 16S bacterial transcripts among the infected group ( n=15 ) and the control group ( n=16 ). In the infection group, using a fold change (above background) of 3 as threshold for 16S rRNA transcripts could correctly predict culture outcome for 5 out of 6 *S. aureus*, 1 out of 1 *E. faecalis*, 1 out of 2 *E. cloacae*, and 0 out of 1 *P. aeruginosa* infections. The patient who was false culture-negative (patient 6) showed a positive *S. aureus* 16S rRNA transcript. Transcriptome analysis uncovered concomitant infection with *E. cloacae* and *P. aeruginosa* for patient 7, in contrast with conventional culture, showing only growth of *E. cloacae*. We observed a cross-hybridization between the probes for *S. aureus (NR_118997.2)* and *S. epidermidis (NR_036904.1)*, which was confirmed in the NCBI 16S BLAST database. Given the strong correlation between the *S. aureus* signal and corresponding culture results, we decided not to include *S. epidermidis* results, as these provided limited additional value. Consequently, no data for *S. epidermidis* are shown in Table 3. The control group, composed of patients with negative cultures (or contamination), showed negative 16S rRNA transcripts among the majority of the patients ( n=12 ), while a positive signal for *S. aureus* was retrieved using the threshold of a fold change of 3 among four patients.

The detection of biofilm gene transcripts showed lower sensitivity compared to the 16S transcripts, as they could only be clearly distinguished above background in one MRSA infection (patient 29), which also showed the highest 16S rRNA signal. In all other samples, the biofilm-related transcripts lacked specificity and displayed lower expression. However, we hypothesized that these lower signals most probably represent genuine biofilm gene expression, since they were also detected in purified bacterial colonies from clinical isolates with biofilm properties (data not shown). In addition, no cross-hybridization with host genes was observed when tested in uninfected whole blood samples (data not shown). See Table S3 for a more detailed Table 3 with the addition of biofilm genes and data for *S. epidermidis*.

**Table 3:** Overview of all included samples ( n=34 ), showing results from both conventional methods and nCounter^®^ analysis for 16S rRNA. The table shows raw counts with a fold change of 3 as a stringent cut-off for positivity above background.

Group	Patient	Conventional culture	Type of sample	*Staphylococcus aureus*	*Enterococcus faecalis*	*Enterobacter cloacae*	*Pseudomonas aeruginosa*	16S rRNA
Infected	2	*S. aureus*	*Unknown*	251	1	2	1	*S. aureus*
	3	*S. aureus*	*Unknown*	25	1	0	0	*S. aureus*
	13	*S. aureus*	*Tissue*	3	2	2	1	*S. aureus*
	25	*S. aureus*	*Bone*	2	1	1	1	*Negative*
	29	*MRSA*	*Tissue*	29 147	2	0	0	*S. aureus*
	32	*S. aureus*	*Tissue*	4	0	1	1	*S. aureus*
	6	*Negative* ^c^	*Tissue*	8	0	2	1	*S. aureus*
	10	*S. epidermidis / S.capitis / S. caprae / Bacillus sp.*	*Tissue*	2	1	1	1	*Staphylococcus sp.*
	12	*E. faecalis*	*Bone*	1	4	1	1	*E. faecalis*
	14	*P. aeruginosa*	*Bone*	2	0	1	1	*Negative*
	15	*S. lugdunensis*	*Tissue*	2	1	1	1	*No probe included*
	19	*S. epidermidis*	*Tissue*	3	1	1	1	*Staphylococcus sp.*
	7	*E. cloacae*	*Bone*	1	1	149	38	*E. cloacae/P. aeruginosa*
	18	*E. cloacae*	*Tissue*	0	1	2	0	*Negative*
	33	*C. acnes* ^b^	*Unknown*	2	0	2	2	*No probe included*
Control	1	*Negative*	*Bone*	1	1	1	1	*Negative*
	4	*Negative*	*Tissue*	8	2	2	2	*S. aureus*
	5	*Negative*	*Bone*	1	1	1	1	*Negative*
	8	*S. epidermidis* ^a^	*Tissue*	3	1	1	1	*S. aureus*
	9	*Negative*	*Bone*	1	1	1	1	*Negative*
	11	*Negative*	*Bone*	2	1	1	1	*Negative*
	16	*S. epidermidis/Bacillus sp.* ^a^	*Bone*	1	0	1	1	*Negative*
	17	*Bacillus species* ^a^	*Bone*	0	0	0	0	*Negative*
	20	*Negative*	*Bone*	2	1	1	1	*Negative*
	22	*Negative*	*Bone*	21	1	1	1	*S. aureus*
	24	*Negative*	*Bone*	7	1	1	1	*S. aureus*
	26	*Negative*	*Tissue*	1	1	0	0	*Negative*
	27	*Negative*	*Bone*	2	2	1	1	*Negative*
	30	*Bacillus cereus* ^a^	*Bone*	1	0	1	1	*Negative*
	31	*Negative*	*Tissue*	1	1	1	1	*Negative*
	34	*Negative*	*Sonicate*	1	0	1	1	*Negative*
Failed analysis	21	*S. aureus*	*Unknown*					
	23	*Negative*	*Tissue*					
	28	*Negative*	*Tissue*					

*S. aureus*: *Staphylococcus aureus*; *S. epidermidis*: *Staphylococcus epidermidis*; *S. caprae*: *Staphylococcus caprae*; *S. capitis*: *Staphylococcus capitis*; *C. acnes*: *Cutibacterium acnes*; MRSA: methicillin-resistant *Staphylococcus aureus*; *E. cloacae*: *Enterobacter cloacae*; *P. aeruginosa*: *Pseudomonas aeruginosa*; sp: species. ^a^ Considered to be contaminants (classified as suggestive pathogens, but considered to be contaminants after evaluation by the multidisciplinary team). ^b^ No probes included. ^c^ Considered to be a false-negative culture result. No data were shown for patients 21, 23, and 28 because the transcriptomic analyses for these samples failed due to QC error.

### 3.3 Analysis of host gene expression pathways

**Figure 1:**
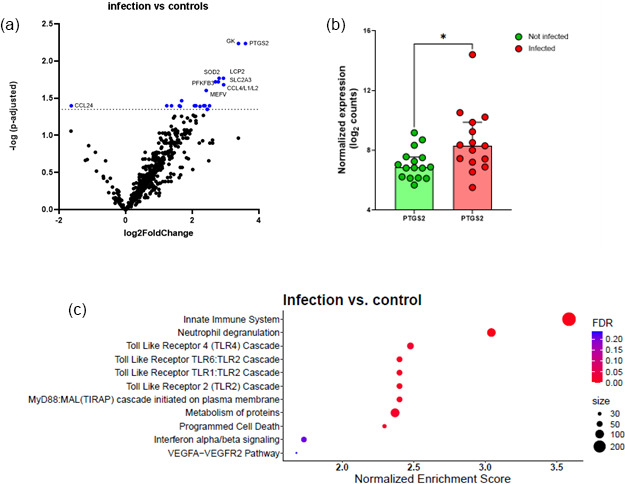
Comparison between infected FRI patients and a non-infected control group. **(a)** Volcano plot identifying differentially expressed genes (DEGs) between patients with confirmed infection ( n=15 ) and non-infected controls ( n=16 ) in tissue/biopsy samples and sonication fluid. The x axis reports the Log2FoldChange, and the y axis reports the -Log10 of the p -adjusted. Genes with |Log2 fold change |>1 (generated by nSolver/ROSALIND for visualization of differential expression) and -Log10 ( p -adjusted) > 1.3 (equivalent to p -adjusted < 0.05) were considered significantly differentially expressed. **(b)** Comparison between non-infected controls and infected patients on PTGS2 normalized expression. There is a significant increase in PTGS2 in the infection group compared to matched controls; bars represent the median with 95 % CI ( p=0.017 , Mann–Whitney test). Each circle represents a single (red, n=15 ) patient or matched control (green, n=16 ). ^*^ p<0.05 . **(c)** Bubble plot of Reactome pathway enrichment analysis for DEGs. Significantly enriched pathways (FDR < 0.25) are displayed. Bubble size represents the number of DEGs associated with each pathway, and colour indicates the statistical significance (FDR). Pathways are ranked by normalized enrichment score. *Abbreviations:* DEG = differentially expressed gene; FDR = false discovery rate; log2 = log base 2; -Log10 : negative log base 10; PTGS2 = prostaglandin-endoperoxide synthase 2; VEGFA = vascular endothelial growth factor A.

Overall, after FDR correction, transcriptome analysis identified 21 significantly upregulated DEGs and 1 downregulated gene (*CCL24*) when comparing the infectious group with the control group (cf. Table S4). Figure 1a shows the volcano plot of the DEGs. Figure 1b shows significant increase in *PTGS2* in the infection group compared to matched controls.

A large group of genes with increased expression in response to infection belong to immunological pathways including innate immunity, neutrophil degranulation, and Toll-like receptor (*TLR)* pathway. An additional pathway found to be upregulated was the *MyD88* (*TIRAP*) cascade associated with programmed cell death (Fig. 1c).

**Figure 2:**
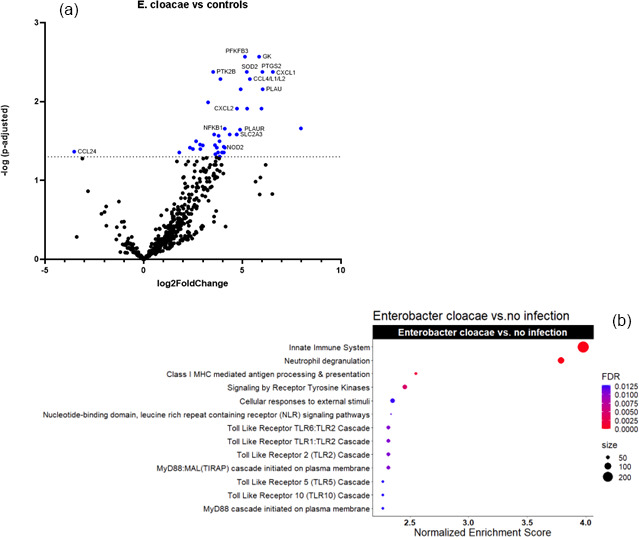
Comparison between *E. cloacae*-infected FRI patients and a non-infected control group. **(a)** Volcano plot showing DEGs between *E. cloacae*-infected patients ( n=2 ) and non-infected controls ( n=16 ). The x axis reports the Log2FoldChange, and the y axis reports the -Log10 of the adjusted p value. Genes with |Log2 fold change |>1 (generated by nSolver/ROSALIND for visualization of differential expression) and -Log10 ( p -adjusted) > 1.3 (equivalent to adjusted p value < 0.05) were considered significantly differentially expressed. **(b)** Bubble plot of Reactome pathway enrichment analysis for DEGs comparing *E. cloacae* infections and controls. Significantly enriched pathways (FDR < 0.25) are displayed. Bubble size represents the number of DEGs associated with each pathway, and colour indicates the statistical significance (FDR). Pathways are ranked by normalized enrichment score. Notably, genes involved in MHC class I antigen presentation, which mediate intracellular bacterial recognition, were upregulated in *E. cloacae* infection. *Abbreviations*: DEG = differentially expressed gene; FDR = false discovery rate; log2 = log base 2; -Log10 : negative log base 10; MHC = major histocompatibility complex.

**Figure 3:**
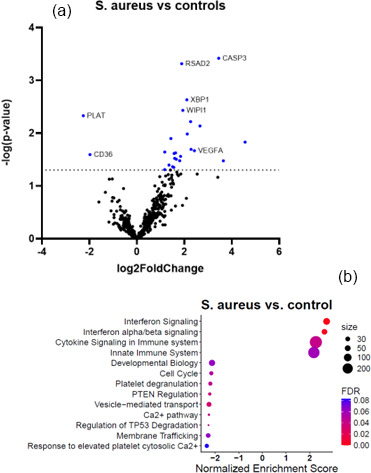
Comparison between *S. aureus*-infected FRI patients and a non-infected control group. **(a)** Volcano plot showing DEGs between *S. aureus*-infected patients ( n=6 ) and non-infected controls ( n=16 ). The x axis reports the Log2FoldChange, and the y axis reports the -Log10 of the p value. Genes with a Log2FoldChange > |1| (generated by nSolver/ROSALIND for visualization of differential expression) and -Log10 ( p value > 1.3) (equivalent to a p value < 0.05) were considered to be differentially expressed. **(b)** Bubble plot of Reactome pathway enrichment analysis for DEGs in the comparison between *S. aureus* infection and non-infected controls. Significantly enriched pathways (FDR < 0.25) are displayed. Bubble size represents the number of DEGs associated with each pathway, and colour indicates the statistical significance (FDR). Pathways are ranked by normalized enrichment score. Downregulation of platelet degranulation and calcium-signalling pathways was observed, while VEGFA-related angiogenesis genes were upregulated. *Abbreviations:* DEG = differentially expressed gene; FDR = false discovery rate; log2 = log base 2; -Log10 : negative log base 10; VEGFA = vascular endothelial growth factor A.

Figures 2a and 3a show the volcano plots of DEG in patients infected with *E. cloacae* and *S. aureus* respectively, compared to non-infected controls. (cf. Table S5 for *E. cloacae* and Table S6 for *S. aureus*). Some pathway differences were observed between the six patients infected with *S. aureus* and the two patients infected with *E. cloacae*. In the *E. cloacae* group (Fig. 2b), MHC class I molecules, which play an important role in cell-mediated immunity by reporting on intracellular events such as the presence of intracellular bacteria, were upregulated. In the *S. aureus* group platelet degranulation pathway and Ca^2+^ pathway were both downregulated pathways (Fig. 3b).

### 3.4 Difference in host response between bone and tissue biopsies

In the group of infected bone ( n=4 ) vs. non-infected control group ( n=16 ), we identified 32 upregulated genes and no downregulated genes with a p -adjusted < 0.05. *CXCL1, CXCL2, CCL4/L1/L2, CXCL3* were all significantly upregulated (Table S7 and Fig. S1 in the Supplement: volcano plot of infected bone versus non-infected control group). For infected tissue biopsies ( n=8 ) versus a non-infected control group ( n=16 ), the comparison showed nine upregulated genes and three downregulated genes (e.g. *CXCL14, CD36* and *CCL24*) with a p value of <0.05 (Table S8 and Fig. S2: volcano plot of infected tissue versus non-infected control group).

## 4 Discussion

In recent years, there has been growing interest in the field of osteoimmunology, to better understand the host response and its influence on the process of bone infection (Yoshii et al., 2002; Kamel Mohamed et al., 2005; Sabate-Bresco et al., 2021; Wilde et al., 2015; Sipprell et al., 2023). In this observational “proof-of-concept” study we performed transcriptome profiling on tissue and bone biopsy samples of patients undergoing revision surgery for suspected FRI. After applying the diagnostic criteria of the consensus definition and/or the decision of the multidisciplinary team, patients were divided in an infected group and a non-infected control group (McNally et al., 2020; Metsemakers et al., 2018). To our knowledge, this was the first study exploring the applicability of nCounter^®^ transcriptome profiling on detection of bacterial 16S rRNA and biofilm genes on tissue and bone biopsy samples.

The analysis of 16S rRNA demonstrated the power of transcriptomics in uncovering pathogens that are often missed by conventional diagnostics. Transcriptomics revealed additional pathogens not detected by culture, such as the co-infection of *E. cloacae* and *P. aeruginosa*. It also identified *S. aureus* in five cases missed by conventional methods with raw counts comparable to the infection group, potentially indicating cases of low-bacterial-load infections and therefore false-negative-culture results. However, four of these five cases were classified as non-infectious, had favourable outcomes, and did not require antibiotic treatment. Some culture-positive samples (e.g. *P. aeruginosa*, *E. cloacae*) did not show corresponding gene expression, suggesting either sample variability, possible contamination in our conventional culture method, or bacteria in dormant stage with low 16S rRNA expression. Bacterial gene expression as quantified by digital transcriptomics can be considered a reliable proxy for bacterial load and/or bacterial replication and metabolic activity, as was previously shown for several bacterial pathogens (Fukutani et al., 2018; Andrade et al., 2017). Moreover, compared to culture, 16S rRNA detection enables the identification of bacterial genetic material directly from tissue, including non-culturable or low-viability organisms, thereby potentially increasing diagnostic sensitivity.

For biofilm gene expression, we observed low signals, with the notable exception of an MRSA-positive biopsy with the highest 16S rRNA signal (cf. raw counts Table 3), which remains to be validated in future studies.

Pathway analysis in the infection group showed an upregulation of genes involved in the innate immune system, neutrophil degranulation, Toll-like receptor pathway, and MyD88 cascade. In implant-related infections, the implant acts as a foreign body that stimulates the innate immune system. Within the infected bone environment, pathogen-associated molecular patterns (PAMPs) activate TLRs on various cells, leading to the release of pro-inflammatory cytokines (e.g. *TNF, IL1, IL6*). These cytokines also promote osteoclast activation and differentiation, leading to bone resorption. Notably, while MyD88-dependent signalling is essential for infection control, it also contributes to pathological bone loss (Putnam et al., 2019), reflecting processes typical of biofilm-associated bone infections. Recent animal studies confirm that these pathways are critical in infection management yet contribute to chronic inflammation and bone degradation, underscoring the dual nature of the immune response in such infections (Seebach and Kubatzky, 2019; Putnam et al., 2019; Yoshimoto et al., 2022). Our enrichment analysis results in the infection group are consistent with the known processes regarding implant-related infections.

In the *S. aureus* infection group, there was decreased expression of the genes related to the “platelet degranulation” pathway and the “calcium pathway” associated with it (e.g. Ca^2+^ responsible for platelet degranulation). It is known that many bacteria directly activate the coagulation system or exhibit specific interactions with the vascular wall (Lemichez et al., 2010). Downregulation of this pathway could be an adaptive mechanism to evade immune response by reducing the release of antimicrobial peptides or limit clot formation to help spread the bacteria (Yeaman, 2010). *VEGFA*, a mediator of angiogenesis, is a notably upregulated gene in the *S. aureus* infection group. It was hypothesized that secretion of *VEGF* from infected macrophages may facilitate bacterial spread by inducing the formation of blood vessels (Maenetje et al., 2023; Polena et al., 2016).

Comparing the host response signal in the different specimens showed more differentially expressed genes in bone biopsies compared to tissue biopsies. Chemokines involved in the chemoattraction of monocytes/macrophages such as *CXCL1*, *CXCL2*, and *CCL4/L1/L2* were only upregulated in infected bone biopsies. Bone has a lower blood supply than soft tissue, making it harder for immune cells to reach the infection site. We hypothesized that the heightened chemokine response in bone biopsies could indicate that the body may “compensate” by producing more potent signals to attract immune cells, attempting to overcome these challenges (Kavanagh et al., 2018; Sipprell et al., 2023). However, future studies are necessary to elucidate this.

We have shown that prostaglandin-endoperoxide synthase 2 (*PTGS2*) is one of the most important upregulated genes in patients with FRI. *PTGS2* (or COX2) is the target for nonsteroidal anti-inflammatory drugs (NSAIDs). It was stated that NSAIDs might affect bone healing; however, data are controversial and based on animal models (Wildemann et al., 2021; George et al., 2020). It was recently shown that NSAIDs do not appear to impair human skeletogenic stem and progenitor cells (Goodnough et al., 2022). Caution is warranted in extrapolating data from animal models. Our results show that *PTGS2* is upregulated in the infection group. Whether this biomarker qualifies as a therapeutic target or diagnostic biomarker requires further exploration in clinical trials.

This study has a few limitations. Because of the proof-of-concept format, the sample size was relatively small. We need larger-cohort studies to gain more insight into the host response and into the host response according to sample origin and pathogen. In future studies, patient selection could also be more strategically focused on different infection types, ensuring sufficient sample sizes for each pathogen to allow more robust and meaningful comparisons. A second limitation is that no additional blood samples were collected for analysis of inflammatory markers in blood. A third limitation is the composition of the control group. The control group does not consist of “regular healing” patients because otherwise no surgery or (invasive) sample collection would have taken place for ethical reasons. This could possibly lead to a bias in our results. A fourth limitation is that the distinction between bone and tissue specimens was not absolute, as bone samples could sometimes contain adjacent soft tissue. This overlap could reduce the accuracy of direct comparisons of host response between the two sample types. All these limitations should be considered when analysis is performed on a larger prospective cohort.

## 5 Conclusion

In conclusion, this study advances our understanding of the immune response in FRI through the application of nCounter^®^ transcriptome profiling, also offering a promising approach for detecting 16S rRNA in tissue and bone biopsy samples.

Transcriptomic analysis uncovered additional infections not detected by conventional culture methods, highlighting its superior sensitivity, particularly in cases where low bacterial loads or sample heterogeneity may probably hinder detection. This underscores the limitations of culture methods and supports an added value of transcriptomic techniques for capturing bacteria in complex infections. Although transcriptomics shows higher sensitivity, its clinical applicability depends on balancing sensitivity with specificity, particularly in differentiating true infection from background presence.

Furthermore, pathway analysis in the infection group showed an upregulation of innate immune signalling pathways, TLR, and MyD88 cascades, which are crucial for infection control yet also contribute to pathological bone loss. Our findings indicate that immune responses in infected bone biopsies are enhanced compared to tissue biopsies.

Finally, the significant upregulation of *PTGS2* (COX2) in FRI patients suggests potential as a therapeutic target or diagnostic biomarker, although further clinical trials are needed to determine its suitability in humans, given the varied data from animal models.

Larger cohorts could provide deeper insights into the immune response and our understanding of genomic elements that contribute to disease processes, possibly leading to the discovery of new diagnostic biomarkers and the development of adjuvant treatments targeting key immunomodulatory pathways.

## Supplement

10.5194/jbji-11-161-2026-supplement The supplement related to this article is available online at https://doi.org/10.5194/jbji-11-161-2026-supplement.

## Data Availability

All data supporting this study are included in this article (Tables 2, 3; Figs. 1, 2 and 3) and in the Supplement. nCounter^®^ gene expression data are available in the Supplement.
