# Supplementary material for: Combined analysis of host immune response, biofilm genes, and 16S rRNA detection in fracture-related infection: an observational cohort study
Source: J Bone Jt Infect. 2026 Mar 12;11(2):161–73. doi: 10.5194/jbji-11-161-2026 (PMC13044966; doi:10.5194/jbji-11-161-2026)

Supplement of J. Bone Joint Infect., 11, 161–173, 2026
https://doi.org/10.5194/jbji-11-161-2026-supplement
© Author(s) 2026. CC BY 4.0 License.

Supplement of

Combined analysis of host immune response, biofilm genes, and 16S rRNA detection in fracture-related infection: an observational cohort study

Melissa Depypere et al.

Correspondence to: Melissa Depypere (melissa.depypere@uzleuven.be)

The copyright of individual parts of the supplement might differ from the article licence.

Table S1: Overview of Target Genes included in the Transcriptomic Panel

This table summarizes all genes included in the NanoString transcriptomic panel (nCounter Human Host Response Panel, catalogue number 531-115000449). Gene symbols follow HUGO nomenclature. The 12 internal reference genes used for normalization are: *ACTB, B2M, GAPDH, HPRT1, RPL13A, TBP, PPIA, GUSB, PGK1, SDHA, YWHAZ,* and *RPLP0*. Each gene was categorized according to five broader biological categories: adaptive immune response, homeostasis, host susceptibility, innate immune cell activation, and interferon response.

**Gene symbol**	**Full name**	**Adaptive immune response**	**Homesostasis**	**Host susceptibility**	**Innate immune cell activation**	**Interferon response**
ACE	angiotensin I converting enzyme	-	+	+	-	-
ACKR2	atypical chemokine receptor 2	-	-	-	+	-
ACKR3	atypical chemokine receptor 3	-	-	-	+	-
ACKR4	atypical chemokine receptor 4	-	-	-	+	-
ACOX1	acyl-CoA oxidase 1	-	-	-	+	-
ACSL1	acyl-CoA synthetase long chain family member 1	-	-	-	+	-
ACSL3	acyl-CoA synthetase long chain family member 3	-	-	-	+	-
ACSL4	acyl-CoA synthetase long chain family member 4	-	-	-	+	-
ACVR1	activin A receptor type 1	+	+	-	+	-
ADAR	adenosine deaminase RNA specific	-	-	-	-	+
ADGRE5	adhesion G protein-coupled receptor E5	-	-	-	+	-
ADGRG3	adhesion G protein-coupled receptor G3	-	-	-	+	-
ADORA2A	adenosine A2a receptor	+	-	-	+	-
AGT	angiotensinogen	-	+	+	+	-
AHR	aryl hydrocarbon receptor	+	-	-	-	-
AIF1	allograft inflammatory factor 1	+	+	-	+	-
AIM2	absent in melanoma 2	-	-	-	-	+
AKT1	AKT serine/threonine kinase 1	+	+	-	+	-

AKT2	AKT serine/threonine kinase 2	+	+	-	+	-
AKT3	AKT serine/threonine kinase 3	+	+	-	+	-
ALOX12	arachidonate 12-lipoxygenase, 12S type	-	+	-	-	-
ALOX15	arachidonate 15-lipoxygenase	+	+	-	+	-
ALOX5	arachidonate 5-lipoxygenase	+	+	-	+	-
ALOX5AP	arachidonate 5-lipoxygenase activating protein	-	+	-	+	-
ALPK1	alpha kinase 1	-	-	-	-	+
ALPL	alkaline phosphatase, biomineralization associated	-	-	-	+	-
ANPEP	alanyl aminopeptidase, membrane	-	+	+	+	-
AP1G1	adaptor related protein complex 1 subunit gamma 1	+	+	-	-	-
AP1M1	adaptor related protein complex 1 subunit mu 1	+	+	-	+	-
AP1S2	adaptor related protein complex 1 subunit sigma 2	+	+	-	-	-
APBB1IP	amyloid beta precursor protein binding family B member 1 interacting protein	-	+	-	-	-
APEX1	apurinic/apyrimidinic endodeoxyribonuclease 1	-	+	-	+	-
APOBEC3G	apolipoprotein B mRNA editing enzyme catalytic subunit 3G	-	-	-	-	+
APOL6	apolipoprotein L6	-	-	-	-	+
APP	amyloid beta precursor protein	+	-	-	+	+
ARRB2	arrestin beta 2	-	+	-	+	-
ATF2	activating transcription factor 2	+	+	-	+	+
ATF4	activating transcription factor 4	-	+	-	+	-
ATF6	activating transcription factor 6	-	+	-	-	-
ATG10	autophagy related 10	-	+	-	-	-
ATG12	autophagy related 12	-	+	-	-	+

ATG13	autophagy related 13	-	+	-	-	-
ATG3	autophagy related 3	-	+	-	-	-
ATG4A	autophagy related 4A cysteine peptidase	-	+	-	-	-
ATG7	autophagy related 7	+	+	-	+	-
ATM	ATM serine/threonine kinase	+	+	-	+	-
ATP6AP2	ATPase H+ transporting accessory protein 2	-	+	+	+	-
ATP6V0D1	ATPase H+ transporting V0 subunit d1	-	+	-	+	-
ATP6V1B2	ATPase H+ transporting V1 subunit B2	-	-	-	+	-
BATF	basic leucine zipper ATF-like transcription factor	+	-	-	+	-
BCL2	BCL2 apoptosis regulator	+	+	-	+	+
BCL2L1	BCL2 like 1	+	+	-	+	+
BCL3	BCL3 transcription coactivator	-	+	-	+	-
BCL6	BCL6 transcription repressor	+	-	-	+	-
BCR	BCR activator of RhoGEF and GTPase	-	-	-	+	-
BDKRB1	bradykinin receptor B1	+	+	+	-	-
BDKRB2	bradykinin receptor B2	+	+	+	-	-
BECN1	beclin 1	-	+	-	-	-
BLK	BLK proto-oncogene, Src family tyrosine kinase	+	-	-	-	-
BNIP3	BCL2 interacting protein 3	-	+	-	+	-
BPI	bactericidal permeability increasing protein	-	-	-	+	+
BST2	bone marrow stromal cell antigen 2	+	+	-	+	+
C1QBP	complement C1q binding protein	-	+	-	-	-
C2	complement C2	+	-	-	-	-
C3	complement C3	+	-	-	+	-
C3AR1	complement C3a receptor 1	+	-	-	+	-
C5	complement C5	+	-	-	-	-

C5AR1	complement C5a receptor 1	+	-	-	+	-
CALM1	calmodulin 1	+	+	-	+	+
CAP1	cyclase associated actin cytoskeleton regulatory protein 1	-	+	-	+	-
CARD11	caspase recruitment domain family member 11	+	-	-	+	+
CARD16	caspase recruitment domain family member 16	-	-	-	-	+
CARD17	caspase recruitment domain family member 17	-	-	-	-	+
CASP1	caspase 1	+	-	-	+	+
CASP10	caspase 10	-	+	-	+	+
CASP3	caspase 3	+	+	-	+	-
CASP4	caspase 4	-	-	-	-	+
CASP5	caspase 5	-	-	-	-	+
CASP8	caspase 8	-	+	-	+	+
CBFB	core-binding factor subunit beta	-	+	-	-	-
CBL	Cbl proto-oncogene	+	+	-	+	-
CBLB	Cbl proto-oncogene B	+	-	-	-	-
CCL1	C-C motif chemokine ligand 1	+	-	-	+	-
CCL11	C-C motif chemokine ligand 11	+	-	-	+	-
CCL13	C-C motif chemokine ligand 13	+	-	-	+	-
CCL14	C-C motif chemokine ligand 14	+	-	-	+	-
CCL15	C-C motif chemokine ligand 15	+	-	-	+	-
CCL16	C-C motif chemokine ligand 16	+	-	-	+	-
CCL17	C-C motif chemokine ligand 17	+	-	-	+	-
CCL18	C-C motif chemokine ligand 18	+	-	-	+	-
CCL19	C-C motif chemokine ligand 19	+	-	-	+	-
CCL2	C-C motif chemokine ligand 2	+	+	-	+	+
CCL20	C-C motif chemokine ligand 20	+	+	-	+	-

CCL21	C-C motif chemokine ligand 21	+	-	-	+	-
CCL22	C-C motif chemokine ligand 22	+	-	-	+	-
CCL23	C-C motif chemokine ligand 23	+	-	-	+	-
CCL24	C-C motif chemokine ligand 24	+	-	-	+	-
CCL25	C-C motif chemokine ligand 25	+	-	-	+	-
CCL26	C-C motif chemokine ligand 26	+	-	-	+	-
CCL27	C-C motif chemokine ligand 27	+	-	-	+	-
CCL28	C-C motif chemokine ligand 28	-	-	-	+	-
CCL3/L1/L3	C-C motif chemokine ligand 3	+	-	-	+	-
CCL4/L1/L2	C-C motif chemokine ligand 4	+	-	-	+	+
CCL5	C-C motif chemokine ligand 5	+	+	-	+	+
CCL7	C-C motif chemokine ligand 7	+	-	-	+	-
CCL8	C-C motif chemokine ligand 8	+	-	-	+	-
CCNC	cyclin C	+	+	-	+	-
CCR1	C-C motif chemokine receptor 1	+	-	-	+	-
CCR10	C-C motif chemokine receptor 10	-	-	-	+	-
CCR2	C-C motif chemokine receptor 2	+	-	-	+	-
CCR3	C-C motif chemokine receptor 3	-	-	-	+	-
CCR4	C-C motif chemokine receptor 4	-	-	-	+	-
CCR5	C-C motif chemokine receptor 5	-	-	+	+	-
CCR6	C-C motif chemokine receptor 6	-	-	-	+	-
CCR7	C-C motif chemokine receptor 7	+	+	-	+	-
CCR8	C-C motif chemokine receptor 8	-	-	-	+	-
CCR9	C-C motif chemokine receptor 9	-	-	-	+	-
CCRL2	C-C motif chemokine receptor like 2	-	-	-	+	-
CD14	CD14 molecule	+	-	-	+	+
CD163	CD163 molecule	-	-	-	+	-
CD19	CD19 molecule	+	-	+	-	-
CD1E	CD1e molecule	+	-	-	-	-

CD2	CD2 molecule	+	-	-	+	-
CD209	CD209 molecule	-	-	+	+	+
CD22	CD22 molecule	+	-	-	-	-
CD244	CD244 molecule	+	-	-	+	-
CD247	CD247 molecule	+	-	-	+	-
CD27	CD27 molecule	+	-	-	+	-
CD274	CD274 molecule	+	-	-	-	-
CD276	CD276 molecule	+	-	-	-	-
CD28	CD28 molecule	+	-	-	-	-
CD36	CD36 molecule	+	+	-	+	+
CD38	CD38 molecule	-	+	-	+	-
CD3D	CD3d molecule	+	-	-	-	-
CD3E	CD3e molecule	+	-	-	-	-
CD3G	CD3g molecule	+	-	-	+	-
CD4	CD4 molecule	+	-	+	+	-
CD40	CD40 molecule	+	-	-	+	-
CD40LG	CD40 ligand	+	-	-	+	-
CD44	CD44 molecule (Indian blood group)	-	-	-	+	+
CD45R0	protein tyrosine phosphatase receptor type C	+	-	-	+	-
CD45RA	protein tyrosine phosphatase receptor type C	+	-	-	+	-
CD45RB	protein tyrosine phosphatase receptor type C	+	-	-	+	-
CD59	CD59 molecule (CD59 blood group)	+	-	-	+	-
CD6	CD6 molecule	+	-	-	-	-
CD68	CD68 molecule	-	+	-	+	-
CD69	CD69 molecule	+	-	-	-	-
CD70	CD70 molecule	+	-	-	+	-
CD79A	CD79a molecule	+	-	-	-	-

CD79B	CD79b molecule	+	-	-	-	-
CD80	CD80 molecule	+	-	-	+	-
CD81	CD81 molecule	+	-	+	-	-
CD84	CD84 molecule	-	-	-	+	-
CD86	CD86 molecule	+	-	-	+	-
CD8A	CD8a molecule	+	-	-	-	-
CD8B	CD8b molecule	+	-	-	-	-
CDH1	cadherin 1	+	+	-	-	-
CDK4	cyclin dependent kinase 4	+	-	-	-	-
CEACAM3	CEA cell adhesion molecule 3	-	-	-	+	-
CEBPB	CCAAT enhancer binding protein beta	-	+	-	+	-
CFLAR	CASP8 and FADD like apoptosis regulator	+	+	-	+	-
CGAS	cyclic GMP-AMP synthase	-	-	-	-	+
CHUK	component of inhibitor of nuclear factor kappa B kinase complex	+	+	-	+	+
CPA3	carboxypeptidase A3	-	+	+	-	-
CR1	complement C3b/C4b receptor 1 (Knops blood group)	+	-	-	+	-
CREBBP	CREB binding protein	+	+	-	+	+
CRK	CRK proto-oncogene, adaptor protein	-	+	-	+	-
CRP	C-reactive protein	+	+	-	-	-
CSF1	colony stimulating factor 1	+	+	-	+	-
CSF1R	colony stimulating factor 1 receptor	+	-	-	+	-
CSF2	colony stimulating factor 2	+	+	-	+	+
CSF2RA	colony stimulating factor 2 receptor subunit alpha	+	-	-	+	+
CSF2RB	colony stimulating factor 2 receptor subunit beta	+	-	-	+	+
CSF3	colony stimulating factor 3	+	-	-	+	+

CSF3R	colony stimulating factor 3 receptor	+	-	-	+	+
CTLA4	cytotoxic T-lymphocyte associated protein 4	+	-	-	-	-
CTSA	cathepsin A	+	+	+	+	-
CTSG	cathepsin G	+	+	+	+	-
CTSL	cathepsin L	+	+	-	-	+
CTSS	cathepsin S	+	+	-	+	+
CTSW	cathepsin W	-	+	-	-	-
CTSZ	cathepsin Z	-	+	-	+	-
CUL1	cullin 1	+	+	-	+	+
CX3CL1	C-X3-C motif chemokine ligand 1	+	+	-	+	-
CX3CR1	C-X3-C motif chemokine receptor 1	-	-	-	+	-
CXCL1	C-X-C motif chemokine ligand 1	+	+	-	+	+
CXCL10	C-X-C motif chemokine ligand 10	+	+	-	+	+
CXCL11	C-X-C motif chemokine ligand 11	-	-	-	+	-
CXCL12	C-X-C motif chemokine ligand 12	+	-	-	+	-
CXCL13	C-X-C motif chemokine ligand 13	-	-	-	+	-
CXCL14	C-X-C motif chemokine ligand 14	-	-	-	+	-
CXCL16	C-X-C motif chemokine ligand 16	-	-	-	+	-
CXCL17	C-X-C motif chemokine ligand 17	+	-	-	+	-
CXCL2	C-X-C motif chemokine ligand 2	+	+	-	+	+
CXCL3	C-X-C motif chemokine ligand 3	+	+	-	+	+
CXCL5	C-X-C motif chemokine ligand 5	-	+	-	+	-
CXCL6	C-X-C motif chemokine ligand 6	-	+	-	+	-
CXCL8	C-X-C motif chemokine ligand 8	+	+	-	+	+
CXCL9	C-X-C motif chemokine ligand 9	-	-	-	+	-
CXCR1	C-X-C motif chemokine receptor 1	-	-	-	+	-
CXCR2	C-X-C motif chemokine receptor 2	-	-	-	+	-
CXCR3	C-X-C motif chemokine receptor 3	-	-	-	+	-

CXCR4	C-X-C motif chemokine receptor 4	+	-	+	+	-
CXCR5	C-X-C motif chemokine receptor 5	-	-	-	+	-
CXCR6	C-X-C motif chemokine receptor 6	-	-	-	+	-
CYP2E1	cytochrome P450 family 2 subfamily E member 1	-	+	-	+	-
CYSTM1	cysteine rich transmembrane module containing 1	-	-	-	+	-
DDAH2	dimethylarginine dimethylaminohydrolase 2	-	+	-	+	-
DDIT3	DNA damage inducible transcript 3	+	+	-	+	+
DDOST	dolichyl-diphosphooligosaccharide--protein glycosyltransferase non-catalytic subunit	-	+	-	+	-
DDX5	DEAD-box helicase 5	-	-	+	-	-
DDX58	DExD/H-box helicase 58	+	-	-	+	+
DEFA4	defensin alpha 4	-	-	-	+	+
DEFB103A/B	defensin beta 103A/B	-	-	-	+	+
DERL1	derlin 1	-	+	-	-	-
DHX58	DExH-box helicase 58	-	-	-	-	+
DIABLO	diablo IAP-binding mitochondrial protein	-	+	-	+	-
DNAJA2	DnaJ heat shock protein family (Hsp40) member A2	-	+	-	-	-
DNAJC10	DnaJ heat shock protein family (Hsp40) member C10	-	+	-	-	-
DTX3L	deltex E3 ubiquitin ligase 3L	+	-	-	-	-
DYSF	dysferlin	-	-	-	+	-
EBI3	Epstein-Barr virus induced 3	+	-	-	+	-
EGLN1	egl-9 family hypoxia inducible factor 1	-	+	-	+	-
EIF2AK2	eukaryotic translation initiation factor 2 alpha kinase 2	-	+	-	-	+

EIF2AK3	eukaryotic translation initiation factor 2 alpha kinase 3	-	+	-	-	-
EIF3F	eukaryotic translation initiation factor 3 subunit F	-	-	+	-	-
ELANE	elastase, neutrophil expressed	+	-	-	+	-
ENTPD1	ectonucleoside triphosphate diphosphohydrolase 1	-	-	-	+	-
EOMES	eomesodermin	+	-	-	-	-
EPHX2	epoxide hydrolase 2	-	+	-	-	-
ERN1	endoplasmic reticulum to nucleus signaling 1	-	+	-	-	-
ETS1	ETS proto-oncogene 1, transcription factor	-	+	-	+	-
EVL	Enah/Vasp-like	+	-	-	-	-
F5	coagulation factor V	+	+	-	-	-
FAM30A	family with sequence similarity 30 member A	-	-	-	-	-
FAS	Fas cell surface death receptor	+	+	-	+	-
FASLG	Fas ligand	+	+	-	+	-
FBXO6	F-box protein 6	+	+	-	-	-
FCAR	Fc fragment of IgA receptor	-	+	-	+	-
FCGR1A/B	Fc fragment of IgG receptor Ia/b	+	-	-	+	+
FCGR2A	Fc fragment of IgG receptor IIa	-	-	-	+	-
FCGR3A/B	Fc fragment of IgG receptor IIIa/b	-	-	-	+	-
FCGRT	Fc fragment of IgG receptor and transporter	-	-	-	+	-
FCRL2	Fc receptor like 2	-	-	-	-	-
FCRL4	Fc receptor like 4	+	-	-	-	-
FGR	FGR proto-oncogene, Src family tyrosine kinase	-	-	-	+	-
FOS	Fos proto-oncogene, AP-1 transcription factor subunit	+	+	-	+	+

FOXO1	forkhead box O1	+	+	-	+	-
FOXP3	forkhead box P3	+	-	-	-	-
FPR1	formyl peptide receptor 1	+	+	-	+	-
FPR2	formyl peptide receptor 2	+	-	-	+	-
FURIN	furin, paired basic amino acid cleaving enzyme	+	+	+	+	-
FYN	FYN proto-oncogene, Src family tyrosine kinase	+	-	-	+	+
GAB2	GRB2 associated binding protein 2	+	-	-	+	-
GADD45B	growth arrest and DNA damage inducible beta	+	+	-	+	-
GATA3	GATA binding protein 3	+	-	-	+	-
GBA	glucosylceramidase beta	-	+	-	+	-
GBP1	guanylate binding protein 1	-	-	-	-	+
GBP2	guanylate binding protein 2	-	-	-	-	+
GBP4	guanylate binding protein 4	-	-	-	-	+
GBP5	guanylate binding protein 5	-	-	-	-	+
GCA	grancalcin	-	-	-	+	-
GK	glycerol kinase	-	-	-	+	-
GLA	galactosidase alpha	-	+	-	+	-
GLB1	galactosidase beta 1	-	+	-	+	-
GNLY	granulysin	-	-	-	+	-
GNS	glucosamine (N-acetyl)-6-sulfatase	-	+	-	+	-
GPX7	glutathione peroxidase 7	-	+	-	+	-
GSK3B	glycogen synthase kinase 3 beta	+	-	-	-	-
GSTM4	glutathione S-transferase mu 4	-	+	-	-	-
GUCY1A1	guanylate cyclase 1 soluble subunit alpha 1	-	+	-	+	-
GUCY1B1	guanylate cyclase 1 soluble subunit beta 1	-	+	-	+	-

GZMA	granzyme A	+	-	-	+	-
GZMB	granzyme B	+	+	-	+	-
GZMH	granzyme H	+	-	-	+	-
HAMP	hepcidin antimicrobial peptide	+	+	-	+	-
HAVCR2	hepatitis A virus cellular receptor 2	+	-	-	+	-
HCK	HCK proto-oncogene, Src family tyrosine kinase	-	-	-	+	-
HCST	hematopoietic cell signal transducer	-	-	-	+	-
HDC	histidine decarboxylase	-	-	-	+	-
HERC5	HECT and RLD domain containing E3 ubiquitin protein ligase 5	+	-	-	-	+
HK3	hexokinase 3	-	+	-	+	-
HLA-A	major histocompatibility complex, class I, A	+	-	-	-	+
HLA-B	major histocompatibility complex, class I, B	+	-	-	+	+
HLA-C	major histocompatibility complex, class I, C	+	-	-	+	+
HLA-DMA	major histocompatibility complex, class II, DM alpha	+	-	-	-	-
HLA-DMB	major histocompatibility complex, class II, DM beta	+	-	-	-	-
HLA-DOB	major histocompatibility complex, class II, DO beta	+	-	-	-	-
HLA-DPA1	major histocompatibility complex, class II, DP alpha 1	+	-	-	-	+
HLA-DPB1	major histocompatibility complex, class II, DP beta 1	+	-	-	-	+
HLA-DQA	major histocompatibility complex, class II, DQ alpha	+	-	-	+	+
HLA-DQB1	major histocompatibility complex, class II, DQ beta 1	+	-	-	-	+

HLA-DRA	major histocompatibility complex, class II, DR alpha	+	-	-	-	+
HLA-DRB	CD74 molecule	+	-	-	+	+
HLA-E	major histocompatibility complex, class I, E	+	-	-	-	+
HLX	H2.0 like homeobox	+	-	-	-	-
HMGB1	high mobility group box 1	+	-	-	+	+
HMOX1	heme oxygenase 1	+	+	-	+	-
HPGD	15-hydroxyprostaglandin dehydrogenase	-	+	-	-	-
HSD11B1	hydroxysteroid 11-beta dehydrogenase 1	-	-	-	-	-
HSP90AA1	heat shock protein 90 alpha family class A member 1	+	+	-	+	+
HSP90AB1	heat shock protein 90 alpha family class B member 1	+	+	-	+	+
HSP90B1	heat shock protein 90 beta family member 1	+	+	-	+	+
ICAM3	intercellular adhesion molecule 3	+	-	-	-	+
ICOS	inducible T cell costimulator	+	-	-	-	-
ICOSLG	inducible T cell costimulator ligand	+	-	-	-	-
IDO1	indoleamine 2,3-dioxygenase 1	-	-	-	+	-
IFI16	interferon gamma inducible protein 16	-	-	-	-	+
IFI27	interferon alpha inducible protein 27	+	+	-	+	+
IFI35	interferon induced protein 35	-	-	-	-	+
IFI44	interferon induced protein 44	-	-	-	-	+
IFI6	interferon alpha inducible protein 6	-	-	-	-	+
IFIH1	interferon induced with helicase C domain 1	-	-	-	-	+
IFIT1	interferon induced protein with tetratricopeptide repeats 1	-	-	-	-	+
IFIT2	interferon induced protein with tetratricopeptide repeats 2	-	-	-	-	+

IFIT3	interferon induced protein with tetratricopeptide repeats 3	-	-	-	-	+
IFITM1	interferon induced transmembrane protein 1	+	+	-	+	+
IFITM2	interferon induced transmembrane protein 2	+	+	-	+	+
IFITM3	interferon induced transmembrane protein 3	+	+	-	+	+
IFNA1/13	interferon alpha 1/13	+	-	-	+	+
IFNA14/16	interferon alpha 14/16	+	-	-	+	+
IFNA2	interferon alpha 2	+	-	-	+	+
IFNA4/7/10/17/21	interferon alpha 4/7/10/17/21	+	-	-	+	+
IFNA5	interferon alpha 5	+	-	-	+	+
IFNA6	interferon alpha 6	+	-	-	+	+
IFNA8	interferon alpha 8	+	-	-	+	+
IFNAR1	interferon alpha and beta receptor subunit 1	+	-	-	+	+
IFNAR2	interferon alpha and beta receptor subunit 2	+	-	-	+	+
IFNB1	interferon beta 1	+	+	-	+	+
IFNG	interferon gamma	+	+	-	+	+
IFNGR2	interferon gamma receptor 2	+	+	-	+	+
IFNK	interferon kappa	+	-	-	+	+
IFNL1	interferon lambda 1	+	-	-	+	+
IFNL2/3	interferon lambda 2/3	+	-	-	+	+
IFNL4	interferon lambda 4 (gene/pseudogene)	-	-	-	-	+
IFNLR1	interferon lambda receptor 1	+	-	-	+	+
IFNW1	interferon omega 1	+	-	-	+	+
IGFBP7	insulin like growth factor binding protein 7	-	+	-	-	-

IKBKB	inhibitor of nuclear factor kappa B kinase subunit beta	+	+	-	+	+
IKBKE	inhibitor of nuclear factor kappa B kinase subunit epsilon	-	-	-	-	+
IKBKG	inhibitor of nuclear factor kappa B kinase regulatory subunit gamma	+	+	-	+	+
IL10	interleukin 10	+	-	-	+	+
IL10RA	interleukin 10 receptor subunit alpha	+	-	-	+	+
IL10RB	interleukin 10 receptor subunit beta	+	-	-	+	+
IL11	interleukin 11	+	-	-	+	+
IL11RA	interleukin 11 receptor subunit alpha	+	-	-	+	+
IL12A	interleukin 12A	+	-	-	+	+
IL12B	interleukin 12B	+	-	-	+	+
IL12RB1	interleukin 12 receptor subunit beta 1	+	-	-	+	+
IL12RB2	interleukin 12 receptor subunit beta 2	+	-	-	+	+
IL13	interleukin 13	+	-	-	+	+
IL13RA1	interleukin 13 receptor subunit alpha 1	+	-	-	+	+
IL13RA2	interleukin 13 receptor subunit alpha 2	+	-	-	+	+
IL15	interleukin 15	+	+	-	+	+
IL15RA	interleukin 15 receptor subunit alpha	+	-	-	+	+
IL16	interleukin 16	+	-	-	+	-
IL17A	interleukin 17A	+	-	-	+	-
IL17B	interleukin 17B	+	-	-	+	-
IL17C	interleukin 17C	+	-	-	+	-
IL17D	interleukin 17D	+	-	-	+	+
IL17F	interleukin 17F	+	-	-	+	-
IL17RA	interleukin 17 receptor A	+	-	-	+	-
IL17RB	interleukin 17 receptor B	+	-	-	+	-
IL17RC	interleukin 17 receptor C	+	-	-	+	-
IL17RD	interleukin 17 receptor D	+	-	-	+	-

IL17RE	interleukin 17 receptor E	+	-	-	+	-
IL18	interleukin 18	+	-	-	+	+
IL18BP	interleukin 18 binding protein	+	+	-	+	-
IL18R1	interleukin 18 receptor 1	+	+	-	+	-
IL18RAP	interleukin 18 receptor accessory protein	+	+	-	+	-
IL19	interleukin 19	+	-	-	+	+
IL1A	interleukin 1 alpha	+	-	-	+	-
IL1B	interleukin 1 beta	+	+	-	+	+
IL1F10	interleukin 1 family member 10	+	-	-	+	-
IL1R1	interleukin 1 receptor type 1	+	-	-	+	-
IL1R2	interleukin 1 receptor type 2	+	-	-	+	-
IL1RAP	interleukin 1 receptor accessory protein	+	-	-	-	-
IL1RAPL1	interleukin 1 receptor accessory protein like 1	+	-	-	+	-
IL1RAPL2	interleukin 1 receptor accessory protein like 2	+	-	-	+	-
IL1RL1	interleukin 1 receptor like 1	+	-	-	+	-
IL1RL2	interleukin 1 receptor like 2	+	-	-	+	-
IL1RN	interleukin 1 receptor antagonist	+	-	-	+	-
IL2	interleukin 2	+	-	-	+	+
IL20	interleukin 20	+	-	-	+	+
IL20RA	interleukin 20 receptor subunit alpha	+	-	-	+	+
IL20RB	interleukin 20 receptor subunit beta	+	-	-	+	+
IL21	interleukin 21	+	-	-	+	+
IL21R	interleukin 21 receptor	+	-	-	+	+
IL22	interleukin 22	+	-	-	+	+
IL22RA1	interleukin 22 receptor subunit alpha 1	+	-	-	+	+
IL22RA2	interleukin 22 receptor subunit alpha 2	+	-	-	+	+
IL23A	interleukin 23 subunit alpha	+	-	-	+	+

IL23R	interleukin 23 receptor	+	-	-	+	+
IL24	interleukin 24	+	-	-	+	+
IL25	interleukin 25	+	-	-	+	-
IL26	interleukin 26	+	-	-	+	-
IL27	interleukin 27	+	-	-	+	-
IL27RA	interleukin 27 receptor subunit alpha	+	-	-	+	+
IL2RA	interleukin 2 receptor subunit alpha	+	-	-	+	+
IL2RB	interleukin 2 receptor subunit beta	+	-	-	+	+
IL2RG	interleukin 2 receptor subunit gamma	+	-	-	+	+
IL3	interleukin 3	+	-	-	+	+
IL31	interleukin 31	+	-	-	+	-
IL31RA	interleukin 31 receptor A	+	-	-	+	-
IL32	interleukin 32	+	-	-	+	-
IL33	interleukin 33	+	-	-	+	+
IL34	interleukin 34	+	-	-	+	-
IL36A	interleukin 36 alpha	+	-	-	+	-
IL36B	interleukin 36 beta	+	-	-	+	-
IL36G	interleukin 36 gamma	+	-	-	+	-
IL36RN	interleukin 36 receptor antagonist	+	-	-	+	-
IL37	interleukin 37	+	-	-	+	-
IL3RA	interleukin 3 receptor subunit alpha	+	-	-	+	+
IL4	interleukin 4	+	-	-	+	+
IL4R	interleukin 4 receptor	+	-	-	+	+
IL5	interleukin 5	+	-	-	+	+
IL5RA	interleukin 5 receptor subunit alpha	+	-	-	+	+
IL6	interleukin 6	+	+	-	+	+
IL6R	interleukin 6 receptor	+	+	-	+	+
IL6ST	interleukin 6 signal transducer	+	-	-	+	+
IL7	interleukin 7	+	-	-	+	+

IL7R	interleukin 7 receptor	+	-	-	+	+
IL9	interleukin 9	+	-	-	+	+
IL9R	interleukin 9 receptor	+	-	-	+	+
IRAK1	interleukin 1 receptor associated kinase 1	+	-	-	+	+
IRAK3	interleukin 1 receptor associated kinase 3	+	-	-	+	+
IRAK4	interleukin 1 receptor associated kinase 4	+	-	-	+	+
IRF1	interferon regulatory factor 1	-	+	-	+	+
IRF3	interferon regulatory factor 3	-	-	-	-	+
IRF4	interferon regulatory factor 4	+	-	-	+	+
IRF7	interferon regulatory factor 7	-	-	-	-	+
IRF9	interferon regulatory factor 9	-	-	-	-	+
ISG15	ISG15 ubiquitin like modifier	-	-	-	-	+
ITGAE	integrin subunit alpha E	+	-	-	-	-
ITGAL	integrin subunit alpha L	+	-	-	+	-
ITGAM	integrin subunit alpha M	+	-	-	+	+
ITGAX	integrin subunit alpha X	+	-	-	+	-
ITGB2	integrin subunit beta 2	+	-	-	+	+
ITGB7	integrin subunit beta 7	+	-	-	-	-
ITK	IL2 inducible T cell kinase	+	-	-	+	-
ITLN1	intelectin 1	-	-	-	+	-
ITPR3	inositol 1,4,5-trisphosphate receptor type 3	+	-	-	+	+
JAK1	Janus kinase 1	+	-	-	+	+
JAK2	Janus kinase 2	+	-	-	+	+
JAK3	Janus kinase 3	+	-	-	+	-
JAML	junction adhesion molecule like	+	-	-	+	-

JUN	Jun proto-oncogene, AP-1 transcription factor subunit	+	+	-	+	+
JUNB	JunB proto-oncogene, AP-1 transcription factor subunit	+	+	-	+	+
KDM6B	lysine demethylase 6B	-	+	-	+	-
KIR2DL1	killer cell immunoglobulin like receptor, two Ig domains and long cytoplasmic tail 1	-	-	-	+	-
KIR2DL3	killer cell immunoglobulin like receptor, two Ig domains and long cytoplasmic tail 3	-	-	-	+	-
KIR3DL1/2	killer cell immunoglobulin like receptor, three Ig domains and long cytoplasmic tail 1/2	-	-	-	+	-
KLRB1	killer cell lectin like receptor B1	-	-	-	+	-
KLRC1	killer cell lectin like receptor C1	-	-	-	+	-
KLRD1	killer cell lectin like receptor D1	-	-	-	+	-
KLRK1	killer cell lectin like receptor K1	-	-	-	+	-
KPNB1	karyopherin subunit beta 1	-	-	-	+	+
KRAS	KRAS proto-oncogene, GTPase	+	-	-	-	-
LAG3	lymphocyte activating 3	+	-	-	-	-
LAMP1	lysosomal associated membrane protein 1	-	+	-	+	-
LAMP2	lysosomal associated membrane protein 2	-	+	-	+	-
LAMP3	lysosomal associated membrane protein 3	-	+	-	-	-
LANCL1	LanC like 1	-	+	-	+	-
LAT	linker for activation of T cells	+	-	-	+	-
LAT2	linker for activation of T cells family member 2	-	-	-	+	-
LCK	LCK proto-oncogene, Src family tyrosine kinase	+	-	-	+	-

LCN2	lipocalin 2	+	-	-	+	-
LCP1	lymphocyte cytosolic protein 1	+	-	-	+	-
LCP2	lymphocyte cytosolic protein 2	+	-	-	+	-
LDHB	lactate dehydrogenase B	-	+	-	-	-
LEF1	lymphoid enhancer binding factor 1	+	-	-	-	-
LGALS3	galectin 3	+	-	-	+	-
LIF	LIF interleukin 6 family cytokine	+	+	-	+	+
LILRA3	leukocyte immunoglobulin like receptor A3	+	-	-	+	-
LILRA5	leukocyte immunoglobulin like receptor A5	+	-	-	-	-
LILRA6	leukocyte immunoglobulin like receptor A6	+	-	-	-	-
LILRB2	leukocyte immunoglobulin like receptor B2	+	-	-	+	-
LIMK2	LIM domain kinase 2	-	-	-	+	-
LITAF	lipopolysaccharide induced TNF factor	-	+	-	-	-
LRG1	leucine rich alpha-2-glycoprotein 1	-	-	-	+	-
LRRK2	leucine rich repeat kinase 2	-	+	-	+	-
LTA4H	leukotriene A4 hydrolase	-	+	-	+	-
LTB	lymphotoxin beta	+	-	-	+	-
LTBR	lymphotoxin beta receptor	+	+	-	+	-
LTC4S	leukotriene C4 synthase	-	+	-	-	-
LTF	lactotransferrin	-	-	-	+	-
LYN	LYN proto-oncogene, Src family tyrosine kinase	+	-	-	+	+
MAF	MAF bZIP transcription factor	+	-	-	-	-
MAFB	MAF bZIP transcription factor B	-	-	-	+	-
MAP1LC3A	microtubule associated protein 1 light chain 3 alpha	-	+	-	+	-

MAP2K2	mitogen-activated protein kinase kinase 2	+	+	-	+	+
MAP2K3	mitogen-activated protein kinase kinase 3	+	+	-	+	+
MAP2K4	mitogen-activated protein kinase kinase 4	+	+	-	+	+
MAP2K7	mitogen-activated protein kinase kinase 7	+	+	-	+	+
MAP3K1	mitogen-activated protein kinase kinase kinase 1	+	+	-	+	+
MAP3K3	mitogen-activated protein kinase kinase kinase 3	+	-	-	+	-
MAP3K5	mitogen-activated protein kinase kinase kinase 5	-	+	-	+	-
MAP3K7	mitogen-activated protein kinase kinase kinase 7	+	+	-	+	+
MAP3K8	mitogen-activated protein kinase kinase kinase 8	+	+	-	+	+
MAPK1	mitogen-activated protein kinase 1	+	+	-	+	+
MAPK13	mitogen-activated protein kinase 13	+	+	-	+	+
MAPK14	mitogen-activated protein kinase 14	+	+	-	+	+
MAPK8	mitogen-activated protein kinase 8	+	+	-	+	+
MAPK9	mitogen-activated protein kinase 9	+	+	-	+	+
MAPKAPK2	MAPK activated protein kinase 2	+	-	-	+	+
MARCKS	myristoylated alanine rich protein kinase C substrate	-	-	-	+	-
MARCO	macrophage receptor with collagenous structure	-	-	-	+	-
MAVS	mitochondrial antiviral signaling protein	-	-	-	-	+
MCL1	MCL1 apoptosis regulator, BCL2 family member	+	+	-	+	-
MDFIC	MyoD family inhibitor domain containing	+	-	-	-	-

MEFV	MEFV innate immuity regulator, pyrin	-	-	-	-	+
MGAM	maltase-glucoamylase	-	-	-	+	-
MIF	macrophage migration inhibitory factor	+	-	-	+	-
MKNK1	MAPK interacting serine/threonine kinase 1	-	+	-	-	-
MLKL	mixed lineage kinase domain like pseudokinase	-	+	-	+	-
MME	membrane metalloendopeptidase	-	+	+	+	-
MRC1	mannose receptor C-type 1	+	-	-	+	-
MS4A1	membrane spanning 4-domains A1	+	-	-	-	-
MS4A2	membrane spanning 4-domains A2	-	-	-	+	-
MS4A4A	membrane spanning 4-domains A4A	-	-	-	+	-
MS4A7	membrane spanning 4-domains A7	-	-	-	+	-
MSRA	methionine sulfoxide reductase A	-	+	-	+	-
MT2A	metallothionein 2A	-	-	-	-	+
MTOR	mechanistic target of rapamycin kinase	+	+	-	-	-
MVP	major vault protein	-	-	-	+	-
MX1	MX dynamin like GTPase 1	-	-	-	-	+
MYC	MYC proto-oncogene, bHLH transcription factor	+	+	-	+	-
MYD88	MYD88 innate immune signal transduction adaptor	+	-	-	+	+
NAE1	NEDD8 activating enzyme E1 subunit 1	-	+	-	-	-
NAMPT	nicotinamide phosphoribosyltransferase	-	-	-	+	+
NCF1	neutrophil cytosolic factor 1	+	-	-	+	-
NCF2	neutrophil cytosolic factor 2	+	+	-	+	-
NCF4	neutrophil cytosolic factor 4	+	+	-	+	-
NCR1	natural cytotoxicity triggering receptor 1	-	-	-	+	-
NCR3	natural cytotoxicity triggering receptor 3	-	-	-	+	-

NDUFS8	NADH:ubiquinone oxidoreductase core subunit S8	-	+	-	+	-
NEO1	neogenin 1	+	+	-	+	-
NEU1	neuraminidase 1	-	+	-	+	-
NFAT5	nuclear factor of activated T cells 5	+	-	-	+	-
NFATC1	nuclear factor of activated T cells 1	+	-	-	+	+
NFATC2	nuclear factor of activated T cells 2	+	-	-	+	+
NFATC3	nuclear factor of activated T cells 3	+	-	-	-	+
NFATC4	nuclear factor of activated T cells 4	+	-	-	+	-
NFE2L2	nuclear factor, erythroid 2 like 2	-	+	-	+	-
NFKB1	nuclear factor kappa B subunit 1	+	+	-	+	+
NFKB2	nuclear factor kappa B subunit 2	+	-	-	+	+
NGLY1	N-glycanase 1	-	+	-	-	-
NKG7	natural killer cell granule protein 7	+	-	-	+	-
NLRC4	NLR family CARD domain containing 4	-	-	-	-	+
NLRC5	NLR family CARD domain containing 5	-	-	-	-	+
NLRP1	NLR family pyrin domain containing 1	-	-	-	-	+
NLRP3	NLR family pyrin domain containing 3	-	-	-	-	+
NOD2	nucleotide binding oligomerization domain containing 2	+	+	-	+	+
NOS2	nitric oxide synthase 2	+	+	-	+	-
NOTCH1	notch receptor 1	+	-	-	-	-
NOX1	NADPH oxidase 1	-	+	-	+	-
NPC2	NPC intracellular cholesterol transporter 2	-	+	-	+	-
NRAS	NRAS proto-oncogene, GTPase	+	-	-	+	+
NT5E	5'-nucleotidase ecto	-	-	-	+	-
NTNG2	netrin G2	+	-	-	-	-
OAS1	2'-5'-oligoadenylate synthetase 1	-	-	-	-	+
OAS2	2'-5'-oligoadenylate synthetase 2	-	-	-	-	+

OAS3	2'-5'-oligoadenylate synthetase 3	-	-	-	-	+
OASL	2'-5'-oligoadenylate synthetase like	-	-	-	-	+
OS9	OS9 endoplasmic reticulum lectin	-	+	-	-	-
OSM	oncostatin M	+	-	-	+	+
P2RX7	purinergic receptor P2X 7	-	-	-	-	+
PAK1	p21 (RAC1) activated kinase 1	+	-	-	+	+
PANX1	pannexin 1	-	-	-	-	+
PARP1	poly(ADP-ribose) polymerase 1	+	+	-	+	-
PARP9	poly(ADP-ribose) polymerase family member 9	-	-	-	-	+
PDCD1	programmed cell death 1	+	-	-	-	-
PDCD1LG2	programmed cell death 1 ligand 2	+	-	-	-	-
PDHB	pyruvate dehydrogenase E1 subunit beta	-	+	-	-	-
PECAM1	platelet and endothelial cell adhesion molecule 1	+	+	-	+	-
PELI1	pellino E3 ubiquitin protein ligase 1	+	-	-	+	+
PELI2	pellino E3 ubiquitin protein ligase family member 2	+	-	-	+	+
PFKFB3	6-phosphofructo-2-kinase/fructose-2,6-biphosphatase 3	-	+	-	-	-
PIK3C3	phosphatidylinositol 3-kinase catalytic subunit type 3	-	+	-	+	+
PIK3CA	phosphatidylinositol-4,5-bisphosphate 3-kinase catalytic subunit alpha	+	+	-	+	-
PIK3CB	phosphatidylinositol-4,5-bisphosphate 3-kinase catalytic subunit beta	+	+	-	+	-
PIK3CD	phosphatidylinositol-4,5-bisphosphate 3-kinase catalytic subunit delta	+	+	-	+	-
PIK3CG	phosphatidylinositol-4,5-bisphosphate 3-kinase catalytic subunit gamma	-	+	-	+	-
PIK3R3	phosphoinositide-3-kinase regulatory subunit 3	+	+	-	+	-

PIK3R4	phosphoinositide-3-kinase regulatory subunit 4	-	+	-	-	+
PIK3R5	phosphoinositide-3-kinase regulatory subunit 5	-	+	-	+	-
PIK3R6	phosphoinositide-3-kinase regulatory subunit 6	-	+	-	+	-
PLAT	plasminogen activator, tissue type	+	+	-	-	-
PLAU	plasminogen activator, urokinase	+	+	-	+	-
PLAUR	plasminogen activator, urokinase receptor	+	+	-	+	-
PLCG1	phospholipase C gamma 1	+	+	-	+	+
PLCG2	phospholipase C gamma 2	+	-	-	+	+
PLEK	pleckstrin	-	+	-	-	-
PLEKHA1	pleckstrin homology domain containing A1	-	+	-	+	-
PLG	plasminogen	+	+	+	+	-
PLIN4	perilipin 4	-	-	-	+	-
PNOC	prepronociceptin	-	-	-	-	-
PPIA	peptidylprolyl isomerase A	+	+	+	+	-
PRCP	prolylcarboxypeptidase	-	+	+	+	-
PRDM1	PR/SET domain 1	+	-	-	-	-
PRF1	perforin 1	+	+	-	+	-
PRKCA	protein kinase C alpha	+	+	-	+	-
PRKCD	protein kinase C delta	-	-	-	+	+
PRKCQ	protein kinase C theta	+	+	-	+	-
PRKCSH	protein kinase C substrate 80K-H	-	+	-	-	-
PSAP	prosaposin	-	+	-	+	-
PSEN1	presenilin 1	-	+	-	+	-
PSMB10	proteasome 20S subunit beta 10	+	-	-	+	+
PSMB8	proteasome 20S subunit beta 8	+	-	-	+	+

PSMB9	proteasome 20S subunit beta 9	+	-	-	+	+
PSTPIP1	proline-serine-threonine phosphatase interacting protein 1	-	-	-	-	+
PTGER2	prostaglandin E receptor 2	+	+	-	-	-
PTGER4	prostaglandin E receptor 4	-	+	-	-	-
PTGS2	prostaglandin-endoperoxide synthase 2	+	+	-	+	-
PTK2B	protein tyrosine kinase 2 beta	+	-	-	+	-
PTPN4	protein tyrosine phosphatase non-receptor type 4	+	-	-	+	+
PTPN6	protein tyrosine phosphatase non-receptor type 6	+	-	-	+	+
PTPRC	protein tyrosine phosphatase receptor type C	+	-	-	+	-
PXN	paxillin	+	+	-	+	-
PYCARD	PYD and CARD domain containing	-	-	-	+	+
RAB31	RAB31, member RAS oncogene family	-	-	-	+	-
RAB5C	RAB5C, member RAS oncogene family	-	-	-	+	-
RAB7A	RAB7A, member RAS oncogene family	+	-	-	+	-
RAC2	Rac family small GTPase 2	+	-	-	+	-
RACK1	receptor for activated C kinase 1	-	+	-	+	-
RAF1	Raf-1 proto-oncogene, serine/threonine kinase	+	+	-	+	+
RASGRP1	RAS guanyl releasing protein 1	+	+	-	+	-
RASGRP4	RAS guanyl releasing protein 4	-	-	-	+	-
RB1CC1	RB1 inducible coiled-coil 1	-	+	-	-	-
RBCK1	RANBP2-type and C3HC4-type zinc finger containing 1	+	+	-	+	+
RBPJ	recombination signal binding protein for immunoglobulin kappa J region	+	-	-	+	-
REL	REL proto-oncogene, NF-kB subunit	+	-	-	+	-
RELA	RELA proto-oncogene, NF-kB subunit	+	+	-	+	+

RELB	RELB proto-oncogene, NF-kB subunit	+	-	-	+	+
RGMA	repulsive guidance molecule BMP co-receptor a	+	+	-	+	-
RHOG	ras homolog family member G	-	+	-	+	-
RIPK1	receptor interacting serine/threonine kinase 1	+	+	-	+	+
RIPK2	receptor interacting serine/threonine kinase 2	+	-	-	+	+
RIPK3	receptor interacting serine/threonine kinase 3	-	+	-	+	+
RNASEL	ribonuclease L	-	-	-	-	+
RNF114	ring finger protein 114	+	-	-	-	-
RNF135	ring finger protein 135	-	-	-	-	+
RNF31	ring finger protein 31	-	+	-	+	+
RPS6KA1	ribosomal protein S6 kinase A1	+	+	-	+	+
RPS6KA3	ribosomal protein S6 kinase A3	+	+	-	+	+
RPS6KB1	ribosomal protein S6 kinase B1	+	+	-	+	-
RSAD2	radical S-adenosyl methionine domain containing 2	-	-	-	-	+
RUNX3	RUNX family transcription factor 3	+	-	-	-	-
S100A12	S100 calcium binding protein A12	+	-	-	+	+
SAMHD1	SAM and HD domain containing deoxynucleoside triphosphate triphosphohydrolase 1	-	-	-	-	+
SCARB2	scavenger receptor class B member 2	-	+	-	-	-
SELE	selectin E	+	+	-	+	-
SELENOS	selenoprotein S	-	+	-	+	-
SELL	selectin L	+	-	-	+	-
SEM1	SEM1 26S proteasome complex subunit	+	-	-	+	+
SERPINA1	serpin family A member 1	+	+	-	+	-
SH2D1A	SH2 domain containing 1A	-	-	-	+	-

SIGIRR	single Ig and TIR domain containing	+	-	-	+	+
SIGLEC5	sialic acid binding Ig like lectin 5	-	-	-	+	-
SIRPA	signal regulatory protein alpha	+	+	-	+	-
SLC11A1	solute carrier family 11 member 1	-	+	-	+	-
SLC2A3	solute carrier family 2 member 3	-	-	-	+	-
SMAD3	SMAD family member 3	+	+	-	+	-
SMAD4	SMAD family member 4	+	+	-	+	-
SMAD5	SMAD family member 5	+	+	-	+	-
SOCS1	suppressor of cytokine signaling 1	+	-	-	+	+
SOCS3	suppressor of cytokine signaling 3	+	+	-	+	+
SOD1	superoxide dismutase 1	+	+	-	+	-
SOD2	superoxide dismutase 2	+	+	-	+	-
SORT1	sortilin 1	-	+	-	-	-
SP1	Sp1 transcription factor	+	+	-	+	-
SP100	SP100 nuclear antigen	-	-	-	-	+
SPI1	Spi-1 proto-oncogene	+	-	-	+	-
SPIB	Spi-B transcription factor	-	-	-	-	-
SSR1	signal sequence receptor subunit 1	-	+	-	-	-
STAT1	signal transducer and activator of transcription 1	+	-	-	+	+
STAT2	signal transducer and activator of transcription 2	-	-	-	-	+
STAT3	signal transducer and activator of transcription 3	+	+	-	+	-
STAT4	signal transducer and activator of transcription 4	+	-	-	+	-
STAT5A	signal transducer and activator of transcription 5A	+	-	-	+	-
STAT5B	signal transducer and activator of transcription 5B	+	-	-	+	-

STAT6	signal transducer and activator of transcription 6	+	-	-	+	+
STING1	stimulator of interferon response cGAMP interactor 1	-	-	-	+	+
STRAP	serine/threonine kinase receptor associated protein	+	+	-	+	-
STT3B	STT3 oligosaccharyltransferase complex catalytic subunit B	-	+	-	-	-
SUGT1	SGT1 homolog, MIS12 kinetochore complex assembly cochaperone	-	-	-	-	+
SYK	spleen associated tyrosine kinase	+	-	-	+	+
TAB1	TGF-beta activated kinase 1 (MAP3K7) binding protein 1	+	+	-	+	+
TAB2	TGF-beta activated kinase 1 (MAP3K7) binding protein 2	+	+	-	+	+
TANK	TRAF family member associated NFKB activator	-	-	-	-	+
TAP1	transporter 1, ATP binding cassette subfamily B member	+	-	-	+	-
TAP2	transporter 2, ATP binding cassette subfamily B member	+	-	-	+	-
TBK1	TANK binding kinase 1	+	-	-	+	+
TBX21	T-box transcription factor 21	+	-	-	-	-
TBXAS1	thromboxane A synthase 1	-	+	-	-	-
TCF7	transcription factor 7	+	-	-	-	-
TCIRG1	T cell immune regulator 1, ATPase H+ transporting V0 subunit a3	-	+	-	+	-
TCL1A	TCL1 family AKT coactivator A	-	-	-	-	-
TCN2	transcobalamin 2	-	-	-	+	-
TGFB1	transforming growth factor beta 1	+	+	-	+	-
TGFB2	transforming growth factor beta 2	+	+	-	+	-
TGFB3	transforming growth factor beta 3	+	+	-	+	-

TGFBR2	transforming growth factor beta receptor 2	+	+	-	+	-
THBS1	thrombospondin 1	+	+	-	+	-
THOP1	thimet oligopeptidase 1	+	+	+	-	-
TIFA	TRAF interacting protein with forkhead associated domain	-	-	-	-	+
TIGIT	T cell immunoreceptor with Ig and ITIM domains	+	-	-	-	-
TIMP2	TIMP metallopeptidase inhibitor 2	-	-	-	+	-
TLN1	talin 1	-	+	-	-	-
TLR1	toll like receptor 1	+	-	-	+	+
TLR2	toll like receptor 2	+	-	+	+	+
TLR3	toll like receptor 3	-	-	-	+	+
TLR4	toll like receptor 4	+	+	-	+	+
TLR5	toll like receptor 5	-	-	-	-	+
TLR6	toll like receptor 6	+	-	-	+	+
TLR7	toll like receptor 7	-	-	-	+	+
TLR8	toll like receptor 8	-	-	-	+	+
TLR9	toll like receptor 9	-	-	-	-	+
TMEM140	transmembrane protein 140	-	-	-	-	+
TMPRSS2	transmembrane serine protease 2	-	-	+	-	-
TNF	tumor necrosis factor	+	+	-	+	+
TNFRSF10B	TNF receptor superfamily member 10b	+	+	-	+	-
TNFRSF17	TNF receptor superfamily member 17	+	-	-	+	-
TNFRSF18	TNF receptor superfamily member 18	+	-	-	+	-
TNFRSF1A	TNF receptor superfamily member 1A	+	+	-	+	-
TNFRSF25	TNF receptor superfamily member 25	+	-	-	+	-
TNFRSF4	TNF receptor superfamily member 4	+	-	-	+	-
TNFRSF9	TNF receptor superfamily member 9	+	-	-	+	-
TNFSF10	TNF superfamily member 10	+	+	-	+	-

TNFSF13B	TNF superfamily member 13b	+	-	-	+	-
TNFSF18	TNF superfamily member 18	+	-	-	+	-
TNFSF4	TNF superfamily member 4	+	-	-	+	-
TNFSF9	TNF superfamily member 9	+	-	-	+	-
TOLLIP	toll interacting protein	+	-	-	+	+
TPP1	tripeptidyl peptidase 1	-	+	-	-	-
TPSAB1/B2	tryptase alpha/beta 1/2	-	-	+	-	-
TRAF2	TNF receptor associated factor 2	+	+	-	+	+
TRAF3	TNF receptor associated factor 3	+	+	-	+	+
TRAF6	TNF receptor associated factor 6	+	-	-	+	+
TRAM1	translocation associated membrane protein 1	-	+	-	-	-
TRAT1	T cell receptor associated transmembrane adaptor 1	+	-	-	-	-
TRIM21	tripartite motif containing 21	+	-	-	-	+
TRIM22	tripartite motif containing 22	-	-	-	-	+
TRIM25	tripartite motif containing 25	+	-	-	+	+
TRIM33	tripartite motif containing 33	+	+	-	+	-
TRIM5	tripartite motif containing 5	-	-	-	-	+
TRIM56	tripartite motif containing 56	-	-	-	-	+
TRIM6	tripartite motif containing 6	-	-	-	-	+
TXK	TXK tyrosine kinase	+	-	-	+	-
TXN	thioredoxin	-	+	-	+	+
TXNIP	thioredoxin interacting protein	-	+	-	+	+
TYK2	tyrosine kinase 2	+	-	-	+	+
TYROBP	transmembrane immune signaling adaptor TYROBP	-	-	-	+	-
UBA52	ubiquitin A-52 residue ribosomal protein fusion product 1	+	+	-	+	+
UBE2L6	ubiquitin conjugating enzyme E2 L6	+	-	-	-	+

UBE2N	ubiquitin conjugating enzyme E2 N	+	-	-	+	+
ULK1	unc-51 like autophagy activating kinase 1	-	+	-	-	-
ULK2	unc-51 like autophagy activating kinase 2	-	+	-	-	-
VAMP3	vesicle associated membrane protein 3	+	-	-	+	-
VCAM1	vascular cell adhesion molecule 1	+	+	-	+	+
VEGFA	vascular endothelial growth factor A	+	+	-	+	-
VRK3	VRK serine/threonine kinase 3	+	-	-	+	+
VSIR	V-set immunoregulatory receptor	+	-	-	-	-
VWF	von Willebrand factor	+	+	-	-	-
WAS	WASP actin nucleation promoting factor	+	-	-	+	-
WIPI1	WD repeat domain, phosphoinositide interacting 1	-	+	-	-	-
XAF1	XIAP associated factor 1	-	-	-	-	+
XBP1	X-box binding protein 1	-	+	-	+	-
XCL1/2	X-C motif chemokine ligand 1/2	+	-	-	+	-
XCR1	X-C motif chemokine receptor 1	-	-	-	+	-
YWHAQ	tyrosine 3-monooxygenase/tryptophan 5-monooxygenase activation protein theta	-	+	-	-	-
ZAP70	zeta chain of T cell receptor associated protein kinase 70	+	-	-	+	-
ZBP1	Z-DNA binding protein 1	-	-	-	-	+
Housekeeping genes						
ABCF1	ATP binding cassette subfamily F member 1					
ALAS1	5'-aminolevulinate synthase 1					
GUSB	glucuronidase beta					
HPRT1	hypoxanthine phosphoribosyltransferase 1					

MRPS7	mitochondrial ribosomal protein S7					
NMT1	N-myristoyltransferase 1					
NRDE2	NRDE-2, necessary for RNA interference, domain containing					
OAZ1	ornithine decarboxylase antizyme 1					
PGK1	phosphoglycerate kinase 1					
SDHA	succinate dehydrogenase complex flavoprotein subunit A					
STK11IP	serine/threonine kinase 11 interacting protein					
TBP	TATA-box binding protein					

HUGO: Human Genome Organisation; COX-2, cyclooxygenase-2

Table S2: Additional probes spiked into the panel

Customer Identifier	**HUGO Gene**	**NSID**
adrA	*adrA*	ECL_RS05565.1:625
clfA	*clfA*	USA300HOU_0819.1:170
clfB	*clfB*	USA300HOU_2630.1:519
csgB	*csgB*	ECL_RS12735.1:130
CupA1	*cupA1*	PA2128.2:447
CupA4	*cupA4*	PA2131.2:845
E.cloacae	*Enterobacter_cloacae_16S*	NR_102794.2:539
E. faecalis	*Enterococcus_faecalis_16S*	NR_040789.1:389
flgM	*flgM*	ECL_RS12590.1:175
fliA	*fliA*	ECL_RS16130.1:180
fnbA	*fnbA*	USA300HOU_2491.1:1731
fnbB	*fnbB*	USA300HOU_2490.1:282
lecA	*lecA*	PA2570.2:158
lecB	*lecB*	PA3361.2:145
P. aeruginosa	*Pseudomonas_aeruginosa_16S*	NR_026078.1:514
S. aureus	*Staphylococcus_aureus_16S*	NR_118997.2:397
S. epidermidis	*Staphylococcus_epidermidis_16S*	NR_036904.1:372

HUGO: Human Genome Organisation; NSID: Nanostring internal identifiers

Table S3: detailed table 3 with the addition of biofilm genes

Group	Patient	Conventional culture	type of sample	*Staphylococcus aureus*	*clfA*	*clfB*	*fnbA*	*fnbB*	*icaA*	*icaB*	*icaC*	*Staphylococcus epidermidis*	*Enterococcus faecalis*	*Enterobacter cloacae*	*adrA*	*csgB*	*flgM*	*fliA*	*Pseudomonas aeruginosa*	*CupA1*	*CupA4*	*lecA*	*lecB*	16S rRNA
Infected	2	*S.aureus*	*unknown*	251	1	2	2	4	1	2	2	106	1	2	2	3	2	1	1	2	2	2	2	*S. aureus*
	3	*S.aureus*	*unknown*	25	2	1	3	0	36	3	4	15	1	0	1	44	9	1	0	3	1	1	2	*S. aureus*
	13	*S.aureus*	*tissue*	3	1	3	2	4	2	2	3	3	2	2	2	5	2	1	1	2	2	3	1	*S. aureus*
	25	*S.aureus*	*bone*	2	2	1	1	1	2	1	2	1	1	1	2	1	1	1	1	3	1	1	4	*negative*
	29	*MRSA*	*tissue*	29147	16	119	70	154	151	119	114	15964	2	0	3	1	0	4	0	0	11	3	1	*S. aureus*
	32	*S. aureus*	*tissue*	4	1	1	1	0	4	2	1	2	0	1	2	3	2	1	1	1	1	2	2	*S. aureus*
	6	*negative****	*tissue*	8	1	1	2	2	3	2	2	7	0	2	1	1	1	1	1	3	2	2	4	*S. aureus*
	10	*S. epidermidis/S. capitis/S. caprae/Bacillus sp.*	*tissue*	2	1	1	2	2	6	1	1	2	1	1	2	1	2	1	1	1	2	2	1	*Staphylococcus sp.*
	12	*E.faecalis*	*bone*	1	2	1	4	0	24	6	4	5	4	1	2	30	9	1	1	3	2	4	2	*E. faecalis*
	14	*P.aeruginosa*	*bone*	2	1	1	2	2	3	2	1	1	0	1	2	2	2	1	1	3	2	2	2	*Negative*
	15	*S. lugdunensis*	*tissue*	2	1	2	1	2	3	2	2	2	1	1	2	2	1	2	1	2	2	1	2	*no probe included*
	19	*S. epidermidis*	*tissue*	3	1	2	1	3	3	1	2	2	1	1	1	1	1	1	1	1	2	2	2	*Staphylococcus sp.*
	7	*E. cloacae*	*bone*	1	1	1	4	1	3	7	16	8	1	149	2	1	1	1	38	2	1	3	2	*E. cloacae/P. aeruginosa*
	18	*E. cloacae*	*tissue*	0	1	0	0	0	1	0	1	1	1	2	0	2	1	0	0	0	1	1	1	*negative*
	33	*C. acnes***	*unknown*	2	0	1	2	0	4	1	2	2	0	2	1	4	1	1	2	2	1	2	1	*no probe included*
Control	1	*negative*	*bone*	1	1	2	2	0	13	3	5	3	1	1	1	15	4	1	1	2	1	2	5	*negative*
	4	*negative*	*tissue*	8	0	1	2	3	6	1	2	6	2	2	3	2	2	2	2	1	2	2	6	*S. aureus*
	5	*negative*	*bone*	1	1	1	1	2	3	1	2	1	1	1	2	1	1	1	1	2	1	1	3	*negative*
	8	*S.epidermidis**	*tissue*	3	2	1	2	1	5	2	1	3	1	1	1	4	1	2	1	2	2	1	2	*S. aureus*
	9	*negative*	*bone*	1	1	1	1	4	2	1	2	1	1	1	1	0	1	1	1	2	1	0	1	*negative*
	11	*negative*	*bone*	2	1	2	1	2	3	0	1	1	1	1	2	1	1	2	1	4	3	2	3	*negative*
	16	*S.epidermidis/Bacillus sp.**	*bone*	1	0	1	1	2	1	1	1	1	0	1	1	1	1	1	1	1	1	1	1	*negative*
	17	*Bacillus species**	*bone*	0	1	1	0	1	1	1	1	0	0	0	0	0	0	0	0	1	1	1	2	*negative*
	20	*negative*	*bone*	2	1	2	1	2	3	2	2	1	1	1	2	3	2	1	1	3	2	2	1	*negative*
	22	*negative*	*bone*	21	1	2	2	2	1	2	1	16	1	1	2	0	1	1	1	1	2	1	2	*S. aureus*
	24	*negative*	*bone*	**7**	2	1	1	**3**	**5**	1	1	6	1	1	2	1	1	2	1	2	3	2	2	*S. aureus*
	26	*negative*	*tissue*	1	2	1	2	0	4	**3**	2	1	1	0	2	4	1	2	0	2	2	2	4	*negative*
	27	*negative*	*bone*	2	1	2	2	**3**	4	2	2	1	2	1	2	2	2	1	1	5	1	1	2	*negative*
	30	*Bacillus cereus**	*bone*	1	1	2	0	0	1	1	2	1	0	1	1	1	2	1	1	0	1	1	1	*negative*
	31	*negative*	*tissue*	1	0	1	1	1	1	1	1	1	1	1	0	1	1	2	1	2	1	1	1	*negative*
	34	*negative*	*sonicate*	1	0	1	1	1	0	1	2	1	0	1	1	1	0	1	1	2	1	1	2	*negative*
Failed analysis	21	*S. aureus*	*unknown*																					
	23	*negative*	*tissue*																					
	28	*negative*	*tissue*																					

S. aureus: Staphylococcus aureus; S. epidermidis: Staphylococcus epidermidis; S. caprae: Staphylococcus caprae; S. capitis: Staphylococcus capitis; C. acnes: Cutibacterium acnes; MRSA: Methicillin-Resistant Staphylococcus aureus; E. cloacae; Enterobacter cloacae; P. aeruginosa: Pseudomonas aeruginosa; Sp: species

*considered as contaminants (classified as suggestive pathogens, but considered contaminants after evaluation by the multidisciplinary team) ** no probes included*** considered as false-negative culture result

No data were shown for patients 21, 23, and 28 because the transcriptomic analyses for these samples failed due to QC error.

Table S4: Comparison between infected FRI patients (n=15) and a non-infected control group (n=16). P-values were adjusted using the Benjamini–Hochberg false discovery rate (FDR). Significant genes are indicated in bold. (-log (p-adjusted > 1.3)).

Gene	log2FoldChange	-log(p-adjusted)
GK	3.387673	2.235302
PTGS2	3.599788	2.235302
SOD2	2.806119	1.767157
LCP2	2.937850	1.767157
PFKFB3	2.697631	1.720202
SLC2A3	2.782975	1.720202
CCL4/L1/L2	2.941441	1.680506
MEFV	2.419760	1.603211
CEACAM3	1.681630	1.465615
CCL24	-1.634316	1.397076
CD45RB	1.235577	1.397076
RSAD2	1.377099	1.397076
PTK2B	1.611161	1.397076
NOD2	2.064391	1.397076
GBP5	2.111791	1.397076
NAMPT	2.344139	1.397076
SIGLEC5	2.381743	1.397076
CASP3	2.517509	1.397076
XBP1	1.640557	1.388655
FPR2	2.232200	1.388655
PLAUR	2.449448	1.349184
VEGFA	2.454637	1.349184
BCL3	2.029499	1.269365
CXCL2	2.334576	1.267263
CD44	1.470807	1.255552
ADORA2A	1.678072	1.255552
PIK3R5	2.069393	1.255552
LIMK2	2.100246	1.255552
CYSTM1	1.437602	1.232119
ZBP1	1.855418	1.232119
NLRP3	1.941771	1.195742
LRG1	2.110615	1.195742
PRKCD	1.746159	1.173659
SERPINA1	2.115013	1.164505
IL2RG	1.370161	1.120070
IRF7	1.500889	1.082543
IRAK3	1.763629	1.072536
IFIT2	1.412168	1.070878
CSF2RB	1.475774	1.070878

KDM6B	1.832416	1.070878
TRAF3	1.840492	1.070878
NCF2	1.959300	1.070878
PLAT	-1.641132	1.055706
LITAF	1.560517	1.055706
ITGAX	1.873867	1.055706
ATG7	1.909088	1.055706
CR1	1.911488	1.049878
CEBPB	1.012592	1.029796
GBP2	1.114293	1.029796
CSF2RA	1.185368	1.029796
HCK	1.285754	1.029796
CD14	1.296710	1.029796
IFITM2	1.484870	1.029796
IL6R	1.488129	1.029796
NCF1	1.558913	1.029796
IL18R1	1.768104	1.029796
LYN	1.819382	1.029796
ALOX5AP	1.924616	1.020579
IFITM1	1.207840	1.008159
REL	1.318386	1.008159
NFKB1	1.501546	1.008159
GADD45B	1.674853	1.008159
FGR	1.693711	1.008159
C3AR1	1.780205	1.008159
FCGR2A	1.542928	0.984384
TLR1	1.049844	0.961614
TLR6	1.347351	0.961614
FCAR	1.720899	0.961614
SLC11A1	1.817347	0.961614
DDIT3	1.865491	0.961614
CCL18	3.385547	0.961614
IFIH1	1.072027	0.937783
ISG15	1.559286	0.937330
RASGRP4	1.213404	0.935675
CASP4	1.593989	0.935675
CXCL8	2.608016	0.935675
HERC5	1.374880	0.932641
NFKB2	1.577487	0.932641
PLAU	2.312291	0.926290
ALOX5	1.395321	0.915734
CXCR2	1.148799	0.915433
IL4R	1.150744	0.912710
IRF1	1.161837	0.902973
IL1RAP	1.475907	0.902973

RAC2	1.557022	0.902973
IFNGR2	1.343594	0.896748
ERN1	1.480977	0.896748
BCL6	1.561038	0.896748
CTLA4	0.993574	0.895371
KRAS	1.170878	0.895371
FCGR3A/B	1.218190	0.895371
IFIT3	1.221694	0.895371
IFIT1	1.228765	0.895371
IRF9	1.311825	0.895371
TLR2	1.399476	0.895371
JAK3	1.452252	0.895371
CXCL3	1.462381	0.895371
LILRA5	1.465744	0.895371
FPR1	1.647239	0.895371
DEFA1	2.211230	0.895371
OSM	2.529563	0.895371
IL1B	2.611527	0.895371
PAK1	1.459742	0.880846
GPX7	-1.109713	0.860408
ARRB2	1.496904	0.858356
TNFSF13B	1.359342	0.852949
SOCS3	1.578194	0.844164
KLRC1	1.084522	0.841303
PYCARD	1.067399	0.824900
NCF4	1.368179	0.809911
TRIM25	1.311246	0.788646
TANK	1.268113	0.781476
FOXP3	-0.906891	0.770331
NLRP1	0.584837	0.770331
NGLY1	0.926231	0.770331
HLA-DRB	0.935310	0.770331
CARD17	1.188958	0.770331
CD69	1.382616	0.770331
ADAR	1.760723	0.770331
TAP1	0.886072	0.764640
RHOG	0.981108	0.764640
NLRC4	0.999553	0.764640
ATP6V1B2	1.235421	0.764640
GCA	1.298980	0.764640
LCP1	1.457597	0.764640
PIK3CD	1.107339	0.763452
RAB7A	0.826489	0.756739
TBK1	1.245112	0.756739
IL6	1.429995	0.756739

CASP1	1.131403	0.755187
GZMA	1.190515	0.748766
CD274	0.851137	0.748087
AIM2	0.823072	0.744318
HK3	0.855182	0.737066
IFITM3	0.868300	0.734503
SYK	0.916025	0.734503
CCR1	1.385323	0.727813
CD45R0	1.297280	0.722568
C5AR1	1.316175	0.710999
TRAF6	0.877811	0.707214
MARCO	1.339695	0.705078
MLKL	0.830075	0.705057
STAT1	1.132902	0.705057
PTGER4	0.856115	0.697899
LILRB2	1.109286	0.696951
STAT3	1.127215	0.696951
CXCL5	1.390225	0.696951
CXCL1	1.828931	0.696951
HSP90B1	0.889063	0.695693
FAS	0.934723	0.695693
DDX58	0.982763	0.695693
CD86	1.147557	0.692753
APBB1IP	1.107360	0.690957
AIF1	0.892348	0.690130
LTA4H	0.882197	0.685738
TNFRSF10B	1.127708	0.684412
DYSF	1.087796	0.682956
ACSL1	1.883273	0.682956
TYK2	0.584573	0.681131
TAP2	0.519542	0.678309
MAPK14	0.702502	0.677773
IL10RA	0.825324	0.677773
LTBR	0.955181	0.677773
BCL2	0.986816	0.677773
STAT5B	0.699005	0.676597
S100A12	1.322264	0.676154
MAPK13	0.838102	0.674338
CCL14	-1.152440	0.672906
PRDM1	0.807754	0.672906
MCL1	1.071809	0.672906
ITGAM	1.100359	0.672906
GUCY1B1	1.155075	0.672906
IL10RB	1.048255	0.668440
FURIN	0.941268	0.662459

CD36	-1.210389	0.661713
CFLAR	0.886738	0.661713
JUNB	0.988294	0.661713
BECN1	-0.602430	0.656286
TOLLIP	0.880480	0.656286
NFE2L2	0.992434	0.656286
LTB	0.999877	0.656286
ATG3	1.103624	0.656286
IGHG	2.496990	0.656286
CD22	1.034881	0.642788
PTPRC	1.001557	0.625041
CD27	1.016147	0.625041
IFI6	1.133866	0.625041
SELL	1.092249	0.624492
PIK3CG	0.919521	0.613995
MARCKS	1.026822	0.611318
TRIM21	1.299035	0.609188
FCGR1A/B	1.045322	0.608677
CXCR1	1.023497	0.607463
MYD88	0.908956	0.598731
JAK2	0.980433	0.594435
RIPK2	0.781417	0.594131
ACOX1	0.773052	0.592139
RBPJ	0.770637	0.584358
CD28	0.929863	0.584358
IFI35	0.982722	0.584358
SPI1	1.018802	0.584358
SP100	1.036939	0.584358
GUCY1A1	0.837729	0.582186
CXCR4	1.016217	0.581967
HSP90AA1	0.605991	0.580317
IL18	0.675595	0.580317
CRK	0.705628	0.580317
TBXAS1	1.090925	0.580317
CCR2	0.887961	0.568290
IGHA	1.642079	0.568290
DIABLO	0.720577	0.567171
RPS6KA1	0.942488	0.567171
CBL	0.962607	0.567171
C3	1.698324	0.567171
RIPK3	0.532394	0.565423
PSMB9	0.720461	0.556613
IL7R	0.723497	0.556606
IL1R2	1.099241	0.556606
LAT2	0.973776	0.544497

TLR5	0.834375	0.538943
OAS2	0.891298	0.538943
DEFA4	0.894390	0.538943
PTPN6	0.909277	0.538943
CXCL16	1.001735	0.538943
IL1RN	2.015508	0.538943
STAT6	0.609436	0.538493
LRRK2	0.881348	0.533938
GSK3B	0.729634	0.531436
WAS	0.980297	0.531436
IL2	1.048715	0.531436
ATG10	0.766881	0.530723
JAML	0.971321	0.530723
CCL11	0.825947	0.526377
SIRPA	0.937187	0.526377
IFI27	0.864829	0.519308
NOX1	-0.876407	0.517637
MGAM	0.909089	0.517637
HLA-DRA	0.742808	0.511382
CASP10	0.785747	0.511382
APOBEC3G	0.873202	0.511382
CD3E	0.773932	0.502518
TRAC	0.903424	0.499625
TLR4	0.723700	0.493457
STRAP	1.356818	0.483962
TXN	0.652472	0.479589
ALPL	0.835481	0.474547
IRAK4	0.415037	0.473303
TRIM22	0.763387	0.473303
PLCG2	0.766829	0.473303
PSEN1	0.820821	0.473303
TNFRSF1A	1.446563	0.473303
CTSS	0.786000	0.470059
IL16	0.633143	0.468843
ALPK1	0.752995	0.467487
CCL3/L1/L3	1.651093	0.466324
GBP4	0.482125	0.464504
ATG12	0.542273	0.463117
ADGRE5	0.861944	0.460896
NOTCH1	0.750423	0.455942
IL17RA	0.576318	0.454201
MAP2K3	0.775319	0.454201
ZAP70	0.592083	0.448956
LCN2	0.955130	0.448956
HLA-B	0.604846	0.447671

XCR1	-0.671078	0.447527
TNFRSF25	0.662559	0.442592
TRIM33	0.410300	0.436436
SMAD4	0.504154	0.436436
PXN	0.592082	0.436436
HLA-DPA1	0.664851	0.436436
STAT5A	1.345688	0.436436
ATP6V0D1	0.731133	0.436232
CD163	0.779136	0.436232
WIPI1	0.695025	0.434074
XAF1	0.805622	0.432309
AP1S2	1.259060	0.430284
CSF3R	1.607269	0.429300
TPP1	0.566541	0.428304
GBA	0.753034	0.423347
VWF	-0.661700	0.422226
TLR9	-0.459432	0.421110
PIK3CB	0.719177	0.421110
BCL2L1	0.845620	0.415978
IFI44	0.689110	0.413690
IRAK1	0.745960	0.413690
FOS	0.826436	0.413516
HLA-C	0.543267	0.410994
HLA-A	0.598161	0.410994
ITGAL	0.727797	0.410994
APOL6	0.801619	0.410994
MS4A7	0.841925	0.410994
HLA-DQA	0.718282	0.409346
GLA	0.410748	0.401193
EIF2AK3	0.500181	0.392740
PRKCQ	0.431911	0.388056
HLX	0.537474	0.387562
TAB2	0.738949	0.384087
IRF4	-0.553375	0.378425
LTF	0.910930	0.378425
CCR3	0.503440	0.378223
PLEK	1.367043	0.378223
IL23R	0.650254	0.377696
ATM	1.280308	0.377696
DTX3L	0.431575	0.377619
ACKR3	0.740228	0.377619
TYROBP	0.752731	0.376622
HLA-DMA	0.742336	0.376232
IGHM	1.114849	0.376232
DERL1	1.186735	0.376232

CPA3	0.713900	0.374661
CUL1	0.497643	0.371831
F5	0.615716	0.371831
PIK3R4	0.620739	0.371831
PIK3CA	0.512635	0.371166
CASP8	0.628799	0.371166
CAP1	0.571567	0.369357
NEU1	0.484407	0.365924
SAMHD1	0.551748	0.364215
MAPKAPK2	0.541096	0.361620
HMGB1	0.319431	0.359143
CX3CR1	0.496413	0.359143
UBA52	0.504843	0.359143
RAF1	0.672873	0.359143
PTGER2	0.409272	0.355094
OAS1	0.444386	0.355094
PSTPIP1	0.476438	0.355094
RNASEL	0.523211	0.355094
MAP3K8	0.629067	0.355094
RBCK1	1.015847	0.355094
IFNAR2	1.040288	0.355094
IL2RB	0.590732	0.352930
CD3G	0.627088	0.352930
MAP3K1	0.594456	0.350374
IFNAR1	0.472546	0.348711
TNFSF10	0.790084	0.348711
LAMP2	0.614662	0.345006
NFATC3	0.963265	0.345006
ICAM3	0.447631	0.344706
SP1	0.466840	0.344645
KLRB1	0.680678	0.344645
RNF135	0.242253	0.344597
MRC1	0.580766	0.344597
IFNA8	0.615005	0.337091
LILRA6	0.484920	0.333976
OS9	0.407000	0.330730
DDX5	0.458289	0.330730
UBE2L6	0.499558	0.322140
SELE	0.602555	0.320789
IL1R1	0.499102	0.319267
PSMB10	0.477436	0.317056
MAPK9	0.750723	0.317056
PARP1	0.845124	0.317056
MAP2K7	0.568737	0.314061
MAP1LC3A	0.586495	0.314061

RB1CC1	0.462343	0.308955
KPNB1	0.625305	0.305963
NAE1	-0.396924	0.303144
SMAD5	0.415532	0.303098
TCIRG1	0.467201	0.298439
MIF	0.298853	0.297048
IL13RA1	0.382785	0.297048
MAPK1	0.394719	0.297048
PLIN4	0.534236	0.297048
MS4A4A	0.704544	0.297048
ATP6AP2	0.865604	0.297048
VRK3	0.716931	0.296655
ACSL4	0.817020	0.296655
CBFB	0.909804	0.296144
GSTM4	0.454239	0.290316
CSF1	0.481698	0.290316
PTPN4	0.378964	0.285222
AKT2	0.538612	0.285222
HLA-DMB	0.554540	0.285222
HLA-DPB1	0.584498	0.285222
CXCL9	0.538048	0.282032
ULK1	0.238565	0.280850
RAB31	0.998325	0.272180
ITPR3	-0.263487	0.265030
NDUFS8	0.863568	0.265030
ANPEP	0.480983	0.264045
RNF114	0.433120	0.254501
EGLN1	0.359450	0.254331
PARP9	0.437348	0.254331
PSAP	0.440956	0.254331
CCNC	0.442005	0.254331
TLR7	-0.223748	0.253669
CDH1	0.377839	0.253669
RNF31	0.424083	0.253669
VSIR	0.533352	0.253669
AKT1	0.788691	0.250938
SSR1	0.417381	0.248005
IKBKG	0.347374	0.245215
MSRA	0.405837	0.244577
PIK3C3	-0.210775	0.239378
ATG13	-0.203284	0.239378
MAP2K2	0.338806	0.239378
DNAJA2	0.417320	0.239378
CD3D	0.436851	0.235115
CSF1R	0.453180	0.229359

IL33	-0.506697	0.227590
IL27RA	0.291397	0.224237
MAP3K3	0.305085	0.222530
RAB5C	0.734371	0.222530
PLEKHA1	-0.254226	0.222353
CCR5	0.416724	0.222353
MME	0.464054	0.222353
NKG7	0.243504	0.219997
HMOX1	0.915392	0.219997
STT3B	0.447614	0.219045
PECAM1	-0.358050	0.218711
CREBBP	0.355989	0.218711
ATF6	0.734567	0.212475
MAVS	0.363596	0.211220
AP1G1	0.247776	0.207305
DNAJC10	0.231844	0.200503
MVP	0.600109	0.198610
IKBKB	0.393123	0.198249
TLR8	0.178171	0.194519
NT5E	-0.202247	0.193097
TLN1	0.303601	0.192013
GNLY	0.458186	0.192013
SOCS1	0.385518	0.190285
IFNA4/7/10/17/21	0.355351	0.179788
AP1M1	0.614813	0.168272
CD4	0.353194	0.167901
FCGRT	0.303258	0.163003
ATF4	0.223523	0.161939
RELA	0.256223	0.161939
C1QBP	0.291248	0.161939
EIF2AK2	0.298664	0.161939
STAT2	0.319293	0.161939
MAFB	0.330286	0.161939
IL5	0.334118	0.161939
TCN2	0.355079	0.161939
CXCL12	0.423774	0.161939
CBLB	0.566807	0.161939
CXCL14	0.661379	0.161939
RIPK1	0.204270	0.161023
JAK1	0.223479	0.161023
SELENOS	0.252049	0.161023
SUGT1	0.295530	0.161023
VAMP3	0.308312	0.161023
IFI16	0.598310	0.161023
JUN	-0.306659	0.159106

MAPK8	0.240930	0.156491
IGFBP7	-0.357413	0.153463
MT2A	0.295120	0.151449
TGFB3	-0.181826	0.148282
MAP3K7	0.157536	0.148282
CTSW	0.178695	0.148282
LAT	0.274716	0.148282
THBS1	0.279162	0.148282
TGFBR2	-0.228668	0.146355
PELI1	0.232469	0.146355
PRKCA	0.308498	0.144402
SIGIRR	0.153465	0.140288
SCARB2	-0.223734	0.139532
PPIA	0.158120	0.136965
AHR	0.257371	0.136965
GZMH	0.276232	0.134230
IL32	0.314617	0.133437
TGFB1	0.178047	0.132554
PRCP	-0.267280	0.130992
KLRK1	-0.147830	0.130992
GNS	0.271042	0.130992
CCL2	-0.305440	0.125491
LILRA3	-0.195543	0.125491
ATF2	0.232391	0.125491
IL6ST	-0.206135	0.124020
BCR	-0.119962	0.124020
AKT3	0.232252	0.123446
ITGB2	0.257152	0.122532
TRAM1	0.177212	0.120079
THOP1	0.247093	0.119016
MYC	-0.247634	0.118649
BNIP3	0.230764	0.118649
HPGD	0.258284	0.118649
YWHAQ	0.178993	0.113802
DDOST	0.193322	0.113802
PDHB	0.383851	0.113382
MAP2K4	0.192008	0.108914
MDFIC	0.422664	0.108296
APP	-0.196562	0.101825
TAB1	0.167877	0.101825
ACKR4	0.202338	0.101825
LDHB	0.200223	0.100943
MAF	0.190882	0.097582
CTSA	0.201878	0.093326
PSMB8	0.086514	0.091401

PLCG1	-0.198860	0.090641
CD68	0.182029	0.090641
TXNIP	0.174632	0.089226
FYN	0.335892	0.085858
NFATC2	0.136659	0.079781
CD45RA	-0.160711	0.075536
SMAD3	-0.167103	0.072131
CTSZ	0.175552	0.068345
TRAF2	0.137053	0.068150
RPS6KA3	0.153230	0.068150
KIR2DL1	0.170874	0.068150
CCL5	0.133908	0.067929
HCST	-0.150770	0.062224
CD84	0.160489	0.062224
CCL21	0.299773	0.062224
CGAS	-0.073540	0.061228
DDAH2	0.115829	0.061228
LEF1	0.164811	0.061228
NEO1	0.095101	0.057189
CTSL	0.164288	0.053030
ATG4A	-0.098603	0.049614
TBX21	-0.094726	0.049614
NPC2	0.089659	0.049614
CALM1	-0.079090	0.049380
BST2	0.111507	0.049380
EVL	0.124474	0.049380
SEM1	0.130188	0.049380
ETS1	0.111870	0.046486
ENTPD1	0.099505	0.041833
CD276	-0.118322	0.040552
RACK1	0.062572	0.039188
CCL28	0.064022	0.039157
HLA-E	0.166831	0.039157
NFAT5	-0.073755	0.036733
SORT1	-0.090853	0.032586
GLB1	0.058390	0.032228
PANX1	0.085878	0.032228
NFATC1	-0.068301	0.027896
EIF3F	0.047551	0.026106
LAMP1	0.037659	0.023489
APEX1	0.048715	0.023489
MTOR	0.051530	0.023489
TRIM56	0.035493	0.016322
LANCL1	-0.046525	0.015664
IFNA6	0.049062	0.015664

TIMP2	-0.046464	0.014810
CD81	-0.034983	0.011072
SOD1	0.023054	0.007469
CD59	0.024188	0.007469
MS4A1	0.029206	0.007469
HSP90AB1	-0.017825	0.006801
HAVCR2	0.017943	0.005348
FOXO1	-0.015914	0.004258
NRAS	-0.017519	0.001959
VCAM1	-0.013704	0.001959
TPSAB1/B2	-0.007956	0.001959
LGALS3	-0.005081	0.001959
STING1	-0.004722	0.001959
ACVR1	0.006871	0.001959
PRKCSH	0.012811	0.001959
UBE2N	-0.001013	0.000397
CDK4	-0.000649	0.000397
ACE	0	0
ACKR2	0	0
ACSL3	0	0
ADGRG3	0	0
ADRA	0	0
AGT	0	0
AICDA	0	0
ALOX12	0	0
ALOX15	0	0
BATF	0	0
BDKRB1	0	0
BDKRB2	0	0
BLK	0	0
BPI	0	0
C2	0	0
C5	0	0
CARD11	0	0
CARD16	0	0
CASP5	0	0
CCL1	0	0
CCL13	0	0
CCL15	0	0
CCL16	0	0
CCL17	0	0
CCL19	0	0
CCL20	0	0
CCL22	0	0
CCL23	0	0

CCL25	0	0
CCL26	0	0
CCL27	0	0
CCL7	0	0
CCL8	0	0
CCR10	0	0
CCR4	0	0
CCR6	0	0
CCR7	0	0
CCR8	0	0
CCR9	0	0
CCRL2	0	0
CD19	0	0
CD1E	0	0
CD2	0	0
CD209	0	0
CD244	0	0
CD247	0	0
CD38	0	0
CD40	0	0
CD40LG	0	0
CD6	0	0
CD70	0	0
CD79A	0	0
CD79B	0	0
CD80	0	0
CD8A	0	0
CD8B	0	0
CHUK	0	0
CLFA	0	0
CLFB	0	0
CRP	0	0
CSF2	0	0
CSF3	0	0
CSGB	0	0
CTSG	0	0
CUPA1	0	0
CUPA4	0	0
CX3CL1	0	0
CXCL10	0	0
CXCL11	0	0
CXCL13	0	0
CXCL17	0	0
CXCL6	0	0
CXCR3	0	0

CXCR5	0	0
CXCR6	0	0
CYP2E1	0	0
DEFB103A/B	0	0
DHX58	0	0
EBI3	0	0
ELANE	0	0
EOMES	0	0
EPHX2	0	0
FAM30A	0	0
FASLG	0	0
FBXO6	0	0
FCRL2	0	0
FCRL4	0	0
FLGM	0	0
FLIA	0	0
FNBA	0	0
FNBB	0	0
GAB2	0	0
GATA3	0	0
GBP1	0	0
GZMB	0	0
HAMP	0	0
HDC	0	0
HLA-DOB	0	0
HLA-DQB1	0	0
HSD11B1	0	0
ICAA	0	0
ICAB	0	0
ICAC	0	0
ICOS	0	0
ICOSLG	0	0
IDO1	0	0
IFNA1/13	0	0
IFNA14/16	0	0
IFNA2	0	0
IFNA5	0	0
IFNB1	0	0
IFNG	0	0
IFNK	0	0
IFNL1	0	0
IFNL2/3	0	0
IFNL4	0	0
IFNLR1	0	0
IFNW1	0	0

IGHD	0	0
IGHE	0	0
IKBKE	0	0
IL10	0	0
IL11	0	0
IL11RA	0	0
IL12A	0	0
IL12B	0	0
IL12RB1	0	0
IL12RB2	0	0
IL13	0	0
IL13RA2	0	0
IL15	0	0
IL15RA	0	0
IL17A	0	0
IL17B	0	0
IL17C	0	0
IL17D	0	0
IL17F	0	0
IL17RB	0	0
IL17RC	0	0
IL17RD	0	0
IL17RE	0	0
IL18BP	0	0
IL18RAP	0	0
IL19	0	0
IL1A	0	0
IL1F10	0	0
IL1RAPL1	0	0
IL1RAPL2	0	0
IL1RL1	0	0
IL1RL2	0	0
IL20	0	0
IL20RA	0	0
IL20RB	0	0
IL21	0	0
IL21R	0	0
IL22	0	0
IL22RA1	0	0
IL22RA2	0	0
IL23A	0	0
IL24	0	0
IL25	0	0
IL26	0	0
IL27	0	0

IL2RA	0	0
IL3	0	0
IL31	0	0
IL31RA	0	0
IL34	0	0
IL36A	0	0
IL36B	0	0
IL36G	0	0
IL36RN	0	0
IL37	0	0
IL3RA	0	0
IL4	0	0
IL5RA	0	0
IL7	0	0
IL9	0	0
IL9R	0	0
IRF3	0	0
ITGAE	0	0
ITGB7	0	0
ITK	0	0
ITLN1	0	0
KIR2DL3	0	0
KIR3DL1/2	0	0
KLRD1	0	0
LAG3	0	0
LAMP3	0	0
LCK	0	0
LECA	0	0
LECB	0	0
LIF	0	0
LTC4S	0	0
MAP3K5	0	0
MKNK1	0	0
MS4A2	0	0
MX1	0	0
NCR1	0	0
NCR3	0	0
NFATC4	0	0
NLRC5	0	0
NOS2	0	0
NTNG2	0	0
OAS3	0	0
OASL	0	0
P2RX7	0	0
PDCD1	0	0

PDCD1LG2	0	0
PELI2	0	0
PIK3R3	0	0
PIK3R6	0	0
PLG	0	0
PNOC	0	0
PRF1	0	0
RASGRP1	0	0
RELB	0	0
RGMA	0	0
RPS6KB1	0	0
RUNX3	0	0
SH2D1A	0	0
SPIB	0	0
STAT4	0	0
TCF7	0	0
TCL1A	0	0
TGFB2	0	0
TIFA	0	0
TIGIT	0	0
TLR3	0	0
TMEM140	0	0
TMPRSS2	0	0
TNF	0	0
TNFRSF17	0	0
TNFRSF18	0	0
TNFRSF4	0	0
TNFRSF9	0	0
TNFSF18	0	0
TNFSF4	0	0
TNFSF9	0	0
TRAT1	0	0
TRIM5	0	0
TRIM6	0	0
TXK	0	0
ULK2	0	0
XCL1/2	0	0

Table S5: Comparison between *Enterobacter cloacae* infected FRI patients (n=2) vs a non-infected control group. P-values were adjusted using the Benjamini–Hochberg false discovery rate (FDR). Significant genes are indicated in bold. (-log (p-adjusted > 1.3)).

Symbol	Log2FoldChange	-log(p-adjusted)
GK	5.852332	2.569079
PFKFB3	5.134722	2.569079
CXCL1	6.551332	2.375681
PTGS2	6.020618	2.375681
PTK2B	3.524167	2.375681
SOD2	5.226286	2.375681
ADORA2A	3.891624	2.284975
CCL4/L1/L2	5.384073	2.284975
PLAU	6.035756	2.155790
SIGLEC5	4.919703	2.155790
CYSTM1	3.266702	1.990623
ACSL1	5.246353	1.910593
CXCL2	4.728634	1.910593
PLEK	5.974955	1.910593
IL1B	7.972699	1.659005
NLRP3	4.114464	1.655667
LCP2	4.889427	1.644005
NAMPT	4.363510	1.582386
NFKB1	3.573735	1.582386
PLAUR	4.717700	1.582386
SLC2A3	4.717311	1.582386
IRAK3	3.799008	1.566781
GADD45B	3.847448	1.496726
MAPK13	2.656545	1.496726
IL4R	2.862335	1.454476
PRKCD	3.615085	1.447876
CD44	2.998148	1.444550
ATG7	4.063088	1.426447
CEBPB	2.347253	1.415358
LIMK2	4.115153	1.415358
NOD2	3.710753	1.415358
GBP2	2.498812	1.398447
IL2RG	2.873825	1.398447
CCL24	-3.521490	1.364768
BCL3	3.771135	1.354771
FPR2	4.063945	1.354771
GLA	1.807355	1.354771
PIK3R5	3.970456	1.354771
NFKB2	3.632857	1.332859
C3AR1	3.853112	1.302933

KDM6B	3.695245	1.291056
IFNGR2	3.194293	1.285907
CSF2RB	3.021324	1.277509
FCAR	3.827213	1.277509
GPX7	-3.105630	1.277509
CD274	2.258016	1.241187
CEACAM3	2.694587	1.241187
IL18R1	3.647750	1.241187
TYK2	1.686842	1.241187
CD45RB	2.136817	1.200743
CXCL3	3.378080	1.197366
CXCL8	6.183981	1.197366
IFITM1	2.584063	1.197366
MEFV	3.659256	1.197366
SERPINA1	3.945264	1.197366
TRAF3	3.572890	1.197366
ERN1	3.327949	1.164613
IFITM2	3.028447	1.151581
CR1	3.725182	1.134326
TRIM25	3.173004	1.130040
ALOX5	3.058172	1.116008
ALOX5AP	3.861375	1.116008
CD22	2.894524	1.116008
HCK	2.587692	1.116008
TLR2	3.143160	1.106425
ITGAX	3.558798	1.088490
VEGFA	4.040299	1.088490
LYN	3.528676	1.060285
NCF2	3.621027	1.054533
RAB7A	2.037160	1.044322
DDIT3	3.731326	1.039625
CCL3/L1/L3	5.918905	1.037236
CD14	2.540433	1.037236
IL18	1.998539	1.034044
BCL6	3.317171	1.030398
PAK1	3.182282	1.030398
MARCKS	2.839842	1.021287
SLC11A1	3.607730	1.021287
ATP6V1B2	2.860369	0.984610
GBP5	3.210377	0.984610
LCP1	3.338896	0.984610
OSM	5.675348	0.984610
DYSF	2.696411	0.961465
IFITM3	2.097763	0.961465
LILRA5	2.990316	0.922573

LRG1	3.448438	0.922573
REL	2.480975	0.922573
RHOG	2.225553	0.922573
STAT3	2.724761	0.922573
FPR1	3.382154	0.910105
IL6R	2.681066	0.910105
TANK	2.768499	0.909266
CASP4	3.057607	0.906602
CSF2RA	2.121587	0.906602
FGR	3.113316	0.906602
RAC2	3.045633	0.885430
CXCL5	3.226870	0.881966
S100A12	3.244087	0.873524
LITAF	2.665061	0.863553
NOX1	-2.820680	0.863195
IL10RA	1.984010	0.839561
CCL18	6.520288	0.828214
IL1RN	5.880369	0.821782
NFE2L2	2.432457	0.821782
XBP1	2.296189	0.821782
IL1R2	2.969684	0.810709
SOCS3	3.126677	0.796384
CASP1	2.351179	0.775780
CCR2	2.314959	0.775780
CFLAR	2.097788	0.775780
FCGR2A	2.684100	0.775780
PTGER4	1.894021	0.775780
NCF1	2.594762	0.753513
TBK1	2.567779	0.753513
CD69	2.797177	0.742247
CSF3R	3.290336	0.742247
MAP2K3	2.295213	0.739928
TLR6	2.303124	0.733312
NT5E	-1.255470	0.732122
STAT5B	1.553936	0.699840
IL1RAP	2.582063	0.692358
RASGRP4	2.083565	0.692358
C5AR1	2.702398	0.670947
IRF4	-1.897190	0.669601
MAP1LC3A	2.256608	0.669601
NCF4	2.573485	0.669601
TOLLIP	1.937825	0.669601
DIABLO	1.738516	0.663974
JAK3	2.525240	0.663974
JAK2	2.264213	0.647915

ARRB2	2.677558	0.645067
CD3E	2.014680	0.643401
AIM2	1.535102	0.635854
NGLY1	1.733725	0.632345
ZBP1	2.436517	0.630233
LTA4H	1.804835	0.627870
TRIM33	1.171943	0.627870
LILRA6	1.732221	0.627466
IGHM	3.676407	0.612226
IL10RB	2.178765	0.606386
XCR1	-1.980240	0.599473
MAPK14	1.432035	0.598610
NLRC4	1.824150	0.585685
IL7R	1.692462	0.576465
JUNB	2.036743	0.576465
GCA	2.333388	0.576248
HSP90B1	1.727717	0.576248
PLAT	-2.139980	0.576248
RIPK2	1.702123	0.576248
SYK	1.704080	0.576248
TNFSF13B	2.278730	0.576248
AIF1	1.734370	0.575485
CD45R0	2.420929	0.575485
KRAS	1.907466	0.575485
STAT6	1.432870	0.575485
TLR8	0.891568	0.557151
CARD17	2.041986	0.542618
CTLA4	1.533061	0.542618
IGHA	3.572260	0.542618
IL17RA	1.474007	0.542618
MCL1	2.095318	0.542618
SIRPA	2.154328	0.528089
GUCY1A1	1.732393	0.521574
F5	1.782409	0.520608
MT2A	1.805850	0.520608
TLR1	1.515654	0.520608
CD86	2.086232	0.518185
MLKL	1.479122	0.518185
PLCG2	1.835924	0.512114
ACSL4	1.753916	0.503964
TCIRG1	1.562821	0.503964
ALPL	1.952216	0.501230
IL6	2.365212	0.486139
PTPRC	1.935748	0.486139
CCR1	2.337117	0.482699

GBA	1.890683	0.482699
IFIH1	1.474550	0.482699
IRF7	1.817697	0.482699
RPS6KA1	1.934422	0.482699
ITPR3	-0.998590	0.477758
C3	3.564747	0.475477
TGFB3	-1.107340	0.473524
CRK	1.383856	0.469289
PLIN4	1.706072	0.466293
TPP1	1.382242	0.464264
FCGR1A/B	1.973365	0.464180
ATP6V0D1	1.738642	0.450527
CXCL16	2.051165	0.450527
HLA-DQA	1.813329	0.447098
ITGAM	1.893770	0.433400
HK3	1.343247	0.432461
MAP3K8	1.724413	0.432461
HLA-B	1.377553	0.427326
AP1G1	1.027221	0.425331
CBL	2.972373	0.425331
CCL14	-1.892170	0.425331
HLA-C	1.317993	0.425331
HMOX1	2.288845	0.425331
IKBKG	1.253119	0.425331
MYD88	1.643379	0.425331
SPI1	1.892721	0.425331
HLA-DPA1	1.516544	0.419303
TRAF6	1.370915	0.419303
HLA-DRA	1.505829	0.417428
LILRB2	1.757169	0.417428
SP100	3.012941	0.417428
TAP1	1.316064	0.417428
TLR4	1.492540	0.417428
IGHG	4.152939	0.415866
IL1R1	1.447545	0.415866
RELA	1.214466	0.415866
LAT2	1.845721	0.414424
JAK1	1.091702	0.407268
FOXP3	-1.362570	0.407265
IFNAR1	1.260017	0.407265
IRAK1	1.700094	0.407265
MGAM	1.772890	0.407265
PLEKHA1	-0.974770	0.407265
PRDM1	1.295790	0.407265
SMAD5	1.227931	0.407265

STAT1	1.714361	0.407265
TAB2	2.913236	0.407265
CXCR4	1.793168	0.401781
HLA-A	1.365821	0.400800
CCR5	1.532248	0.397516
TLN1	1.238300	0.395023
HLA-DRB	1.277301	0.392947
NLRP1	0.797949	0.390452
ATG3	2.940973	0.383919
IRAK4	0.818611	0.383574
TBXAS1	1.876546	0.383574
RNF135	0.613240	0.380029
TYROBP	1.752987	0.371936
FCGR3A/B	1.478955	0.368503
LTB	1.546230	0.363094
DDX58	1.402513	0.361669
APBB1IP	2.654623	0.350194
FAS	2.243246	0.350194
PIK3CG	1.438710	0.350194
WAS	1.715432	0.350194
PARP9	1.313815	0.347647
HLA-DPB1	1.654777	0.347222
RNF114	1.298891	0.347222
SP1	1.145173	0.347222
LTBR	2.238858	0.341606
PXN	1.167545	0.341606
ADAR	2.309009	0.336167
STAT5A	2.677601	0.335262
PIK3CD	1.411509	0.331080
PSMB9	1.173302	0.331080
SELL	1.626344	0.331080
TRIM22	1.377671	0.329163
UBA52	1.140043	0.329163
LRRK2	1.455340	0.326630
DEFA1	2.566049	0.314890
CTSS	1.398690	0.312608
CD163	1.453718	0.306834
FURIN	1.297069	0.306834
VWF	-1.225290	0.306834
IFNAR2	1.414383	0.305606
PIK3R4	1.276840	0.297057
CAP1	1.192460	0.296858
CXCL9	1.413512	0.296858
IKBKB	2.095482	0.296858
LAMP2	1.368005	0.296858

MIF	0.750208	0.296858
PSEN1	2.260936	0.296858
ALPK1	1.283383	0.295889
TRIM21	1.886703	0.295889
AP1S2	2.333575	0.294781
LTF	2.380953	0.294781
MARCO	1.651342	0.294781
TXN	1.087412	0.294781
PIK3CB	1.311397	0.291257
GNS	1.274136	0.288484
FOS	1.525925	0.288454
BCR	-0.599870	0.284242
PSMB10	1.082633	0.283591
PTPN6	1.359316	0.283591
JAML	1.468921	0.281983
DNAJA2	1.164512	0.280726
ANPEP	1.230272	0.279589
RNASEL	1.051024	0.273453
ACOX1	1.029489	0.264607
MAPKAPK2	1.055766	0.264607
RIPK3	0.738193	0.264607
BECN1	-0.774810	0.263252
CXCR2	1.076726	0.263252
KPNB1	2.224598	0.263252
LCN2	1.537266	0.263252
NOTCH1	1.198145	0.263252
RAB31	2.462481	0.263252
RAB5C	2.111440	0.263252
RBCK1	2.031820	0.263252
BCL2L1	1.439155	0.261016
IRF9	1.256486	0.258757
TLR5	1.131977	0.258757
PELI1	0.919428	0.257599
TAP2	0.605211	0.256476
HSP90AA1	0.791409	0.255791
IRF1	1.067838	0.255791
MAPK1	0.875128	0.255791
MAFB	1.137612	0.252053
AKT3	1.034174	0.251969
CD276	-1.384380	0.251969
CXCR1	1.270137	0.251969
MAP2K7	1.920681	0.251969
MS4A7	1.389264	0.251969
RBPJ	0.964896	0.251969
TNFRSF1A	2.174226	0.251969

IL2	1.386512	0.250295
ATM	2.250172	0.249221
HLA-DMB	1.225624	0.249221
IFI35	1.207893	0.249221
PTGER2	0.749593	0.249221
HLA-DMA	1.277464	0.242479
AKT2	1.861286	0.238871
HLX	0.883437	0.238871
IFIT2	1.002788	0.238871
TNFRSF10B	1.212742	0.238871
RSAD2	0.804352	0.238157
ATF6	1.974552	0.238110
IL13RA1	0.788290	0.238110
CD68	0.979750	0.233434
ATF2	0.954413	0.232733
MRC1	1.049511	0.228582
PSAP	0.991221	0.228582
SMAD4	0.734858	0.228449
IL16	0.861803	0.227188
KLRB1	1.208560	0.227188
NEU1	0.807355	0.227188
NFATC2	-0.815450	0.227188
ATG12	0.726715	0.216625
MME	1.121886	0.216625
RAF1	1.110810	0.216625
TRAC	1.146940	0.216625
VRK3	1.381476	0.213882
SELE	1.076368	0.212164
VSIR	1.136201	0.206870
GBP4	0.618053	0.204167
MVP	1.542018	0.204167
STRAP	1.733353	0.204167
UBE2L6	0.862147	0.204167
CREBBP	0.843360	0.203340
NEO1	-0.704460	0.202951
DERL1	1.816816	0.202008
GSK3B	0.845927	0.202008
ACKR4	-0.900990	0.197349
ATF4	0.636474	0.197349
CCL28	-0.644340	0.197349
IFI16	1.774646	0.197349
PSTPIP1	0.727628	0.197349
OS9	0.670597	0.197306
PSMB8	0.411172	0.197202
CSF1	0.864959	0.196145

MAP3K1	0.916702	0.196145
PECAM1	-0.789980	0.196145
PIK3CA	0.751978	0.196145
RB1CC1	0.784860	0.196145
SAMHD1	0.826984	0.196145
VAMP3	0.875151	0.193799
CD27	0.973649	0.192691
CTSW	-0.528470	0.191751
NAE1	-0.667330	0.191751
LAMP1	0.542777	0.191034
MSRA	1.318725	0.191034
NFAT5	0.741266	0.189915
ATP6AP2	1.456111	0.189540
IL33	-1.061820	0.189540
PYCARD	0.794828	0.187235
APOBEC3G	0.936341	0.183570
CXCL12	1.107446	0.183570
HPGD	0.923548	0.183570
IGFBP7	-0.996340	0.183570
STAT2	0.820141	0.183570
DTX3L	0.567442	0.179363
IFNA6	-0.908590	0.177765
ITGB2	0.859061	0.177744
MAP2K4	0.717053	0.177744
SSR1	0.756536	0.170667
DDX5	0.663395	0.169305
CASP8	0.793094	0.168078
CBFB	1.431993	0.168078
CD4	0.822896	0.168078
HLA-E	0.743727	0.168078
ITGAL	0.861493	0.168078
MAP3K3	0.582361	0.168078
MAPK9	1.103047	0.168078
NFATC3	1.348978	0.168078
SOCS1	0.835369	0.168078
TRIM56	-0.586920	0.168078
PTPN4	0.593230	0.166240
CSF1R	0.820775	0.163322
CASP10	0.749408	0.161824
ULK1	0.369775	0.161824
LGALS3	0.687478	0.152327
MS4A1	0.798291	0.152327
MAVS	1.067007	0.149186
NDUFS8	1.350269	0.149186
AKT1	1.276145	0.145587

SMAD3	-0.746680	0.145587
APOL6	0.836423	0.141802
CBLB	1.194079	0.141802
CCR3	0.539875	0.141802
CD36	-0.815620	0.141802
LILRA3	-0.540110	0.141802
PIK3C3	-0.338770	0.141802
SIGIRR	-0.377860	0.138834
FOXO1	-0.623170	0.136517
IFI27	-0.707220	0.136517
MAP2K2	0.527618	0.136517
RNF31	0.618129	0.136517
TBX21	-0.557640	0.136517
YWHAQ	-0.527630	0.136517
ICAM3	0.513695	0.136000
ADGRE5	0.769506	0.135907
AP1M1	1.197275	0.135907
MDFIC	1.289310	0.135760
CUL1	0.519248	0.134856
THBS1	0.603030	0.128408
IFIT1	-0.627050	0.128130
OAS2	0.672705	0.125976
CD84	0.694792	0.123717
CTSA	0.627337	0.122748
PLCG1	-0.636140	0.122748
IL32	0.689959	0.112430
ZAP70	0.479835	0.112430
IL27RA	0.401363	0.111048
GZMA	0.627780	0.109268
STT3B	0.634804	0.108926
BNIP3	0.547488	0.106762
NKG7	0.335830	0.106762
PDHB	0.969711	0.106762
ACKR3	0.648365	0.106586
CD28	0.584963	0.106586
BST2	0.552381	0.105505
MAP3K7	0.295641	0.105505
CPA3	0.609818	0.104250
GSTM4	0.467505	0.103659
GUCY1B1	0.637430	0.103659
IL2RB	0.534829	0.102204
CD45RA	-0.512790	0.101659
AHR	0.856294	0.101270
CGAS	0.280660	0.101270
CTSL	0.684645	0.101270

PARP1	0.832971	0.101270
PRKCA	0.559427	0.101270
PRKCQ	0.336563	0.101270
TXNIP	0.480315	0.101270
KLRC1	0.472565	0.100162
DDAH2	0.424351	0.099861
HCST	0.530656	0.099861
PRCP	-0.503060	0.099846
TAB1	-0.389950	0.092512
KLRK1	-0.270140	0.091766
TCN2	-0.520330	0.090125
BCL2	0.452398	0.089356
DNAJC10	0.278976	0.089356
LEF1	0.557032	0.089356
PRKCSH	0.678719	0.089356
SEM1	-0.532220	0.089356
VCAM1	0.541114	0.089356
TNFRSF25	-0.413750	0.087282
CD3G	-0.469090	0.086126
EIF2AK3	0.334534	0.086126
MTOR	0.374396	0.086126
TLR9	-0.296070	0.086126
MAPK8	0.336049	0.085370
CCL21	-0.933260	0.085109
APP	-0.376890	0.082820
CALM1	-0.296850	0.082820
CXCL14	-0.833770	0.082820
IL23R	0.403839	0.082820
RPS6KA3	0.409047	0.082820
SCARB2	-0.331130	0.082820
SUGT1	0.393928	0.082820
TRAM1	0.297642	0.082820
CD3D	0.399270	0.079888
LANCL1	-0.415040	0.079888
IFNA8	0.415037	0.079348
HERC5	0.407424	0.079094
HSP90AB1	0.292940	0.079094
MS4A4A	0.521464	0.078580
DDOST	0.311847	0.077859
FCGRT	0.343359	0.077859
NFATC1	0.356144	0.077859
TPSAB1/B2	0.439984	0.077859
EIF3F	-0.259390	0.074552
NPC2	0.275171	0.073826
MAF	-0.322340	0.068435

SORT1	0.383889	0.068435
XAF1	0.374396	0.065131
CD59	-0.288730	0.065069
FYN	0.606639	0.065069
SOD1	-0.292850	0.065069
CTSZ	0.353362	0.063546
IFNA4/7/10/17/21	0.301248	0.058831
EIF2AK2	0.268196	0.056689
TIMP2	-0.319530	0.056689
ISG15	-0.330860	0.053503
UBE2N	0.228233	0.051720
PANX1	0.294447	0.050976
CX3CR1	0.210051	0.049180
HMGB1	-0.134820	0.049180
LAT	-0.239120	0.049180
ENTPD1	0.245131	0.045795
C1QBP	0.207096	0.042376
ETS1	0.241451	0.042376
IFI6	-0.272570	0.042221
ACVR1	-0.231170	0.041909
ATG10	0.191696	0.039736
CASP3	0.285928	0.039736
CDH1	-0.162990	0.039736
MYC	-0.213760	0.039736
PPIA	-0.120390	0.039736
RIPK1	0.130847	0.039736
IFI44	0.200546	0.038468
GZMH	-0.198100	0.037424
ATG4A	0.157925	0.036378
ATG13	-0.081940	0.032992
SELENOS	0.124545	0.026966
CCL11	-0.136690	0.025303
EGLN1	0.114333	0.025303
EVL	-0.149420	0.025303
GNLY	0.176700	0.025303
LDHB	0.135108	0.025303
TRAF2	0.130332	0.025303
TGFBR2	-0.097590	0.022230
TLR7	-0.059380	0.021952
KIR2DL1	0.125681	0.019558
CCL2	-0.130890	0.019258
NRAS	-0.100690	0.018773
CCL5	0.081965	0.015397
CDK4	0.073672	0.008655
HAVCR2	-0.059480	0.008457

APEX1	-0.045800	0.005994
CCNC	-0.031770	0.004317
CD81	0.027710	0.004317
DEFA4	-0.028260	0.004317
GLB1	0.023301	0.004317
IL6ST	0.030232	0.004317
JUN	0.025460	0.004317
RACK1	0.019919	0.004317
TGFB1	-0.022420	0.004317
TNFSF10	0.034626	0.004317
WIPI1	0.038863	0.004317
OAS1	-0.011730	0.002542
STING1	-0.014180	0.002542
IFIT3	-0.009340	0.002075
IL5	-0.009340	0.002075
ACE	0	0
ACKR2	0	0
ACSL3	0	0
ADGRG3	0	0
ADRA	0	0
AGT	0	0
AICDA	0	0
ALOX12	0	0
ALOX15	0	0
BATF	0	0
BDKRB1	0	0
BDKRB2	0	0
BLK	0	0
BPI	0	0
C2	0	0
C5	0	0
CARD11	0	0
CARD16	0	0
CASP5	0	0
CCL1	0	0
CCL13	0	0
CCL15	0	0
CCL16	0	0
CCL17	0	0
CCL19	0	0
CCL20	0	0
CCL22	0	0
CCL23	0	0
CCL25	0	0
CCL26	0	0

CCL27	0	0
CCL7	0	0
CCL8	0	0
CCR10	0	0
CCR4	0	0
CCR6	0	0
CCR7	0	0
CCR8	0	0
CCR9	0	0
CCRL2	0	0
CD19	0	0
CD1E	0	0
CD2	0	0
CD209	0	0
CD244	0	0
CD247	0	0
CD38	0	0
CD40	0	0
CD40LG	0	0
CD6	0	0
CD70	0	0
CD79A	0	0
CD79B	0	0
CD80	0	0
CD8A	0	0
CD8B	0	0
CHUK	0	0
CLFA	0	0
CLFB	0	0
CRP	0	0
CSF2	0	0
CSF3	0	0
CSGB	0	0
CTSG	0	0
CUPA1	0	0
CUPA4	0	0
CX3CL1	0	0
CXCL10	0	0
CXCL11	0	0
CXCL13	0	0
CXCL17	0	0
CXCL6	0	0
CXCR3	0	0
CXCR5	0	0
CXCR6	0	0

CYP2E1	0	0
DEFB103A/B	0	0
DHX58	0	0
EBI3	0	0
ELANE	0	0
EOMES	0	0
EPHX2	0	0
FAM30A	0	0
FASLG	0	0
FBXO6	0	0
FCRL2	0	0
FCRL4	0	0
FLGM	0	0
FLIA	0	0
FNBA	0	0
FNBB	0	0
GAB2	0	0
GATA3	0	0
GBP1	0	0
GZMB	0	0
HAMP	0	0
HDC	0	0
HLA-DOB	0	0
HLA-DQB1	0	0
HSD11B1	0	0
ICAA	0	0
ICAB	0	0
ICAC	0	0
ICOS	0	0
ICOSLG	0	0
IDO1	0	0
IFNA1/13	0	0
IFNA14/16	0	0
IFNA2	0	0
IFNA5	0	0
IFNB1	0	0
IFNG	0	0
IFNK	0	0
IFNL1	0	0
IFNL2/3	0	0
IFNL4	0	0
IFNLR1	0	0
IFNW1	0	0
IGHD	0	0
IGHE	0	0

IKBKE	0	0
IL10	0	0
IL11	0	0
IL11RA	0	0
IL12A	0	0
IL12B	0	0
IL12RB1	0	0
IL12RB2	0	0
IL13	0	0
IL13RA2	0	0
IL15	0	0
IL15RA	0	0
IL17A	0	0
IL17B	0	0
IL17C	0	0
IL17D	0	0
IL17F	0	0
IL17RB	0	0
IL17RC	0	0
IL17RD	0	0
IL17RE	0	0
IL18BP	0	0
IL18RAP	0	0
IL19	0	0
IL1A	0	0
IL1F10	0	0
IL1RAPL1	0	0
IL1RAPL2	0	0
IL1RL1	0	0
IL1RL2	0	0
IL20	0	0
IL20RA	0	0
IL20RB	0	0
IL21	0	0
IL21R	0	0
IL22	0	0
IL22RA1	0	0
IL22RA2	0	0
IL23A	0	0
IL24	0	0
IL25	0	0
IL26	0	0
IL27	0	0
IL2RA	0	0
IL3	0	0

IL31	0	0
IL31RA	0	0
IL34	0	0
IL36A	0	0
IL36B	0	0
IL36G	0	0
IL36RN	0	0
IL37	0	0
IL3RA	0	0
IL4	0	0
IL5RA	0	0
IL7	0	0
IL9	0	0
IL9R	0	0
IRF3	0	0
ITGAE	0	0
ITGB7	0	0
ITK	0	0
ITLN1	0	0
KIR2DL3	0	0
KIR3DL1/2	0	0
KLRD1	0	0
LAG3	0	0
LAMP3	0	0
LCK	0	0
LECA	0	0
LECB	0	0
LIF	0	0
LTC4S	0	0
MAP3K5	0	0
MKNK1	0	0
MS4A2	0	0
MX1	0	0
NCR1	0	0
NCR3	0	0
NFATC4	0	0
NLRC5	0	0
NOS2	0	0
NTNG2	0	0
OAS3	0	0
OASL	0	0
P2RX7	0	0
PDCD1	0	0
PDCD1LG2	0	0
PELI2	0	0

PIK3R3	0	0
PIK3R6	0	0
PLG	0	0
PNOC	0	0
PRF1	0	0
RASGRP1	0	0
RELB	0	0
RGMA	0	0
RPS6KB1	0	0
RUNX3	0	0
SH2D1A	0	0
SPIB	0	0
STAT4	0	0
TCF7	0	0
TCL1A	0	0
TGFB2	0	0
THOP1	0	0
TIFA	0	0
TIGIT	0	0
TLR3	0	0
TMEM140	0	0
TMPRSS2	0	0
TNF	0	0
TNFRSF17	0	0
TNFRSF18	0	0
TNFRSF4	0	0
TNFRSF9	0	0
TNFSF18	0	0
TNFSF4	0	0
TNFSF9	0	0
TRAT1	0	0
TRIM5	0	0
TRIM6	0	0
TXK	0	0
ULK2	0	0
XCL1/2	0	0

Table S6: Comparison between *Staphylococcus aureus* infected FRI patients (n=6) vs a non-infected control group (n=16). Significant genes are indicated in bold (-log (p-value) > 1.3)). No DEGs could be identified with an adjusted p-value < 0.05, uncorrected p-values of < 0.05 were used.

Symbol	Log2FoldChange	-log(p-value)
CASP3	3.450025	3.419203
RSAD2	1.891815	3.315993
XBP1	2.106308	2.632428
WIPI1	1.934526	2.430697
PLAT	-2.257820	2.330407
NOD2	2.266560	2.218357
LCP2	2.661651	2.136316
ISG15	2.122857	1.983474
CDH1	1.434966	1.897020
SLC2A3	2.285209	1.693400
VEGFA	2.423448	1.668264
CD45RB	1.177904	1.643046
IFIT1	1.631104	1.626481
IFIT2	1.570316	1.615740
CD36	-1.976040	1.594596
IFI6	1.842909	1.559301
IRF7	1.595304	1.521838
HERC5	1.663764	1.506212
ZBP1	1.807355	1.478551
PYCARD	1.353456	1.394843
IFI27	1.511579	1.362935
IRF9	1.554322	1.350727
IFIH1	1.178385	1.309462
IFIT3	1.413874	1.301020
GBP5	1.681202	1.262735
LRG1	1.849892	1.226481
IGHA	2.543101	1.225014
LIMK2	1.711431	1.219404
PLIN4	1.328434	1.214794
GZMA	1.478285	1.205805
GK	1.752463	1.195020
CEACAM3	1.211504	1.189123
AIM2	0.966691	1.175317
IGHG	3.408794	1.164540
TRIM25	1.458591	1.158779
TBX21	-1.037250	1.132145
TXNIP	-1.161170	1.128549
DEFA4	1.358765	1.124833
IL1RAP	1.501989	1.109269
RBPJ	1.068847	1.069795

MEFV	1.508697	1.057649
SOD2	1.524129	1.055742
GUCY1B1	1.421701	1.038320
FPR2	1.542884	1.034898
TLR5	1.188285	1.022778
IRF1	1.124858	1.012595
BCL2	1.183276	1.005532
GBP4	0.756251	1.000475
TRAF3	1.461179	0.975868
TLR7	-0.561880	0.950557
CTLA4	0.923224	0.945350
PSTPIP1	0.866455	0.895451
DDX58	1.047182	0.882347
GNLY	-1.325010	0.877734
CD14	0.996623	0.851422
IL2	1.323086	0.839217
ADORA2A	1.012600	0.825309
TNFRSF10B	1.156804	0.819857
DDIT3	1.436722	0.813660
NAMPT	1.275634	0.813580
TNFRSF25	0.917765	0.812957
CCL11	1.030911	0.809576
IL6	1.341808	0.803138
LITAF	1.095755	0.792487
REL	0.985535	0.780621
GZMH	-1.020100	0.779251
CCL24	-0.967690	0.772388
CSF2RA	0.829453	0.772378
CXCL8	2.087914	0.764773
IFNA8	1.055495	0.763279
STAT1	1.069042	0.755243
IFI44	0.963688	0.751299
OAS1	0.714202	0.750276
PIK3R5	1.211209	0.747168
CD69	1.199391	0.744010
HSP90B1	0.843306	0.736947
IL6R	1.006933	0.735493
IL18R1	1.152490	0.733908
NFATC2	-0.742700	0.726665
CCL28	0.654777	0.723613
CD44	0.860292	0.718897
CCL14	-1.045050	0.711983
KLRC1	0.871362	0.705696
NCF1	1.032872	0.700318
CCL5	-0.816770	0.700290

CBLB	-1.596270	0.696001
HK3	0.745345	0.695936
IL2RG	0.821458	0.691834
BCL3	1.097945	0.670903
TAP1	0.727935	0.666131
FURIN	0.870091	0.662957
FGR	1.109656	0.661788
CCL4/L1/L2	1.215850	0.654698
PTGER4	0.734256	0.654039
IFI35	0.953576	0.640082
TRAF6	0.729996	0.638610
IFITM3	0.701602	0.636259
CSF2RB	0.851399	0.636041
OAS2	0.921733	0.635885
IL1B	1.921690	0.631355
TBK1	0.978156	0.627234
CCR1	1.121038	0.627160
PLAU	1.627335	0.619565
FOXP3	-0.695990	0.609162
CYSTM1	0.739931	0.602729
ATG10	0.771478	0.597560
SOCS3	1.101481	0.592761
CXCR4	0.941411	0.588420
ITGAX	1.052305	0.587407
TLR1	0.650994	0.579709
CARD17	0.857220	0.572685
CASP10	0.780602	0.564901
PTGS2	1.065016	0.563821
PAK1	0.912052	0.558678
IFITM1	0.675192	0.551409
HAVCR2	-0.690250	0.545073
TRIM33	0.435548	0.543606
DEFA1	1.454298	0.540325
RIPK2	0.663070	0.536979
PLEKHA1	-0.464870	0.530539
PRKCSH	-1.088050	0.528602
SELENOS	-0.599820	0.523230
CD84	-0.852820	0.522525
LTBR	1.176125	0.517378
BECN1	-0.473150	0.515987
TRIM56	0.538034	0.509145
CEBPB	0.504478	0.506625
KRAS	0.709053	0.499575
IL23R	0.716997	0.498322
CASP4	0.885992	0.497492

CD274	0.557088	0.494232
CXCL5	0.961918	0.489468
ACSL4	0.683526	0.488184
NAE1	-0.530390	0.487535
CPA3	0.782064	0.475204
NGLY1	0.574131	0.474010
XAF1	0.789433	0.473713
TRIM21	1.016499	0.473640
NLRP1	0.356578	0.467269
PFKFB3	0.833072	0.465944
SERPINA1	0.914164	0.465676
DIABLO	0.574005	0.465040
NFKB1	0.716693	0.463435
IL18	0.512430	0.459636
VRK3	-0.959110	0.456961
PLAUR	0.929611	0.455886
GBP2	0.523242	0.453422
HLA-DRB	0.548912	0.451978
GSK3B	0.593007	0.445989
NKG7	-0.392990	0.445756
FCGR2A	0.750151	0.443087
LYN	0.839872	0.441382
CUL1	0.522178	0.440726
GPX7	-0.623480	0.440618
ADAR	1.078556	0.439424
CXCL1	1.184665	0.434830
MYC	-0.671340	0.432477
PIK3C3	-0.309570	0.432421
PARP9	0.589450	0.432226
PRDM1	0.537679	0.429669
TPSAB1/B2	0.761233	0.425180
ARRB2	0.800488	0.423398
CD27	0.691878	0.420654
HMGB1	0.332970	0.418140
SELE	0.686421	0.416618
SLC11A1	0.852143	0.415560
AP1S2	1.141642	0.412696
HCST	-0.666240	0.411779
DTX3L	0.421053	0.409652
ATG3	0.712382	0.407453
TRAC	0.709131	0.406121
NCF2	0.800225	0.403175
ALOX5AP	0.815912	0.400588
NRAS	-0.962360	0.400262
CSF1	0.578418	0.398752

TGFBR2	-0.476910	0.398080
CD45RA	-0.585850	0.397779
TIMP2	-0.666490	0.395879
CRK	0.487534	0.394822
SIGIRR	-0.329280	0.394085
ACKR3	0.698830	0.392336
KDM6B	0.724099	0.390728
TAP2	0.316139	0.389131
GLB1	-0.451380	0.387716
TNFSF13B	0.675232	0.382358
MAP2K3	-0.595260	0.377455
KLRK1	-0.324890	0.376429
ATG12	0.424581	0.376280
CCL2	-0.697680	0.373936
TGFB1	-0.384650	0.372534
APOL6	0.685556	0.372219
LILRA5	0.666597	0.366602
PRKCA	-0.617450	0.366592
CD276	-0.694720	0.366486
PTK2B	0.475531	0.366453
ADGRE5	0.659497	0.364590
TCIRG1	-0.505320	0.364578
CD86	0.641903	0.364021
NFKB2	0.670156	0.361663
TGFB3	-0.343780	0.357046
CD3G	0.587812	0.354544
IFITM2	0.566974	0.351670
SCARB2	-0.437860	0.348893
XCR1	-0.506310	0.346733
TXN	0.462775	0.346570
BCL6	0.660400	0.346323
NEU1	0.426406	0.343095
CCR3	0.419193	0.343095
RAC2	0.663109	0.342815
NFAT5	-0.451380	0.339019
MIF	0.309417	0.338028
LGALS3	-0.502640	0.337884
ATG4A	-0.450500	0.337120
RAB7A	0.401846	0.335991
CD68	-0.507370	0.334353
LTF	0.978140	0.332453
NPC2	-0.407720	0.332303
NFATC1	-0.514570	0.331572
STAT3	0.566381	0.330590
VWF	-0.457770	0.329532

PTGER2	0.354326	0.325758
FCGR3A/B	0.505183	0.324102
PLCG1	-0.529220	0.322175
GUCY1A1	0.473201	0.321365
JAK3	0.600094	0.320915
UBE2L6	0.458021	0.318187
TANK	0.560772	0.317071
CXCL16	0.611151	0.316731
ALPL	-0.542550	0.316046
CSF3R	-1.173850	0.314938
TLR9	-0.335600	0.312358
DDX5	0.407990	0.310164
SMAD3	-0.513090	0.307407
PECAM1	-0.423850	0.304902
HSP90AA1	0.337727	0.303058
NOTCH1	0.498724	0.302974
MYD88	0.493354	0.302872
PIK3CB	0.507122	0.300958
IFI16	-0.929110	0.299370
C3AR1	0.607120	0.299370
SORT1	-0.547630	0.295642
ITGB2	-0.496910	0.291907
CTSZ	-0.534870	0.291593
TLN1	-0.387930	0.290736
CCNC	0.454106	0.290543
SMAD4	0.336596	0.290194
MME	-0.513930	0.289299
TCN2	-0.520330	0.289020
LTB	0.491782	0.288789
TYK2	0.266939	0.288612
LDHB	0.782755	0.288356
CX3CR1	0.382728	0.288241
PSEN1	-0.834000	0.287326
CXCR2	0.422595	0.287230
IRAK1	0.510673	0.286856
PRKCD	0.501108	0.285804
IKBKG	-0.356930	0.283676
TRIM22	0.463401	0.283580
CXCL3	0.548564	0.282548
NOX1	-0.503130	0.282318
TOLLIP	0.416521	0.280740
DNAJC10	0.276662	0.280247
FCGR1A/B	0.515094	0.277579
NFE2L2	0.467222	0.277045
PLEK	-0.987600	0.276301

VCAM1	-0.540520	0.275982
MAPK14	0.316126	0.274920
GSTM4	0.385043	0.272443
JAK1	-0.313740	0.272386
IL1RN	1.094236	0.263431
CCL18	1.191444	0.263103
IL6ST	-0.359180	0.262244
AKT1	-0.762740	0.261218
CD45R0	0.520577	0.259210
SIRPA	0.491508	0.258757
AKT3	-0.398480	0.255307
EIF2AK2	0.391579	0.253285
APOBEC3G	0.461003	0.252602
IFNGR2	0.439035	0.250636
UBA52	-0.343490	0.249639
LCN2	0.539835	0.246769
BCL2L1	-0.494890	0.243808
RASGRP4	0.374774	0.243004
HSP90AB1	-0.311100	0.242898
LILRB2	0.435950	0.241710
IL10RB	0.433338	0.241200
MLKL	0.327509	0.240967
NLRP3	0.471317	0.240164
AP1M1	-0.737670	0.238537
ATG7	0.504213	0.237026
RNF135	0.162927	0.236766
ICAM3	-0.296580	0.236662
TLR6	0.398072	0.236244
MAP3K3	-0.289800	0.235726
APP	-0.365030	0.234269
C1QBP	0.353216	0.233866
CCR2	0.391674	0.233689
MDFIC	-0.765000	0.232342
ATG13	-0.178800	0.231462
ATF6	-0.738350	0.230109
HPGD	-0.424380	0.229459
JUN	-0.377940	0.229006
C3	0.768170	0.226909
IL17RA	-0.288660	0.225911
LAT	-0.358420	0.225739
FCGRT	-0.343770	0.222603
ANPEP	-0.383260	0.221493
ATP6V1B2	0.412416	0.219112
LAMP1	-0.234650	0.217799
CD4	-0.386730	0.217549

PIK3CG	0.370146	0.216914
STAT2	0.357169	0.215553
CTSA	-0.380020	0.215327
MAVS	-0.534390	0.213519
IL10RA	0.298415	0.212658
MAP2K4	0.533401	0.211477
PRCP	-0.360650	0.211051
IL4R	0.322311	0.210939
STAT5A	-0.696380	0.210750
CREBBP	-0.306390	0.204477
AIF1	0.307867	0.204450
ATM	-0.722510	0.204239
VSIR	-0.399210	0.200318
RHOG	0.302392	0.196979
CR1	0.434567	0.196157
FYN	-0.640810	0.195523
CFLAR	0.308570	0.195159
RAB31	-0.709040	0.194214
MAP2K7	-0.580210	0.193301
HCK	0.299902	0.190653
IL27RA	-0.232510	0.189698
CDK4	-0.346230	0.185714
ALOX5	0.334695	0.183058
CXCL12	0.411314	0.180679
PELI1	-0.254220	0.180237
RAF1	0.352200	0.180023
CBL	0.569080	0.179183
MAPKAPK2	0.280918	0.179078
TRAF2	-0.280980	0.178525
PDHB	-0.538040	0.178197
MAPK1	-0.235750	0.176267
ITGAM	0.348517	0.176054
MS4A7	0.383820	0.175457
NCF4	0.354468	0.173242
IGFBP7	-0.353430	0.172360
HLA-DRA	0.278493	0.171300
MARCO	0.378862	0.167929
CBFB	-0.535950	0.167462
NEO1	-0.217870	0.167083
HMOX1	-0.675380	0.165003
TPP1	-0.236120	0.164376
TAB1	-0.232980	0.163061
SOD1	-0.260970	0.162947
RNASEL	-0.251540	0.162066
GNS	-0.299140	0.161737

CTSW	-0.166760	0.161609
F5	-0.274620	0.159591
SYK	0.243478	0.159519
MGAM	-0.308520	0.159219
TNFRSF1A	0.555848	0.158769
ACVR1	-0.291920	0.158069
TLR2	0.305748	0.155915
TNFSF10	0.221558	0.155283
CD81	-0.273030	0.154723
IRAK3	0.308549	0.152615
UBE2N	-0.227200	0.150527
NFATC3	0.455581	0.148595
CD22	0.285871	0.147773
RPS6KA1	0.287325	0.146999
EVL	-0.289600	0.146384
VAMP3	-0.253310	0.145674
CD3E	0.248388	0.144583
MAPK13	0.211613	0.143348
FOXO1	-0.234610	0.139580
IFNA6	0.275080	0.138206
CSF1R	-0.267990	0.138113
PSMB9	0.208688	0.137601
CALM1	-0.177370	0.137528
ALPK1	0.248388	0.137159
LILRA3	-0.190960	0.137105
NDUFS8	-0.450860	0.136548
SAMHD1	0.224188	0.136381
TLR8	-0.103480	0.136252
HLA-DPA1	0.227714	0.135994
MARCKS	0.270853	0.134718
MVP	-0.395300	0.134025
THOP1	0.250407	0.134011
MAFB	-0.245720	0.133000
STING1	-0.210940	0.132678
RB1CC1	0.208118	0.131966
IL5	0.240889	0.129618
ENTPD1	-0.238400	0.129087
MT2A	-0.222530	0.128184
STAT5B	0.161945	0.126392
ATF2	-0.211460	0.125814
LTA4H	0.202959	0.125697
IL33	0.275542	0.124617
IL7R	0.189891	0.123161
OSM	0.480511	0.122402
RNF114	0.208037	0.120706

EIF3F	-0.158560	0.119726
IL16	-0.180370	0.119424
ZAP70	-0.176920	0.118960
SELL	0.251238	0.118472
EIF2AK3	0.164609	0.116349
CTSS	-0.218740	0.114667
ETS1	-0.223670	0.114218
SSR1	-0.347830	0.112100
BST2	0.206490	0.110283
STT3B	0.227629	0.110245
PIK3CA	0.167016	0.108944
PSMB8	0.090091	0.107957
CGAS	0.108479	0.107505
RPS6KA3	-0.199450	0.106164
ACKR4	0.190508	0.105632
MAP3K8	-0.200820	0.102278
MS4A1	-0.205760	0.101628
MAP3K7	-0.102040	0.100925
GADD45B	0.218468	0.100161
FAS	0.286817	0.099753
HLA-DMB	-0.207820	0.099479
ATP6V0D1	0.186297	0.096044
FOS	-0.217150	0.095939
CXCL2	0.236711	0.095097
SPI1	0.204107	0.094871
PTPRC	0.190898	0.094830
STRAP	0.321124	0.094047
JUNB	0.175752	0.091735
CD163	-0.184740	0.091292
IL13RA1	0.128630	0.091065
PIK3CD	0.169751	0.089588
KPNB1	-0.321230	0.089110
APEX1	0.133856	0.087560
SP100	0.310004	0.086892
HLA-DQA	0.171033	0.085594
AP1G1	0.101671	0.084818
IFNA4/7/10/17/21	0.161696	0.084431
PARP1	0.255340	0.084115
NLRC4	0.142512	0.082901
ATP6AP2	-0.266470	0.082206
FCAR	0.193103	0.082126
PANX1	-0.173330	0.081969
STAT6	0.112683	0.081742
ITPR3	-0.086870	0.081445
ACSL1	0.208397	0.079265

CD28	0.156504	0.078589
ACOX1	0.215147	0.078412
TLR4	-0.134420	0.076898
IL2RB	-0.146100	0.076145
ERN1	0.163499	0.075331
PSMB10	0.124905	0.075124
IFNAR1	0.116980	0.075105
PLCG2	0.144590	0.075068
BNIP3	0.137130	0.072992
JAML	-0.162230	0.072530
APBB1IP	0.258573	0.072437
MAP2K2	-0.108650	0.072257
LRRK2	-0.141140	0.071925
ITGAL	-0.147120	0.071112
TAB2	0.152971	0.070744
GCA	0.155004	0.070505
MS4A4A	0.184094	0.069832
CD3D	0.136236	0.069672
RIPK3	-0.084270	0.069013
ATF4	0.091583	0.068488
CTSL	-0.173640	0.067243
PXN	0.104569	0.065720
HLA-DPB1	0.148120	0.065516
RBCK1	-0.222650	0.065436
IRAK4	0.067457	0.064707
SIGLEC5	0.145894	0.062940
SMAD5	0.094842	0.062111
IKBKB	-0.121290	0.059424
PPIA	0.065923	0.058401
CASP8	-0.111410	0.057608
KLRB1	0.131484	0.057132
SOCS1	0.114752	0.055285
PTPN6	0.116459	0.054590
TYROBP	-0.122760	0.053020
CXCL14	-0.208040	0.051915
MTOR	-0.086940	0.050886
CXCR1	-0.110250	0.050860
HLA-A	0.087223	0.050033
C5AR1	0.115271	0.047575
FPR1	0.119611	0.046689
CD59	-0.082700	0.046606
MAPK9	0.125457	0.045444
LAMP2	0.094090	0.045038
IRF4	0.075844	0.044342
RELA	0.068192	0.043694

CCR5	0.085890	0.043295
EGLN1	0.065423	0.041897
OS9	0.060831	0.041882
RNF31	0.076236	0.041239
CCL3/L1/L3	0.185336	0.040965
LEF1	-0.097940	0.040738
MAP1LC3A	-0.080430	0.037922
GBA	-0.080170	0.037217
HLA-DMA	0.087133	0.037195
MAP3K1	0.072912	0.035973
DNAJA2	-0.068470	0.035899
MAPK8	0.055421	0.035376
PRKCQ	-0.044040	0.035065
AHR	0.064729	0.034444
MSRA	0.101947	0.033946
DDOST	0.055385	0.032798
JAK2	-0.067560	0.031957
IFNAR2	-0.110680	0.030719
HLX	0.051165	0.030711
BCR	0.028157	0.030632
KIR2DL1	-0.067510	0.030117
MRC1	-0.059670	0.029829
SEM1	-0.068090	0.029079
PSAP	-0.055760	0.028854
DYSF	0.061158	0.028659
DDAH2	-0.048140	0.028445
CASP1	0.056806	0.027206
CCL21	-0.113220	0.025768
NT5E	0.028065	0.025402
THBS1	0.045796	0.023933
WAS	0.056106	0.023604
HLA-B	0.038197	0.022636
CXCL9	-0.048590	0.022491
IGHM	0.080618	0.022214
SUGT1	0.068998	0.021987
PTPN4	0.032790	0.021254
HLA-C	-0.033810	0.021036
LANCL1	-0.040640	0.019163
S100A12	0.048230	0.018868
RACK1	-0.025520	0.018609
TRAM1	0.025522	0.017040
SP1	0.026387	0.015974
LAT2	-0.034520	0.014827
IL1R2	-0.036830	0.014412
GLA	-0.017620	0.014252

LILRA6	0.024200	0.014228
ULK1	-0.014050	0.014222
RIPK1	-0.018380	0.014030
PIK3R4	0.024453	0.011926
AKT2	-0.042080	0.011847
MAF	-0.022480	0.011568
YWHAQ	0.017940	0.011241
CAP1	0.019507	0.010285
MCL1	-0.018860	0.008526
DERL1	-0.029760	0.007473
RAB5C	0.026337	0.007066
IL1R1	-0.010780	0.005892
TBXAS1	0.013653	0.005435
HLA-E	0.009422	0.004790
IL32	0.006886	0.002835
LCP1	-0.005380	0.002032
ACE	0	0
ACKR2	0	0
ACSL3	0	0
ADGRG3	0	0
ADRA	0	0
AGT	0	0
AICDA	0	0
ALOX12	0	0
ALOX15	0	0
BATF	0	0
BDKRB1	0	0
BDKRB2	0	0
BLK	0	0
BPI	0	0
C2	0	0
C5	0	0
CARD11	0	0
CARD16	0	0
CASP5	0	0
CCL1	0	0
CCL13	0	0
CCL15	0	0
CCL16	0	0
CCL17	0	0
CCL19	0	0
CCL20	0	0
CCL22	0	0
CCL23	0	0
CCL25	0	0

CCL26	0	0
CCL27	0	0
CCL7	0	0
CCL8	0	0
CCR10	0	0
CCR4	0	0
CCR6	0	0
CCR7	0	0
CCR8	0	0
CCR9	0	0
CCRL2	0	0
CD19	0	0
CD1E	0	0
CD2	0	0
CD209	0	0
CD244	0	0
CD247	0	0
CD38	0	0
CD40	0	0
CD40LG	0	0
CD6	0	0
CD70	0	0
CD79A	0	0
CD79B	0	0
CD80	0	0
CD8A	0	0
CD8B	0	0
CHUK	0	0
CLFA	0	0
CLFB	0	0
CRP	0	0
CSF2	0	0
CSF3	0	0
CSGB	0	0
CTSG	0	0
CUPA1	0	0
CUPA4	0	0
CX3CL1	0	0
CXCL10	0	0
CXCL11	0	0
CXCL13	0	0
CXCL17	0	0
CXCL6	0	0
CXCR3	0	0
CXCR5	0	0

CXCR6	0	0
CYP2E1	0	0
DEFB103A/B	0	0
DHX58	0	0
EBI3	0	0
ELANE	0	0
EOMES	0	0
EPHX2	0	0
FAM30A	0	0
FASLG	0	0
FBXO6	0	0
FCRL2	0	0
FCRL4	0	0
FLGM	0	0
FLIA	0	0
FNBA	0	0
FNBB	0	0
GAB2	0	0
GATA3	0	0
GBP1	0	0
GZMB	0	0
HAMP	0	0
HDC	0	0
HLA-DOB	0	0
HLA-DQB1	0	0
HSD11B1	0	0
ICAA	0	0
ICAB	0	0
ICAC	0	0
ICOS	0	0
ICOSLG	0	0
IDO1	0	0
IFNA1/13	0	0
IFNA14/16	0	0
IFNA2	0	0
IFNA5	0	0
IFNB1	0	0
IFNG	0	0
IFNK	0	0
IFNL1	0	0
IFNL2/3	0	0
IFNL4	0	0
IFNLR1	0	0
IFNW1	0	0
IGHD	0	0

IGHE	0	0
IKBKE	0	0
IL10	0	0
IL11	0	0
IL11RA	0	0
IL12A	0	0
IL12B	0	0
IL12RB1	0	0
IL12RB2	0	0
IL13	0	0
IL13RA2	0	0
IL15	0	0
IL15RA	0	0
IL17A	0	0
IL17B	0	0
IL17C	0	0
IL17D	0	0
IL17F	0	0
IL17RB	0	0
IL17RC	0	0
IL17RD	0	0
IL17RE	0	0
IL18BP	0	0
IL18RAP	0	0
IL19	0	0
IL1A	0	0
IL1F10	0	0
IL1RAPL1	0	0
IL1RAPL2	0	0
IL1RL1	0	0
IL1RL2	0	0
IL20	0	0
IL20RA	0	0
IL20RB	0	0
IL21	0	0
IL21R	0	0
IL22	0	0
IL22RA1	0	0
IL22RA2	0	0
IL23A	0	0
IL24	0	0
IL25	0	0
IL26	0	0
IL27	0	0
IL2RA	0	0

IL3	0	0
IL31	0	0
IL31RA	0	0
IL34	0	0
IL36A	0	0
IL36B	0	0
IL36G	0	0
IL36RN	0	0
IL37	0	0
IL3RA	0	0
IL4	0	0
IL5RA	0	0
IL7	0	0
IL9	0	0
IL9R	0	0
IRF3	0	0
ITGAE	0	0
ITGB7	0	0
ITK	0	0
ITLN1	0	0
KIR2DL3	0	0
KIR3DL1/2	0	0
KLRD1	0	0
LAG3	0	0
LAMP3	0	0
LCK	0	0
LECA	0	0
LECB	0	0
LIF	0	0
LTC4S	0	0
MAP3K5	0	0
MKNK1	0	0
MS4A2	0	0
MX1	0	0
NCR1	0	0
NCR3	0	0
NFATC4	0	0
NLRC5	0	0
NOS2	0	0
NTNG2	0	0
OAS3	0	0
OASL	0	0
P2RX7	0	0
PDCD1	0	0
PDCD1LG2	0	0

PELI2	0	0
PIK3R3	0	0
PIK3R6	0	0
PLG	0	0
PNOC	0	0
PRF1	0	0
RASGRP1	0	0
RELB	0	0
RGMA	0	0
RPS6KB1	0	0
RUNX3	0	0
SH2D1A	0	0
SPIB	0	0
STAT4	0	0
TCF7	0	0
TCL1A	0	0
TGFB2	0	0
TIFA	0	0
TIGIT	0	0
TLR3	0	0
TMEM140	0	0
TMPRSS2	0	0
TNF	0	0
TNFRSF17	0	0
TNFRSF18	0	0
TNFRSF4	0	0
TNFRSF9	0	0
TNFSF18	0	0
TNFSF4	0	0
TNFSF9	0	0
TRAT1	0	0
TRIM5	0	0
TRIM6	0	0
TXK	0	0
ULK2	0	0
XCL1/2	0	0

Table S7: Comparison of infected bone samples (n=4) versus a non-infected control group (n=16). P-values were adjusted using the Benjamini–Hochberg false discovery rate (FDR). Significant genes are indicated in bold. (-log (p-adjusted > 1.3)).

Symbol	log2FoldChange	-log(p-adjusted)
GK	5.554434	2.497942
PFKFB3	4.702782	2.497942
PLAU	6.167316	2.497942
CXCL1	5.774557	2.209719
PLEK	5.922365	2.209719
PTGS2	5.310657	2.209719
SOD2	4.847201	2.209719
CXCL2	4.608546	2.166400
CYSTM1	3.094846	2.163324
ACSL1	4.927587	2.002610
ADORA2A	3.219856	2.002610
CCL4/L1/L2	4.733473	2.002610
PTK2B	3.005220	2.002610
SIGLEC5	4.261732	1.922264
NLRP3	3.772884	1.791400
GADD45B	3.638976	1.716439
LCP2	4.372443	1.571106
SLC2A3	4.264510	1.545330
ATG7	3.909483	1.527987
PLAUR	4.239118	1.527987
IRAK3	3.441078	1.498854
MEFV	3.771376	1.498854
NAMPT	3.822247	1.498854
CD44	2.656683	1.339452
BCL3	3.490210	1.339325
CEACAM3	2.594361	1.339325
KDM6B	3.470361	1.339325
PRKCD	3.223380	1.339325
C3AR1	3.583541	1.303480
CXCL3	3.299704	1.303480
FPR2	3.661225	1.303480
ITGAX	3.636256	1.303480
MAPK13	2.271857	1.279957
PIK3R5	3.475551	1.200064
NFKB1	2.857529	1.196557
NOD2	3.146382	1.196557

IL18R1	3.278859	1.166647
IL4R	2.355504	1.157644
LIMK2	3.428302	1.157644
NCF2	3.526110	1.157644
FCAR	3.289989	1.114553
NFKB2	3.055751	1.074584
IFNGR2	2.730231	1.068725
LCP1	3.293739	1.041204
TRAF3	3.155956	1.039198
VEGFA	3.685924	1.026707
CR1	3.296033	1.005750
ERN1	2.893378	1.005750
LYN	3.226617	1.005750
PAK1	2.926937	1.005750
SLC11A1	3.390420	1.005750
IFITM2	2.599003	1.001883
SERPINA1	3.383764	1.001883
CD22	2.525906	0.979076
BCL6	2.991558	0.966093
CSF2RB	2.421271	0.966093
IL2RG	2.196008	0.966093
IFITM1	2.122026	0.965675
HCK	2.208952	0.959437
DDIT3	3.257798	0.939403
CEBPB	1.701009	0.900304
ALOX5AP	3.198827	0.891638
GBP2	1.837021	0.891638
RAC2	2.861576	0.891638
FGR	2.852216	0.874692
LILRA5	2.704544	0.874692
LRG1	3.061726	0.838188
MARCKS	2.442407	0.838188
ATP6V1B2	2.466292	0.812172
TLR2	2.516958	0.800407
VWF	-2.101895	0.800407
CASP4	2.686990	0.776873
ALOX5	2.366407	0.764178
CXCL8	4.452289	0.764178
DYSF	2.291183	0.764178
IL6R	2.289507	0.764178
RAB7A	1.616493	0.764178
S100A12	2.856568	0.764178
REL	2.078687	0.749213
FPR1	2.870523	0.748110
CSF2RA	1.748938	0.729066

C5AR1	2.595557	0.716366
CD274	1.604071	0.707948
NLRC4	1.822002	0.707948
RHOG	1.813222	0.707948
ZBP1	2.404984	0.707948
TANK	2.288770	0.707418
SOCS3	2.710970	0.690542
LITAF	2.194195	0.671459
STAT3	2.183332	0.671459
FCGR2A	2.308314	0.664624
IGHA	3.773477	0.653509
PECAM1	-1.760520	0.653509
RASGRP4	1.862496	0.653509
TLR6	2.021811	0.650783
OSM	4.186150	0.642250
ARRB2	2.439668	0.627485
CSF3R	2.825360	0.627485
NCF4	2.299428	0.627485
CXCL5	2.593259	0.623618
IGFBP7	-2.279460	0.623618
TBK1	2.156039	0.603040
IFITM3	1.536051	0.593467
XBP1	1.856706	0.593467
IL1B	4.057309	0.567706
TRIM25	2.127996	0.567706
CD45RB	1.352660	0.558396
NCF1	2.065481	0.547484
DIABLO	1.487446	0.540168
NFE2L2	1.867331	0.534373
IL10RB	1.905720	0.526331
MAP2K3	1.854066	0.526331
GLA	1.077286	0.511473
IL1R2	2.237397	0.511473
TYK2	1.017209	0.505505
DEFA1	3.307754	0.496477
CCR1	2.241586	0.494129
PLAT	-1.870958	0.478343
PTGER4	1.411384	0.478343
PSMB8	-0.756144	0.472688
CD45R0	2.069356	0.464193
IL1RAP	2.011973	0.464193
KRAS	1.627376	0.454834
MAP1LC3A	1.786338	0.454834
NGLY1	1.393621	0.454834
CCL14	-1.922167	0.449064

IL6	2.180748	0.449064
C3	2.370427	0.439473
CD69	2.045515	0.439473
CD86	1.824811	0.431471
F5	1.570316	0.431471
MCL1	1.775543	0.423234
GCA	1.895328	0.422753
CARD17	1.723461	0.415691
STAT5B	1.127516	0.413310
RPS6KA1	1.701890	0.411678
BCR	-0.763486	0.406882
CFLAR	1.454106	0.406882
CASP1	1.624691	0.399489
JUNB	1.607113	0.399489
LAT	-1.454654	0.392034
NT5E	-0.847098	0.392034
TYROBP	1.795840	0.392034
ATP6V0D1	1.547126	0.385833
IFI27	-1.615932	0.385833
LTA4H	1.310298	0.385833
TNFSF13B	1.740945	0.385833
JAK3	1.808127	0.384815
JAK2	1.643394	0.382227
TRAF6	1.279805	0.382227
ALPL	1.622469	0.374748
IL10RA	1.251088	0.374748
CCL3/L1/L3	3.234992	0.360633
GUCY1A1	1.382264	0.360633
IL33	-1.795779	0.360633
IRF7	1.478984	0.360633
SYK	1.249866	0.360633
TOLLIP	1.345775	0.360633
ATG3	1.665937	0.349323
MAP3K8	1.492043	0.348907
ISG15	-1.655352	0.346783
XAF1	-1.666904	0.342055
IL1RN	3.319271	0.335703
GBA	1.517058	0.334427
HMOX1	2.011568	0.334427
SIGIRR	-0.773035	0.334427
FAS	1.278926	0.328485
ITGAM	1.531944	0.328485
PRKCQ	0.904216	0.324756
CD276	-1.684684	0.311048
IGHG	3.679918	0.302026

SIRPA	1.546357	0.298059
PRCP	-1.384015	0.297049
HSP90B1	1.153956	0.284893
RIPK2	1.157156	0.284893
MGAM	1.476638	0.278343
PTPRC	1.413975	0.278343
SPI1	1.490719	0.277308
AIF1	1.119709	0.275246
CXCL16	1.534129	0.275246
TCIRG1	1.153937	0.274828
APBB1IP	1.400679	0.273948
PLEKHA1	-0.799114	0.269685
CCL18	3.267674	0.263283
SELENOS	0.995485	0.263283
ETS1	-1.345468	0.260419
HK3	0.974536	0.260419
STAT5A	2.372551	0.260419
TPP1	0.995703	0.260419
CD14	1.147442	0.259809
MAPK14	0.849232	0.223164
PIK3CB	1.244581	0.223164
LEF1	-1.459432	0.216539
PLCG2	1.190540	0.214208
TGFBR2	-0.937431	0.214208
CCL24	-1.153820	0.210901
MS4A4A	1.508147	0.210901
CCL5	-1.032421	0.206568
GBP4	-0.750651	0.206568
TGFB3	-0.724040	0.206568
APP	-1.086657	0.204148
CD27	1.257189	0.204148
LCN2	1.483093	0.199048
CD59	-1.016954	0.194241
IL18	0.843819	0.194241
RIPK3	0.696132	0.193400
TCN2	-1.239961	0.193400
LANCL1	-1.187307	0.188778
SP100	1.283860	0.186935
STAT6	0.811956	0.186935
STING1	-0.963201	0.186935
PIK3CG	1.096101	0.186687
GPX7	-1.035852	0.186615
CPA3	1.223032	0.176671
IRAK1	1.155489	0.174881
CD45RA	-1.021695	0.166010

HPGD	1.142958	0.164269
TRIM22	1.027421	0.163730
CALM1	-0.728042	0.162905
LILRB2	1.113935	0.162905
LTF	1.934252	0.162905
MTOR	-0.877872	0.162905
PIK3R4	1.022568	0.162905
TAB2	1.191921	0.162905
ADAR	1.624302	0.152527
DEFA4	1.074184	0.152527
FCGR1A/B	1.135429	0.152527
LAT2	1.154206	0.152527
LILRA6	0.830075	0.152527
MYD88	0.989405	0.152527
PLIN4	1.029747	0.152527
RSAD2	0.780569	0.152527
IL1R1	0.894960	0.151737
VRK3	0.816052	0.151737
RNF135	0.414021	0.151206
CD68	0.889560	0.146593
DNAJA2	0.923971	0.145765
IFIH1	0.809251	0.145765
LTBR	0.898185	0.145765
PIK3CD	0.972846	0.145765
CRK	0.771040	0.143119
TLR1	0.747044	0.141101
AIM2	0.726511	0.137501
CXCR1	1.067114	0.137501
CAP1	0.861054	0.136067
TNFRSF25	-0.858885	0.136067
IKBKG	0.722923	0.131811
IFI44	-0.900403	0.130952
CD81	-0.898750	0.129854
CXCR2	0.815230	0.129854
CXCR4	1.059841	0.129854
EVL	-0.991981	0.129854
RNF114	0.836994	0.129854
ALPK1	0.895378	0.129778
SELL	1.062943	0.129778
EIF2AK3	0.672835	0.122586
IFI6	-0.976387	0.122586
IGHM	1.589951	0.122586
SMAD5	0.696305	0.122586
TAP2	0.458214	0.122586
RNASEL	0.768859	0.122243

ACSL4	0.829904	0.121927
FOS	1.066858	0.121927
LTB	0.910573	0.121927
PTPN6	0.925095	0.121927
UBA52	0.730542	0.121927
APEX1	-0.665782	0.113766
BCL2L1	1.046941	0.113766
CD3D	-0.864714	0.113766
IL17RA	0.658151	0.113766
MME	0.926184	0.113766
TLN1	0.699358	0.113766
TRIM33	0.481697	0.113384
WAS	0.988753	0.113384
CBL	0.912052	0.110310
IRF1	0.753596	0.110310
MDFIC	-0.984111	0.110310
TLR4	0.753038	0.110310
TLR5	0.821171	0.110310
TNFRSF10B	0.919952	0.110310
TBXAS1	0.989333	0.109045
CD3E	0.789580	0.107010
MAP2K7	0.841864	0.107010
MLKL	0.646028	0.107010
ATG10	0.743225	0.106996
CD163	0.831011	0.106996
IL2	1.016822	0.106996
IL32	-0.922544	0.106996
KIR2DL1	0.864934	0.106996
STRAP	1.397649	0.106996
ACOX1	0.660344	0.106188
PRDM1	0.641350	0.106188
ULK1	-0.372774	0.103434
EIF2AK2	-0.701333	0.103172
IFNA4/7/10/17/21	0.768742	0.103172
PXN	0.623508	0.103172
SEM1	-0.895233	0.103172
IFNAR2	0.798871	0.102077
TIMP2	-0.830621	0.102077
GSTM4	0.667425	0.102049
BECN1	-0.455758	0.098818
CBLB	1.304695	0.098818
PRKCA	-0.812592	0.098818
SCARB2	-0.582834	0.098818
NEU1	0.560256	0.098238
IL23R	0.741495	0.098032

JAK1	0.518368	0.098032
NRAS	-1.155629	0.098032
VAMP3	0.700677	0.098032
FURIN	0.700332	0.095313
HERC5	0.760812	0.095313
FCGR3A/B	0.681824	0.094204
PELI1	0.571333	0.094204
ACKR4	0.623337	0.091552
ACVR1	-0.690555	0.091552
AKT1	1.185367	0.091552
ANPEP	0.703862	0.091552
ATF4	0.450032	0.091552
ATG12	0.487017	0.091552
BST2	-0.685747	0.091552
CXCL12	0.916868	0.091552
ENTPD1	-0.703357	0.091552
FOXP3	-0.567236	0.091552
GUCY1B1	0.806082	0.091552
HLA-A	0.589083	0.091552
HLA-B	0.543451	0.091552
IFIT3	-0.628031	0.091552
IFNAR1	0.536806	0.091552
KLRC1	0.610196	0.091552
MIF	0.385709	0.091552
MRC1	0.656878	0.091552
MS4A7	0.832034	0.091552
MT2A	0.625057	0.091552
NAE1	-0.875729	0.091552
NDUFS8	1.179905	0.091552
NFATC2	-0.514409	0.091552
RBPJ	0.589326	0.091552
SOD1	-0.598560	0.091552
YWHAQ	-0.502215	0.091552
CXCL14	1.318040	0.090401
FYN	-1.192956	0.090049
RELA	0.492168	0.086962
CBFB	1.089284	0.083769
CCL11	0.627675	0.083769
CD84	-0.670601	0.083769
CDK4	-0.655479	0.083769
CTLA4	0.492222	0.083769
GBP5	0.712198	0.083769
GNS	0.608377	0.083769
HLA-C	0.474793	0.083769
PSTPIP1	-0.467923	0.083769

RACK1	-0.403448	0.083769
RAF1	0.685662	0.083769
RPS6KA3	-0.596351	0.083769
TRAC	-0.710493	0.083769
VSIR	0.717415	0.083769
LAMP2	1.105815	0.081542
DNAJC10	-0.350661	0.076987
TRIM21	0.493162	0.076564
AP1G1	0.355790	0.076550
CSF1	0.539602	0.076550
IKBKB	0.596043	0.076550
MSRA	0.507687	0.076550
PLCG1	-0.596278	0.076550
SMAD4	0.398402	0.076550
SP1	0.457234	0.076550
ZAP70	-0.458239	0.076550
ATG13	-0.228669	0.076536
CCR3	0.431121	0.076536
CTSS	0.539025	0.076536
HCST	0.557440	0.076536
HSP90AA1	0.369837	0.076536
JAML	0.639355	0.076536
MAPKAPK2	0.483022	0.076536
AKT3	0.501573	0.072605
PYCARD	0.468477	0.071475
IFNA8	0.571622	0.069355
DTX3L	-0.339765	0.065896
LAMP1	0.312776	0.065896
PIK3CA	0.424858	0.065896
HLA-DPB1	-0.579249	0.061804
LGALS3	0.458477	0.059351
STAT2	-0.467222	0.059351
CTSL	-0.619728	0.058402
LRRK2	0.510533	0.057698
CCR2	0.486399	0.054484
IRAK4	0.260360	0.054484
NFATC1	-0.468292	0.054484
SORT1	-0.523562	0.054461
BNIP3	0.445264	0.054318
CTSZ	-0.497411	0.054318
EGLN1	-0.353483	0.054318
GNLY	-0.581077	0.054318
ITPR3	-0.251200	0.054318
MAPK1	0.355923	0.054318
OAS2	0.487446	0.054318

VCAM1	0.537246	0.053648
IL6ST	-0.368958	0.053212
KPNB1	0.518325	0.053212
CASP10	0.429684	0.049530
CX3CR1	-0.352302	0.049530
PSAP	0.396841	0.049530
NOTCH1	0.438029	0.048889
RBCK1	0.454566	0.046785
GLB1	-0.307471	0.044552
CCL2	-0.503238	0.044225
CREBBP	0.361624	0.043660
LDHB	-0.388354	0.043660
NLRP1	0.213956	0.043660
PTPN4	0.310849	0.043660
SOCS1	-0.428517	0.043660
ATM	0.466534	0.043516
ATP6AP2	0.380588	0.043516
DDX58	0.372923	0.043516
TNFRSF1A	0.760387	0.043516
CTSW	0.218071	0.038683
DDAH2	0.307263	0.038683
IL5	0.373776	0.038683
MAPK9	0.519512	0.038683
NKG7	-0.226169	0.038683
PPIA	-0.216892	0.038683
TXN	0.308956	0.038683
TXNIP	-0.339872	0.038683
AHR	-0.322021	0.036679
CCNC	-0.335236	0.036679
IFI35	0.376218	0.036679
NFAT5	0.289507	0.036679
PSMB10	-0.301170	0.036679
RAB31	0.727786	0.036679
TGFB1	-0.234914	0.036679
TLR9	-0.227069	0.036679
C1QBP	-0.299614	0.034547
HSP90AB1	-0.250698	0.031538
NEO1	0.237324	0.031538
MAP2K2	0.252686	0.031364
CCR5	0.322827	0.029908
BCL2	0.302682	0.027322
TPSAB1/B2	0.370439	0.026118
MAP2K4	0.263972	0.025176
CCL21	0.634613	0.024841
HLA-DQA	-0.304250	0.024841

IFI16	0.572386	0.024841
PRKCSH	0.436653	0.024841
STT3B	-0.325576	0.024841
ATG4A	-0.244045	0.023943
THOP1	-0.292041	0.023943
PIK3C3	-0.136240	0.023272
ATF2	0.243992	0.020147
CD3G	-0.283072	0.020147
GSK3B	0.234842	0.020147
KLRK1	0.143200	0.020147
MAF	-0.236508	0.020147
MAFB	0.258932	0.020147
PDHB	-0.450836	0.020147
SMAD3	-0.268753	0.020147
SUGT1	-0.229868	0.018589
AKT2	0.186413	0.017984
AP1M1	-0.367613	0.017984
AP1S2	-0.391652	0.017984
APOL6	0.191326	0.017984
CDH1	0.183237	0.017984
CTSA	-0.194935	0.017984
CXCL9	-0.260016	0.017984
DDOST	-0.167797	0.017984
FCGRT	0.152126	0.017984
GZMH	-0.170855	0.017984
HLA-DPA1	-0.185375	0.017984
HLA-DRA	-0.177625	0.017984
HLX	0.146015	0.017984
HMGB1	0.109831	0.017984
ICAM3	0.169276	0.017984
IFIT2	0.221335	0.017984
IL13RA1	-0.172172	0.017984
IL2RB	-0.228342	0.017984
IRF4	-0.189162	0.017984
IRF9	0.248284	0.017984
ITGB2	0.161350	0.017984
MAP3K1	-0.163666	0.017984
MAP3K7	-0.096772	0.017984
MAPK8	-0.167966	0.017984
MARCO	-0.232446	0.017984
MAVS	0.153852	0.017984
MS4A1	-0.213758	0.017984
MYC	-0.216000	0.017984
NOX1	-0.257204	0.017984
OAS1	-0.129063	0.017984

OS9	0.164752	0.017984
PARP9	-0.178642	0.017984
PSEN1	0.340552	0.017984
RB1CC1	0.161892	0.017984
TBX21	0.146549	0.017984
THBS1	0.221720	0.017984
TLR7	-0.079275	0.017984
TLR8	-0.079727	0.017984
TRAF2	-0.202607	0.017984
TRAM1	-0.128777	0.017984
TRIM56	-0.120839	0.017984
CD28	0.153992	0.016420
LILRA3	0.117171	0.016420
MVP	0.235907	0.016420
NFATC3	-0.251575	0.016420
HAVCR2	-0.122156	0.015271
HLA-DMA	0.158803	0.015271
HLA-DMB	-0.148073	0.015271
IL7R	-0.113431	0.015271
PARP1	-0.125531	0.015271
UBE2N	0.111839	0.014751
SAMHD1	-0.108294	0.014493
TNFSF10	-0.173536	0.013497
IFIT1	-0.113849	0.012554
JUN	0.113143	0.012554
TAP1	-0.092056	0.012554
XCR1	-0.100343	0.010536
IFNA6	0.112470	0.009539
IL27RA	0.070735	0.009539
ADGRE5	0.099404	0.009260
CD36	-0.099220	0.009260
CSF1R	0.101703	0.009260
FOXO1	0.078169	0.009260
GZMA	0.089320	0.009260
HLA-DRB	-0.062553	0.009260
ITGAL	0.086223	0.009260
KLRB1	-0.093028	0.009260
PSMB9	0.065713	0.009260
RAB5C	-0.142197	0.009260
RNF31	-0.089005	0.009260
UBE2L6	0.067114	0.009010
ATF6	0.113297	0.007843
CASP3	0.090787	0.007843
CASP8	0.065611	0.007843
CGAS	-0.030606	0.007843

PANX1	0.064130	0.007843
SSR1	-0.051730	0.007843
STAT1	0.058299	0.007843
TAB1	-0.049804	0.007843
SELE	0.057915	0.007518
IL16	0.040335	0.006929
MAP3K3	-0.032814	0.006208
DERL1	-0.040139	0.003775
PTGER2	-0.022771	0.003775
ACKR3	-0.032421	0.003283
RIPK1	0.018944	0.003283
APOBEC3G	0.012666	0.002106
CCL28	-0.014214	0.002106
CD4	0.005885	0.002106
CUL1	0.006008	0.002106
DDX5	0.007471	0.002106
EIF3F	0.009781	0.002106
HLA-E	-0.007998	0.002106
NPC2	0.014053	0.002106
WIPI1	-0.015653	0.002106
ACE	0	0
ACKR2	0	0
ACSL3	0	0
ADGRG3	0	0
ADRA	0	0
AGT	0	0
AICDA	0	0
ALOX12	0	0
ALOX15	0	0
BATF	0	0
BDKRB1	0	0
BDKRB2	0	0
BLK	0	0
BPI	0	0
C2	0	0
C5	0	0
CARD11	0	0
CARD16	0	0
CASP5	0	0
CCL1	0	0
CCL13	0	0
CCL15	0	0
CCL16	0	0
CCL17	0	0
CCL19	0	0

CCL20	0	0
CCL22	0	0
CCL23	0	0
CCL25	0	0
CCL26	0	0
CCL27	0	0
CCL7	0	0
CCL8	0	0
CCR10	0	0
CCR4	0	0
CCR6	0	0
CCR7	0	0
CCR8	0	0
CCR9	0	0
CCRL2	0	0
CD19	0	0
CD1E	0	0
CD2	0	0
CD209	0	0
CD244	0	0
CD247	0	0
CD38	0	0
CD40	0	0
CD40LG	0	0
CD6	0	0
CD70	0	0
CD79A	0	0
CD79B	0	0
CD80	0	0
CD8A	0	0
CD8B	0	0
CHUK	0	0
CLFA	0	0
CLFB	0	0
CRP	0	0
CSF2	0	0
CSF3	0	0
CSGB	0	0
CTSG	0	0
CUPA1	0	0
CUPA4	0	0
CX3CL1	0	0
CXCL10	0	0
CXCL11	0	0
CXCL13	0	0

CXCL17	0	0
CXCL6	0	0
CXCR3	0	0
CXCR5	0	0
CXCR6	0	0
CYP2E1	0	0
DEFB103A/B	0	0
DHX58	0	0
EBI3	0	0
ELANE	0	0
EOMES	0	0
EPHX2	0	0
FAM30A	0	0
FASLG	0	0
FBXO6	0	0
FCRL2	0	0
FCRL4	0	0
FLGM	0	0
FLIA	0	0
FNBA	0	0
FNBB	0	0
GAB2	0	0
GATA3	0	0
GBP1	0	0
GZMB	0	0
HAMP	0	0
HDC	0	0
HLA-DOB	0	0
HLA-DQB1	0	0
HSD11B1	0	0
ICAA	0	0
ICAB	0	0
ICAC	0	0
ICOS	0	0
ICOSLG	0	0
IDO1	0	0
IFNA1/13	0	0
IFNA14/16	0	0
IFNA2	0	0
IFNA5	0	0
IFNB1	0	0
IFNG	0	0
IFNK	0	0
IFNL1	0	0
IFNL2/3	0	0

IFNL4	0	0
IFNLR1	0	0
IFNW1	0	0
IGHD	0	0
IGHE	0	0
IKBKE	0	0
IL10	0	0
IL11	0	0
IL11RA	0	0
IL12A	0	0
IL12B	0	0
IL12RB1	0	0
IL12RB2	0	0
IL13	0	0
IL13RA2	0	0
IL15	0	0
IL15RA	0	0
IL17A	0	0
IL17B	0	0
IL17C	0	0
IL17D	0	0
IL17F	0	0
IL17RB	0	0
IL17RC	0	0
IL17RD	0	0
IL17RE	0	0
IL18BP	0	0
IL18RAP	0	0
IL19	0	0
IL1A	0	0
IL1F10	0	0
IL1RAPL1	0	0
IL1RAPL2	0	0
IL1RL1	0	0
IL1RL2	0	0
IL20	0	0
IL20RA	0	0
IL20RB	0	0
IL21	0	0
IL21R	0	0
IL22	0	0
IL22RA1	0	0
IL22RA2	0	0
IL23A	0	0
IL24	0	0

IL25	0	0
IL26	0	0
IL27	0	0
IL2RA	0	0
IL3	0	0
IL31	0	0
IL31RA	0	0
IL34	0	0
IL36A	0	0
IL36B	0	0
IL36G	0	0
IL36RN	0	0
IL37	0	0
IL3RA	0	0
IL4	0	0
IL5RA	0	0
IL7	0	0
IL9	0	0
IL9R	0	0
IRF3	0	0
ITGAE	0	0
ITGB7	0	0
ITK	0	0
ITLN1	0	0
KIR2DL3	0	0
KIR3DL1/2	0	0
KLRD1	0	0
LAG3	0	0
LAMP3	0	0
LCK	0	0
LECA	0	0
LECB	0	0
LIF	0	0
LTC4S	0	0
MAP3K5	0	0
MKNK1	0	0
MS4A2	0	0
MX1	0	0
NCR1	0	0
NCR3	0	0
NFATC4	0	0
NLRC5	0	0
NOS2	0	0
NTNG2	0	0
OAS3	0	0

OASL	0	0
P2RX7	0	0
PDCD1	0	0
PDCD1LG2	0	0
PELI2	0	0
PIK3R3	0	0
PIK3R6	0	0
PLG	0	0
PNOC	0	0
PRF1	0	0
RASGRP1	0	0
RELB	0	0
RGMA	0	0
RPS6KB1	0	0
RUNX3	0	0
SH2D1A	0	0
SPIB	0	0
STAT4	0	0
TCF7	0	0
TCL1A	0	0
TGFB2	0	0
TIFA	0	0
TIGIT	0	0
TLR3	0	0
TMEM140	0	0
TMPRSS2	0	0
TNF	0	0
TNFRSF17	0	0
TNFRSF18	0	0
TNFRSF4	0	0
TNFRSF9	0	0
TNFSF18	0	0
TNFSF4	0	0
TNFSF9	0	0
TRAT1	0	0
TRIM5	0	0
TRIM6	0	0
TXK	0	0
ULK2	0	0
XCL1/2	0	0

Figure S1: Volcano plot of infected bone (n=4) versus non-infected control group (n=16).
Volcano plot identifying differentially expressed genes (DEGs) in infected bone biopsy samples (n=4) versus non-infected control group (n =16). The x-axis reports the Log2FoldChange, and the y-axis the -Log10 of the p-adjusted. Genes with a Log2FoldChange > ¦1¦ and -log10 (p-adjusted) > 1.3 (equivalent to p-adjusted < 0.05) were considered as differentially expressed.

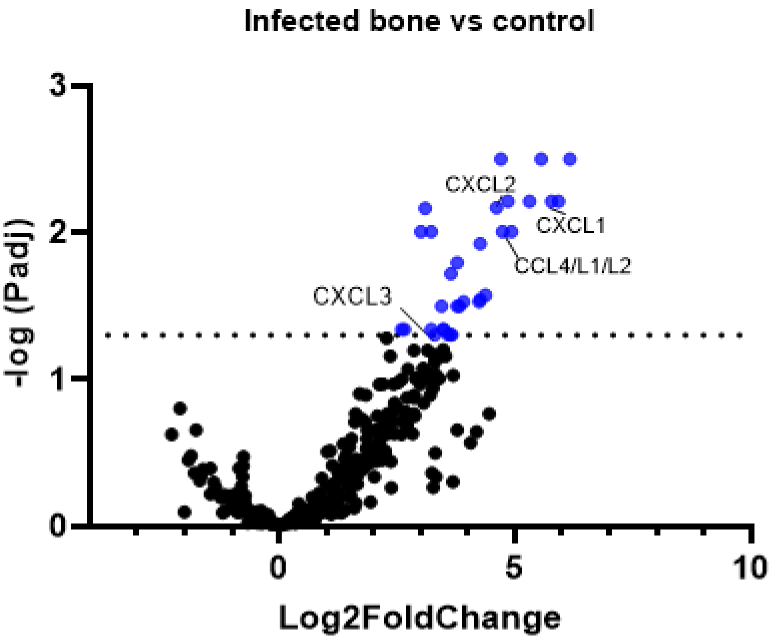

Table S8: Infected tissue samples (n=6) versus a non-infected control group (n=16). P-values were adjusted using the Benjamini–Hochberg false discovery rate (FDR). Significant genes are indicated in bold. (-log (p-value > 1.3)).

Symbol	log2FoldChange	-log(p-value)
CD36	-3.039765	2.112049
CXCL14	-3.481191	1.769244
WIPI1	2.239695	1.659197
CCL24	-2.102570	1.613062
RSAD2	1.601118	1.558765
KLRC1	1.984893	1.545138
CD45RB	1.513440	1.517921
CTLA4	1.762686	1.514869
CASP3	2.748280	1.490111
IL23R	2.114582	1.391263
ADORA2A	2.236326	1.378640
GBP4	1.146274	1.315423
IL2	2.389094	1.211725
GBP5	1.990955	1.164001
C3	-2.339972	1.135660
SELE	1.918295	1.085550
CCL28	1.232661	1.081870
CTSW	1.050626	1.078101
HCST	-1.594623	1.048213
LAMP1	-0.981365	1.022762
NKG7	0.956670	1.021397
IL33	-1.913843	0.986741
SCARB2	-1.221387	0.954399
AIM2	1.186527	0.938290
IFIH1	1.228819	0.929479
GZMA	1.592110	0.918610
TNFRSF25	1.388565	0.916475
TAP2	0.787779	0.907208
CD3G	1.571373	0.895091
IRF1	1.321654	0.888044
NFATC2	1.186362	0.877010
CD69	1.852098	0.849590
NOD2	1.760311	0.849511
PTGER2	0.882074	0.790947
NPC2	-1.009500	0.779460
CDH1	1.085136	0.777295
HERC5	1.384519	0.777276
IFIT2	1.205549	0.757004

SORT1	-1.419920	0.740882
TRIM56	0.983952	0.740168
CD68	-1.155524	0.728282
CXCL5	1.799329	0.725131
XCR1	1.103691	0.716930
CD274	1.046410	0.708348
CCL11	1.304855	0.707610
MARCO	1.482302	0.666638
KLRK1	0.686155	0.663092
IFIT3	1.038919	0.659684
ZAP70	0.914883	0.642574
CEACAM3	1.155711	0.637291
IL5	1.202940	0.618140
CXCR4	1.322633	0.615009
TRIM33	0.663389	0.610955
IRAK4	0.587022	0.599908
TNFRSF10B	1.253341	0.586526
CX3CR1	0.881879	0.581324
GUCY1B1	1.277781	0.580668
IFI27	1.170274	0.576969
CASP10	1.082149	0.568855
REL	1.095924	0.568829
TRAC	1.219556	0.567372
ISG15	1.208587	0.564995
RBPJ	0.910363	0.551587
CARD17	1.163039	0.550858
HPGD	-1.138976	0.545726
TRAF6	0.896360	0.542530
TLR8	0.489615	0.538762
GSTM4	-0.931222	0.532701
PTGS2	1.868144	0.527903
TLR7	0.512112	0.526069
NLRP1	0.499954	0.525443
RACK1	-0.687643	0.524474
CXCL12	-1.352663	0.520950
IRF9	1.007342	0.516481
ULK1	0.466318	0.513188
LITAF	1.111074	0.512792
LGALS3	-0.909872	0.508867
TLR5	1.010569	0.508659
CCR3	0.783232	0.506890
NAMPT	1.361309	0.505623
PTPN4	0.754888	0.501320
PARP9	0.941106	0.499574
TLR9	0.657371	0.497970

CCL18	2.589478	0.497751
IL1RAP	1.180813	0.492366
HAVCR2	-0.810966	0.489916
CCR2	0.996940	0.484252
IRF7	0.957852	0.478439
LRG1	1.322371	0.475769
OSM	2.048730	0.472548
DDAH2	-0.773852	0.468533
SMAD3	-0.969727	0.463751
KRAS	0.917806	0.461044
IL2RB	0.909862	0.459468
MEFV	1.219913	0.458284
RIPK2	0.799975	0.454923
MAF	-0.860958	0.454825
NOTCH1	0.932501	0.450481
CD3E	0.913150	0.449198
HK3	0.704544	0.449139
IFNA8	0.989558	0.447134
CXCL8	1.966764	0.446570
GNS	-0.912625	0.445530
IL2RG	0.852209	0.442292
CSF2RB	0.938599	0.439547
IFNA4/7/10/17/21	0.915478	0.438449
PSTPIP1	0.630373	0.435902
LTF	1.263034	0.430505
TAP1	0.653192	0.426286
CXCR2	0.751294	0.424226
FCGRT	-0.772250	0.423465
LIMK2	1.186260	0.421020
NGLY1	0.731236	0.419927
MLKL	0.684498	0.419758
PYCARD	0.713223	0.414344
FURIN	0.813857	0.413310
MT2A	-0.810911	0.412502
CD276	-1.047581	0.411355
MME	-0.924764	0.410461
BCL3	1.098690	0.404317
JUNB	0.882343	0.404188
IFIT1	0.751860	0.400963
PTGER4	0.684498	0.399293
NCF1	0.934852	0.397314
HCK	0.783181	0.390786
CD14	0.749175	0.389505
ITGB2	-0.787488	0.389311
PIK3R5	1.088497	0.381111

TRIM25	0.942515	0.379468
TLR6	0.845653	0.378886
IL7R	0.655146	0.374955
IRF4	0.622765	0.373863
LANCL1	-0.814308	0.372451
CXCL1	1.440744	0.371411
FPR2	1.068354	0.366457
IL6R	0.846978	0.364722
GBP2	0.630975	0.363346
ACKR4	0.760812	0.361954
FCGR3A/B	0.716689	0.359343
TLR1	0.585703	0.356822
CALM1	-0.536149	0.355134
VCAM1	-0.912867	0.355102
MYC	-0.796908	0.353972
NFKB1	0.877317	0.352947
APEX1	-0.589226	0.346526
JAK3	0.899141	0.346228
TPSAB1/B2	-0.879936	0.340986
CTSL	-0.953619	0.335042
PRDM1	0.608152	0.334202
ADGRE5	0.787436	0.329789
DDOST	-0.605515	0.328961
CD44	0.686727	0.325849
ATG13	0.334387	0.325832
PSMB9	0.579345	0.325322
DDX5	0.569856	0.325030
TIMP2	-0.763505	0.322797
MS4A4A	-0.925169	0.321545
KDM6B	0.909745	0.318000
TRAM1	-0.509317	0.317065
CD3D	0.692491	0.314846
DNAJC10	0.411814	0.314207
CEBPB	0.507021	0.314043
HLA-B	0.565140	0.311137
STAT1	0.694756	0.311009
CD81	-0.666362	0.307869
ZBP1	0.830075	0.306444
CUL1	0.535652	0.305535
NLRC4	0.612213	0.305053
NDUFS8	-0.751047	0.304957
VAMP3	-0.641923	0.303242
JUN	-0.662362	0.300756
PTK2B	0.646613	0.300148
IFNA6	0.735561	0.299612

CSF2RA	0.551249	0.297598
EVL	-0.685787	0.295434
CD45RA	0.638769	0.295046
SOCS3	0.907903	0.294757
PLAT	-0.712942	0.294316
SIGIRR	0.343608	0.293716
IL1B	1.490908	0.291205
CGAS	0.352789	0.288516
CBLB	-1.126344	0.287682
MDFIC	-0.759613	0.287435
GUCY1A1	0.619728	0.286657
SOD1	-0.559902	0.283531
HSP90AB1	-0.482789	0.281967
CTSZ	-0.681990	0.277845
IGFBP7	-0.740447	0.276931
RNF114	-0.554200	0.274739
FOXP3	0.488747	0.274018
GSK3B	0.524137	0.272351
HMGB1	0.324389	0.271902
SMAD5	0.491853	0.271447
CTSA	-0.610841	0.269159
GNLY	0.731652	0.268799
RB1CC1	0.516250	0.266296
CCL14	-0.709781	0.261135
CXCR1	0.654406	0.259968
PIK3CD	0.588741	0.259803
SELENOS	-0.476438	0.259423
BCL2L1	0.722204	0.258384
FOS	0.716438	0.258281
SEM1	-0.692101	0.258117
ETS1	0.620859	0.257261
THOP1	-0.602496	0.256959
CASP4	0.743735	0.254607
PIK3CA	0.467505	0.252691
PDHB	-0.963674	0.251842
BCL2	0.529619	0.251166
FCGR2A	0.675446	0.251066
RNF135	0.238312	0.249351
IFITM1	0.520215	0.247670
LYN	0.767382	0.246780
TNFSF13B	0.634431	0.246633
MAP1LC3A	-0.620019	0.245722
IFITM2	0.621023	0.244815
TYROBP	-0.654508	0.243966
PSAP	-0.508316	0.242030

CD28	0.562936	0.237123
TBX21	0.450512	0.236136
MAP3K1	0.541019	0.235335
LCN2	0.720314	0.235180
OAS2	0.572023	0.233949
LILRB2	0.566347	0.230438
CBFB	-0.952322	0.230155
RIPK3	0.338802	0.230115
MVP	-0.856472	0.229622
CDK4	-0.567041	0.228826
IL10RA	0.444325	0.228689
TRAF3	0.695607	0.227845
AKT1	-0.925470	0.227739
NOX1	0.544321	0.225134
THBS1	-0.498385	0.223585
CD84	-0.584963	0.223505
XAF1	0.574315	0.222273
SMAD4	0.361291	0.222010
PIK3CG	0.519671	0.221990
MAP2K4	-0.448871	0.220447
TXNIP	-0.464695	0.217554
NAE1	-0.386326	0.216144
LTB	0.532495	0.215996
CASP8	0.502258	0.215780
ACSL4	0.478972	0.215416
FAS	0.460036	0.214451
DTX3L	0.324854	0.214399
DDX58	0.460355	0.213856
BCR	0.241478	0.213447
PANX1	-0.555736	0.212858
ATG10	0.459432	0.212287
ACKR3	0.568219	0.212114
HLA-E	-0.940421	0.211949
IL1R1	-0.450502	0.209952
TLR2	0.582900	0.209627
GLB1	-0.366017	0.209181
TCIRG1	-0.435946	0.206404
TAB1	-0.374165	0.206351
CXCL9	0.513976	0.206107
ACVR1	-0.497500	0.205652
CD27	0.546073	0.205064
LAMP2	-0.505550	0.204000
TGFB3	-0.291386	0.203138
MAFB	-0.482612	0.203136
TRIM21	0.403289	0.202966

FGR	0.604862	0.201045
ADAR	0.437911	0.200514
AP1M1	-0.842506	0.200454
TRAF2	-0.424315	0.200404
LDHB	-0.457964	0.198398
FCGR1A/B	0.527932	0.197675
MAP3K8	0.500969	0.197462
JAK2	0.516164	0.196960
TBK1	0.545434	0.196371
STAT5B	0.330598	0.195976
ALOX5	0.515737	0.191747
CD4	-0.463201	0.191698
XBP1	0.457402	0.190365
OAS1	-0.299210	0.190360
ARRB2	0.575932	0.188298
GCA	0.526273	0.186297
HLA-DRA	0.403093	0.185701
FCAR	0.603435	0.185564
CR1	0.613114	0.183147
VWF	-0.414126	0.180561
AKT3	0.407353	0.177699
DNAJA2	-0.411762	0.176456
CCL5	0.375491	0.175492
TANK	0.488286	0.175482
CD86	0.482601	0.175346
APBB1IP	0.457023	0.175344
MARCKS	-0.482999	0.175303
FPR1	0.583031	0.171658
PLAUR	0.727404	0.171563
RPS6KA3	-0.413815	0.171470
BST2	-0.403669	0.170722
BECN1	0.259051	0.170668
GK	0.650750	0.169131
RASGRP4	0.391573	0.168472
GPX7	0.365573	0.167696
MIF	-0.237633	0.167537
PLEKHA1	-0.253574	0.166347
DEFA4	0.420468	0.166314
UBE2N	-0.343532	0.165934
MSRA	-0.363322	0.165735
TNFRSF1A	0.434567	0.164664
SLC2A3	0.650711	0.162857
NCF2	0.548855	0.161353
GLA	-0.244723	0.160914
IL16	0.320392	0.160816

PRKCSH	-0.323614	0.160408
CFLAR	0.361768	0.159989
IGHG	-1.072036	0.158296
IL6ST	-0.325129	0.158245
LCP2	0.593395	0.158155
PTPRC	0.412829	0.156847
ATF6	-0.709444	0.156430
CD22	0.431666	0.156245
NT5E	0.211462	0.155519
ITGAL	0.389327	0.153889
ITPR3	0.193942	0.152455
APP	-0.357218	0.152435
YWHAQ	-0.294305	0.151917
CCL4/L1/L2	0.597863	0.151485
MYD88	0.364098	0.151008
KLRB1	0.434021	0.150709
PPIA	-0.212096	0.148284
RHOG	0.331747	0.148074
IRAK3	0.455930	0.147916
MAP2K3	0.391763	0.147847
JAML	0.418423	0.147084
EIF3F	-0.262364	0.145756
FYN	-0.658097	0.144460
KPNB1	-0.418137	0.143974
IL13RA1	-0.257496	0.143385
DEFA1	0.686973	0.142442
RAB31	-0.724400	0.141612
CD59	-0.321552	0.141567
IL18R1	0.455511	0.141082
TLN1	-0.290630	0.140995
PRKCD	0.414482	0.140027
NFATC3	0.545336	0.139524
IL4R	0.330698	0.139510
SELL	0.400593	0.138468
ACSL1	-0.653707	0.136985
GZMH	0.346176	0.136721
MAPK13	-0.298341	0.136463
IL32	0.394813	0.136096
IFI44	0.322453	0.133785
MS4A1	0.367187	0.133755
C1QBP	-0.297243	0.133458
HLA-DQA	0.342219	0.130898
ICAM3	0.250078	0.130694
CASP1	0.339351	0.128963
PFKFB3	0.440695	0.126442

NLRP3	0.411669	0.126142
VRK3	-0.447719	0.125345
ATF4	-0.218841	0.124787
CBL	0.346699	0.123874
PSEN1	0.338841	0.123309
PIK3CB	0.325095	0.123293
SOCS1	0.318448	0.123147
LAT2	0.356467	0.122099
RAB5C	-0.528365	0.122059
IGHM	0.560582	0.120497
NFKB2	0.387730	0.120099
APOBEC3G	0.324994	0.119600
MAPK8	0.237039	0.118860
TRIM22	-0.286713	0.118355
NFE2L2	0.317074	0.117713
CCR1	0.386302	0.117510
DERL1	-0.348234	0.117181
MCL1	0.327767	0.115893
S100A12	-0.398903	0.114735
TGFBR2	-0.232985	0.114623
PELI1	0.229222	0.110902
HLA-A	0.247188	0.110244
EIF2AK2	-0.253018	0.109884
PTPN6	0.295541	0.109358
NFATC1	0.274895	0.108086
CAP1	-0.253961	0.107416
PLIN4	-0.284506	0.107364
NRAS	-0.263034	0.106054
BNIP3	-0.267952	0.105999
TLR4	0.245242	0.104827
PLAU	0.501876	0.104335
MAPK14	0.185257	0.104155
ITGAX	0.359333	0.103652
IFI6	0.281657	0.103426
WAS	0.310376	0.102899
CYSTM1	0.254648	0.102860
F5	-0.260709	0.102264
STAT2	-0.245112	0.101409
IKBKB	-0.270089	0.101072
NCF4	0.316182	0.101035
ACOX1	0.216054	0.100191
PSMB8	0.113238	0.100173
LCP1	-0.349332	0.100127
HLX	0.206919	0.099986
LRRK2	0.260813	0.098042

MAP3K7	-0.134930	0.096975
STAT5A	-0.281397	0.096745
SUGT1	-0.229225	0.095842
HLA-DMA	-0.274598	0.093892
SPI1	0.273951	0.093269
SP100	0.283005	0.093082
TPP1	-0.189223	0.091777
ATG3	0.275609	0.090817
CD163	-0.250162	0.090789
KIR2DL1	0.263034	0.090318
TBXAS1	0.282533	0.090279
C3AR1	0.319439	0.090188
AKT2	-0.245112	0.089307
HLA-C	0.181926	0.088444
PLCG1	-0.240059	0.087208
SAMHD1	-0.187098	0.086994
TAB2	0.260251	0.086988
CRK	0.176878	0.084657
IRAK1	-0.235430	0.083675
HLA-DRB	0.173221	0.081922
GBA	-0.229417	0.080356
CCL2	0.255559	0.079515
UBA52	0.170687	0.078307
NEU1	-0.151485	0.078006
RELA	0.163499	0.077870
MAP2K2	-0.157971	0.077611
CD45R0	0.250344	0.077173
LEF1	0.245625	0.076853
ATG12	0.141715	0.076625
STT3B	-0.222392	0.076441
PAK1	-0.245112	0.076308
MTOR	0.174498	0.075486
LILRA5	0.240474	0.074404
PRKCQ	0.129283	0.073814
RBCK1	0.203348	0.072103
OS9	-0.135111	0.071531
LTBR	0.178122	0.070246
RPS6KA1	-0.198462	0.069669
DDIT3	-0.338435	0.069442
LTA4H	0.157809	0.068583
HLA-DPB1	0.204750	0.067065
HSP90AA1	0.120992	0.066336
IL17RA	0.132772	0.064564
MAPK1	0.127709	0.064137
RAC2	0.215597	0.063466

APOL6	0.197511	0.063237
LILRA3	0.128849	0.062654
HLA-DMB	-0.181306	0.062535
HMOX1	-0.375327	0.062417
C5AR1	0.210383	0.061648
ATP6V0D1	-0.163685	0.059083
IL10RB	0.169925	0.058986
MAPK9	0.209835	0.058535
TYK2	0.092446	0.057919
CXCL16	-0.184083	0.057221
TOLLIP	0.144390	0.057094
GADD45B	-0.190814	0.056918
PECAM1	0.137410	0.056847
CTSS	-0.142855	0.056214
BCL6	0.194037	0.055828
ERN1	0.184798	0.055472
PIK3R4	0.146841	0.054891
RNASEL	-0.130156	0.054600
IFITM3	0.121278	0.054163
CCNC	-0.139884	0.053732
STAT3	0.162299	0.053617
CSF1R	-0.138828	0.049157
PRKCA	0.139064	0.048541
RNF31	-0.116858	0.047586
CCR5	0.127016	0.046753
JAK1	-0.087698	0.045503
MAP2K7	-0.128733	0.045478
IFI16	-0.132052	0.045363
CSF3R	-0.279666	0.045224
IL18	0.092849	0.044689
MS4A7	-0.151004	0.044654
NEO1	0.089531	0.044382
ATF2	-0.110369	0.044360
MRC1	-0.120076	0.044168
SYK	0.097321	0.042176
SERPINA1	0.237325	0.042084
IL1RN	0.281367	0.041644
ATP6AP2	-0.176183	0.041377
NFAT5	0.097413	0.041235
AIF1	0.094062	0.039886
IL1R2	0.139789	0.038949
IKBKG	-0.083056	0.038924
PLEK	-0.224056	0.038085
PLCG2	0.102238	0.036651
PARP1	0.091177	0.036471

CREBBP	-0.085370	0.035003
TGFB1	-0.063156	0.034394
IFNGR2	0.105731	0.034184
SOD2	-0.141761	0.033825
TCN2	0.101086	0.033659
ITGAM	0.096463	0.033239
DYSF	0.097337	0.031710
TNFSF10	0.116206	0.031036
AP1S2	0.144542	0.030169
SP1	0.066832	0.030060
LAT	-0.074001	0.029144
ATG7	-0.110793	0.029081
RIPK1	-0.048656	0.027504
ALPL	-0.077909	0.025338
EGLN1	-0.052128	0.025013
SSR1	-0.064925	0.024983
ALOX5AP	-0.137950	0.024653
RAB7A	0.052790	0.024542
VSIR	-0.077689	0.024060
CSF1	-0.063726	0.023991
HLA-DPA1	0.058837	0.023373
DIABLO	0.048910	0.020744
MAVS	0.053111	0.020674
TXN	-0.045765	0.019745
FOXO1	-0.051225	0.019628
ALPK1	-0.051530	0.018859
MAP3K3	-0.037112	0.018481
ATG4A	-0.043943	0.018371
IL27RA	0.034270	0.017802
STRAP	0.084944	0.017652
EIF2AK3	0.036951	0.017035
PRCP	-0.048582	0.016801
RAF1	0.050937	0.016581
ATM	0.051095	0.015420
LILRA6	0.033167	0.014496
ANPEP	0.040095	0.014338
CCL3/L1/L3	-0.088862	0.014172
PSMB10	-0.033167	0.014012
ENTPD1	0.038680	0.013540
MAPKAPK2	0.033640	0.013482
IL6	-0.050374	0.013297
IFI35	0.038013	0.013265
CCL21	-0.080218	0.012970
CPA3	-0.040519	0.012672
HSP90B1	-0.031499	0.012460

PXN	-0.027579	0.011979
PIK3C3	0.014470	0.010933
IGHA	0.060130	0.010814
UBE2L6	0.023300	0.009697
AHR	0.023958	0.009215
STING1	0.021349	0.008571
IFNAR2	-0.025385	0.008401
ATP6V1B2	-0.021069	0.006477
IFNAR1	0.014571	0.006399
SIGLEC5	-0.025095	0.005471
SLC11A1	0.027568	0.005466
CXCL3	-0.015026	0.004239
SIRPA	0.013575	0.004170
VEGFA	0.021791	0.003787
CXCL2	0.015732	0.003392
STAT6	0.005510	0.002654
MGAM	0.007829	0.002464
AP1G1	-0.000930	0.000507
ACE	0	0
ACKR2	0	0
ACSL3	0	0
ADGRG3	0	0
ADRA	0	0
AGT	0	0
AICDA	0	0
ALOX12	0	0
ALOX15	0	0
BATF	0	0
BDKRB1	0	0
BDKRB2	0	0
BLK	0	0
BPI	0	0
C2	0	0
C5	0	0
CARD11	0	0
CARD16	0	0
CASP5	0	0
CCL1	0	0
CCL13	0	0
CCL15	0	0
CCL16	0	0
CCL17	0	0
CCL19	0	0
CCL20	0	0
CCL22	0	0

CCL23	0	0
CCL25	0	0
CCL26	0	0
CCL27	0	0
CCL7	0	0
CCL8	0	0
CCR10	0	0
CCR4	0	0
CCR6	0	0
CCR7	0	0
CCR8	0	0
CCR9	0	0
CCRL2	0	0
CD19	0	0
CD1E	0	0
CD2	0	0
CD209	0	0
CD244	0	0
CD247	0	0
CD38	0	0
CD40	0	0
CD40LG	0	0
CD6	0	0
CD70	0	0
CD79A	0	0
CD79B	0	0
CD80	0	0
CD8A	0	0
CD8B	0	0
CHUK	0	0
CLFA	0	0
CLFB	0	0
CRP	0	0
CSF2	0	0
CSF3	0	0
CSGB	0	0
CTSG	0	0
CUPA1	0	0
CUPA4	0	0
CX3CL1	0	0
CXCL10	0	0
CXCL11	0	0
CXCL13	0	0
CXCL17	0	0
CXCL6	0	0

CXCR3	0	0
CXCR5	0	0
CXCR6	0	0
CYP2E1	0	0
DEFB103A/B	0	0
DHX58	0	0
EBI3	0	0
ELANE	0	0
EOMES	0	0
EPHX2	0	0
FAM30A	0	0
FASLG	0	0
FBXO6	0	0
FCRL2	0	0
FCRL4	0	0
FLGM	0	0
FLIA	0	0
FNBA	0	0
FNBB	0	0
GAB2	0	0
GATA3	0	0
GBP1	0	0
GZMB	0	0
HAMP	0	0
HDC	0	0
HLA-DOB	0	0
HLA-DQB1	0	0
HSD11B1	0	0
ICAA	0	0
ICAB	0	0
ICAC	0	0
ICOS	0	0
ICOSLG	0	0
IDO1	0	0
IFNA1/13	0	0
IFNA14/16	0	0
IFNA2	0	0
IFNA5	0	0
IFNB1	0	0
IFNG	0	0
IFNK	0	0
IFNL1	0	0
IFNL2/3	0	0
IFNL4	0	0
IFNLR1	0	0

IFNW1	0	0
IGHD	0	0
IGHE	0	0
IKBKE	0	0
IL10	0	0
IL11	0	0
IL11RA	0	0
IL12A	0	0
IL12B	0	0
IL12RB1	0	0
IL12RB2	0	0
IL13	0	0
IL13RA2	0	0
IL15	0	0
IL15RA	0	0
IL17A	0	0
IL17B	0	0
IL17C	0	0
IL17D	0	0
IL17F	0	0
IL17RB	0	0
IL17RC	0	0
IL17RD	0	0
IL17RE	0	0
IL18BP	0	0
IL18RAP	0	0
IL19	0	0
IL1A	0	0
IL1F10	0	0
IL1RAPL1	0	0
IL1RAPL2	0	0
IL1RL1	0	0
IL1RL2	0	0
IL20	0	0
IL20RA	0	0
IL20RB	0	0
IL21	0	0
IL21R	0	0
IL22	0	0
IL22RA1	0	0
IL22RA2	0	0
IL23A	0	0
IL24	0	0
IL25	0	0
IL26	0	0

IL27	0	0
IL2RA	0	0
IL3	0	0
IL31	0	0
IL31RA	0	0
IL34	0	0
IL36A	0	0
IL36B	0	0
IL36G	0	0
IL36RN	0	0
IL37	0	0
IL3RA	0	0
IL4	0	0
IL5RA	0	0
IL7	0	0
IL9	0	0
IL9R	0	0
IRF3	0	0
ITGAE	0	0
ITGB7	0	0
ITK	0	0
ITLN1	0	0
KIR2DL3	0	0
KIR3DL1/2	0	0
KLRD1	0	0
LAG3	0	0
LAMP3	0	0
LCK	0	0
LECA	0	0
LECB	0	0
LIF	0	0
LTC4S	0	0
MAP3K5	0	0
MKNK1	0	0
MS4A2	0	0
MX1	0	0
NCR1	0	0
NCR3	0	0
NFATC4	0	0
NLRC5	0	0
NOS2	0	0
NTNG2	0	0
OAS3	0	0
OASL	0	0
P2RX7	0	0

PDCD1	0	0
PDCD1LG2	0	0
PELI2	0	0
PIK3R3	0	0
PIK3R6	0	0
PLG	0	0
PNOC	0	0
PRF1	0	0
RASGRP1	0	0
RELB	0	0
RGMA	0	0
RPS6KB1	0	0
RUNX3	0	0
SH2D1A	0	0
SPIB	0	0
STAT4	0	0
TCF7	0	0
TCL1A	0	0
TGFB2	0	0
TIFA	0	0
TIGIT	0	0
TLR3	0	0
TMEM140	0	0
TMPRSS2	0	0
TNF	0	0
TNFRSF17	0	0
TNFRSF18	0	0
TNFRSF4	0	0
TNFRSF9	0	0
TNFSF18	0	0
TNFSF4	0	0
TNFSF9	0	0
TRAT1	0	0
TRIM5	0	0
TRIM6	0	0
TXK	0	0
ULK2	0	0
XCL1/2	0	0

Figure S2: Volcano plot of infected tissue biopsy (n=8) versus non-infected control group (n=16). Volcano plot identifying differentially expressed genes (DEGs) in infected tissue samples (n=8) versus controls (n=16). The x-axis reports the Log2FoldChange, and the y-axis the -Log10 of the p-value. Genes with a Log2FoldChange > ¦1¦ and -log10 (p-value) > 1.3 (equivalent to p-value < 0.05) were considered as differentially expressed.

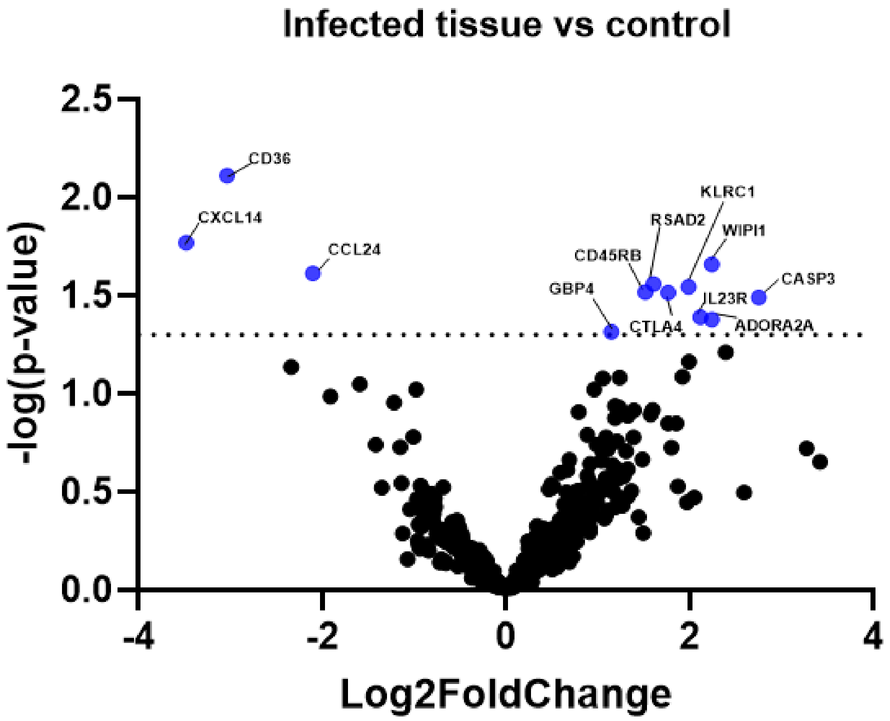

Supplement: The supplement related to this article is available online at https://doi.org/10.5194/jbji-11-161-2026-supplement. [file jbji-11-161-2026-supplement.pdf]
